# ALKBH4 promotes tumourigenesis with a poor prognosis in non-small-cell lung cancer

**DOI:** 10.1038/s41598-021-87763-1

**Published:** 2021-04-21

**Authors:** Kentaro Jingushi, Masaya Aoki, Kazuhiro Ueda, Takahiro Kogaki, Masaya Tanimoto, Yuya Monoe, Masayuki Ando, Takuya Matsumoto, Kentaro Minami, Yuko Ueda, Kaori Kitae, Hiroaki Hase, Toshiyuki Nagata, Aya Harada-Takeda, Masatatsu Yamamoto, Kohichi Kawahara, Kazuhiro Tabata, Tatsuhiko Furukawa, Masami Sato, Kazutake Tsujikawa

**Affiliations:** 1 Laboratory of Molecular and Cellular Physiology, Graduate School of Pharmaceutical Sciences, Osaka University, 1-6 Yamadaoka, Suita, Osaka 565-0871 Japan; 2 Department of General Thoracic Surgery, Graduate School of Medical and Dental Sciences, Kagoshima University, 8-35-1 Sakuragaoka, Kagoshima, Kagoshima 890-8520 Japan; 3 Department of Molecular Oncology, Graduate School of Medical and Dental Sciences, Kagoshima University, 8-35-1 Sakuragaoka, Kagoshima, Kagoshima 890-8544 Japan; 4 Human Pathology, Kagoshima University Graduate School of Medical and Dental Sciences, 8-35-1 Sakuragaoka, Kagoshima City, 890-8544 Japan

**Keywords:** Non-small-cell lung cancer, Oncogenes

## Abstract

The human AlkB homolog family (ALKBH) of proteins play a critical role in some types of cancer. However, the expression and function of the lysine demethylase *ALKBH4* in cancer are poorly understood. Here, we examined the expression and function of *ALKBH4* in non-small-cell lung cancer (NSCLC) and found that *ALKBH4* was highly expressed in NSCLC, as compared to that in adjacent normal lung tissues. *ALKBH4* knockdown significantly induced the downregulation of NSCLC cell proliferation via cell cycle arrest at the G_1_ phase of in vivo tumour growth. *ALKBH4* knockdown downregulated E2F transcription factor 1 (E2F1) and its target gene expression in NSCLC cells. *ALKBH4* and *E2F1* expression was significantly correlated in NSCLC clinical specimens. Moreover, patients with high *ALKBH4* expression showed a poor prognosis, suggesting that ALKBH4 plays a pivotal tumour-promoting role in NSCLC.

## Introduction

*Escherichia coli* AlkB is a 2-oxoglutarate and Fe (II)-dependent dioxygenase that repairs alkylated DNA/RNA nucleotides by catalysing oxidative demethylation^1–3^. In humans, the existence of nine AlkB homolog (ALKBH) family members (ALKBH1–ALKBH9) has been reported^4,5^. Some of the ALKBH molecules recognise various kinds of substrates, including methylated single- or double-stranded DNA or RNA bases^6^. Recently, the high expression of ALKBH molecules, such as ALKBH2^7^, ALKBH3^8–12^ and ALKBH8^13,14^, has been reported to have tumour promoting activity in several cancers.

ALKBH4, one of the ALKBH family proteins, has been reported to be a lysine demethylase of actin in HEK293T cells^15^. Moreover, ALKBH4 has been reported to regulate gene expression or the chromatin state through association with transcriptional factors and chromatin regulation factors in HEK293 cells^16^. Recently, ALKBH4 has been reported to function as a suppressor of metastasis as it binds competitively to WD repeat-containing protein 5 (WDR5) in colon cancer^17^. However, the expression, substrate and biological function of ALKBH4 in other cancers are currently unknown.

Non-small-cell lung cancer (NSCLC) is one of the leading causes of cancer death worldwide. NSCLC accounts for approximately 85% of all lung cancers^18^. NSCLC is divided into adenocarcinoma, squamous cell carcinoma and large cell carcinoma. The most widely recognised genomic alterations in NSCLC include epidermal growth factor receptor (*EGFR*) mutations and echinoderm microtubule-associated protein-like 4-anaplastic lymphoma kinase (EML-ALK) fusion gene mutations. Although, molecular-targeted drugs for the proteins with such genetic mutations, improved the prognosis of NSCLC patients and their continuous usage often induced acquired resistance^19^. Moreover, some NSCLC cases do not present such mutations. Therefore, a novel therapeutic target candidate or approach is eagerly required.

The E2 promoter-binding factor (E2F) family of transcriptional factors consists of E2F1, E2F2, and E2F3. The activation and overexpression of members of the E2F family can contribute to oncogenic transformation of rodent embryonic fibroblasts and to tumourigenesis^20^. E2F1 plays an important role in cell cycle progression, senescence and apoptosis^21–23^. Abnormalities in E2F1 gene expression are found in several human cancers. Retrospective studies of several cancers, including NSCLC, indicate that an upregulated E2F1 expression is frequently associated with high-grade tumours and a poor patient survival prognosis^24–26^.

In this study, we show that the high expression of *ALKBH4* in NSCLC specimens is associated with a poor prognosis. Additionally, we demonstrate the tumour-promoting role of *ALKBH4* via its enzymatic activity in NSCLC cells.

## Results

### ALKBH4 is highly expressed in NSCLC tissues

**Figure 1:**
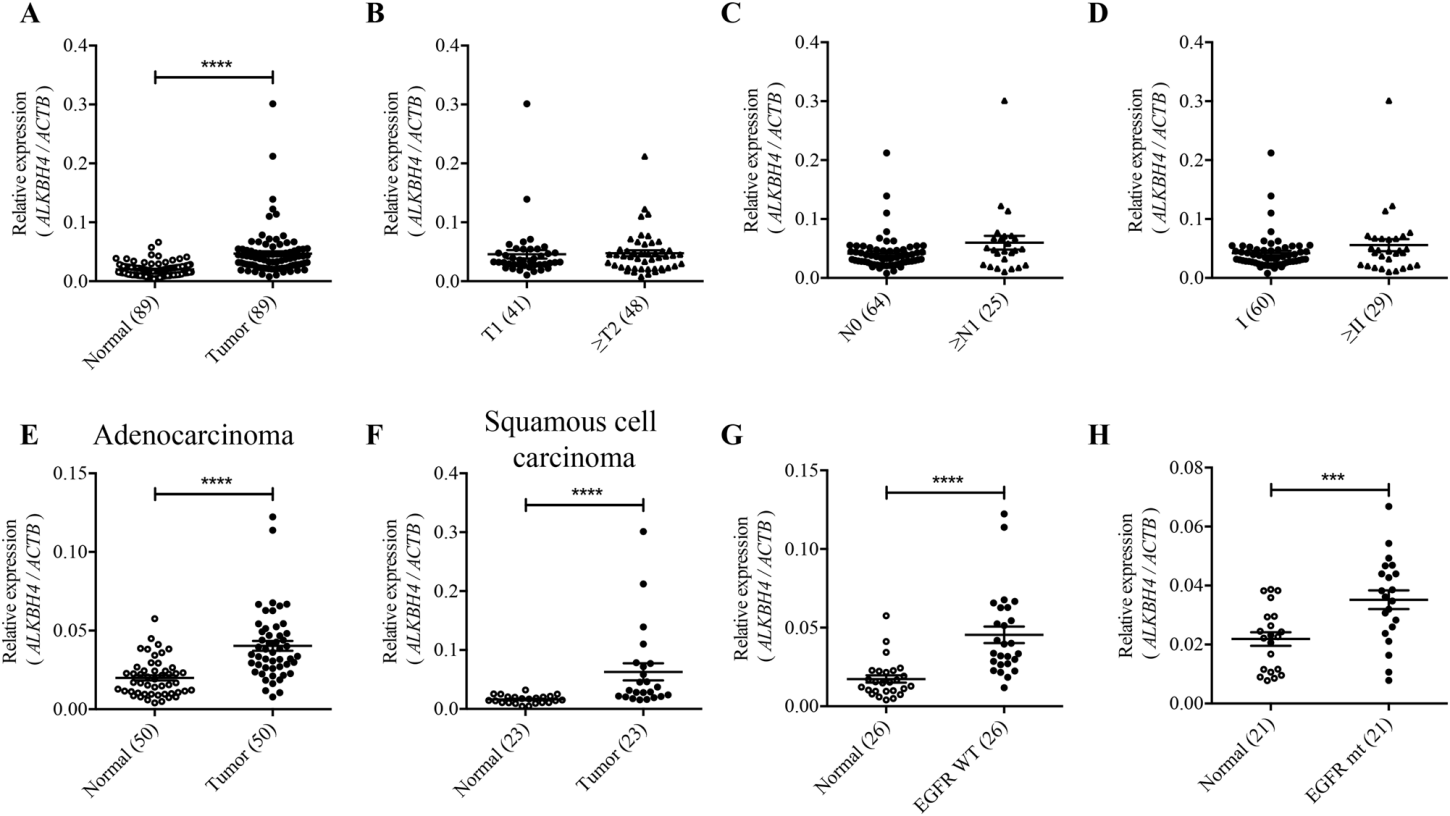
*ALKBH4* is highly expressed in NSCLC. *ALKBH4* expression levels were measured using qPCR and compared between normal and tumour tissues. (**A**) T classification (**B**), N classification (**C**), stages (**D**), histological types (**E,F**), and EGFR mutation (*WT* wild type, *mt* mutation) status (**G,H**) in NSCLC clinical specimens. Relative *ALKBH4* expression normalised to *ACTB* is shown. Data are represented as mean ± S.D. The number of specimens examined is shown in parentheses. ***p < 0.001; ****p < 0.0001 for paired *t*-test (**A**) and Student’s *t*-test (**B**–**H**).

To obtain the *ALKBH4* expression profile, we evaluated *ALKBH4* expression in 89-matched pair NSCLC clinical specimens using qPCR. Compared to adjacent normal lung tissues, *ALKBH4* expression was significantly higher in tumour tissues (Fig. 1A). Although there was no significant difference between either of the tumour stages, node stages (Fig. 1B,C), or pathological stages (Fig. 1D), *ALKBH4* expression was higher in tumour tissues than in normal tissues, regardless of histologic subtypes (Fig. 1E,F). Interestingly, a higher *ALKBH4* expression was detected in tumour tissues than in matched-pair normal tissues, regardless of the presence or absence of epidermal growth factor receptor (*EGFR*) gene mutations (Fig. 1G,H).

### ALKBH4 knockdown reduced cell proliferation by inducing G_1_ phase arrest in NSCLC cells

**Figure 2:**
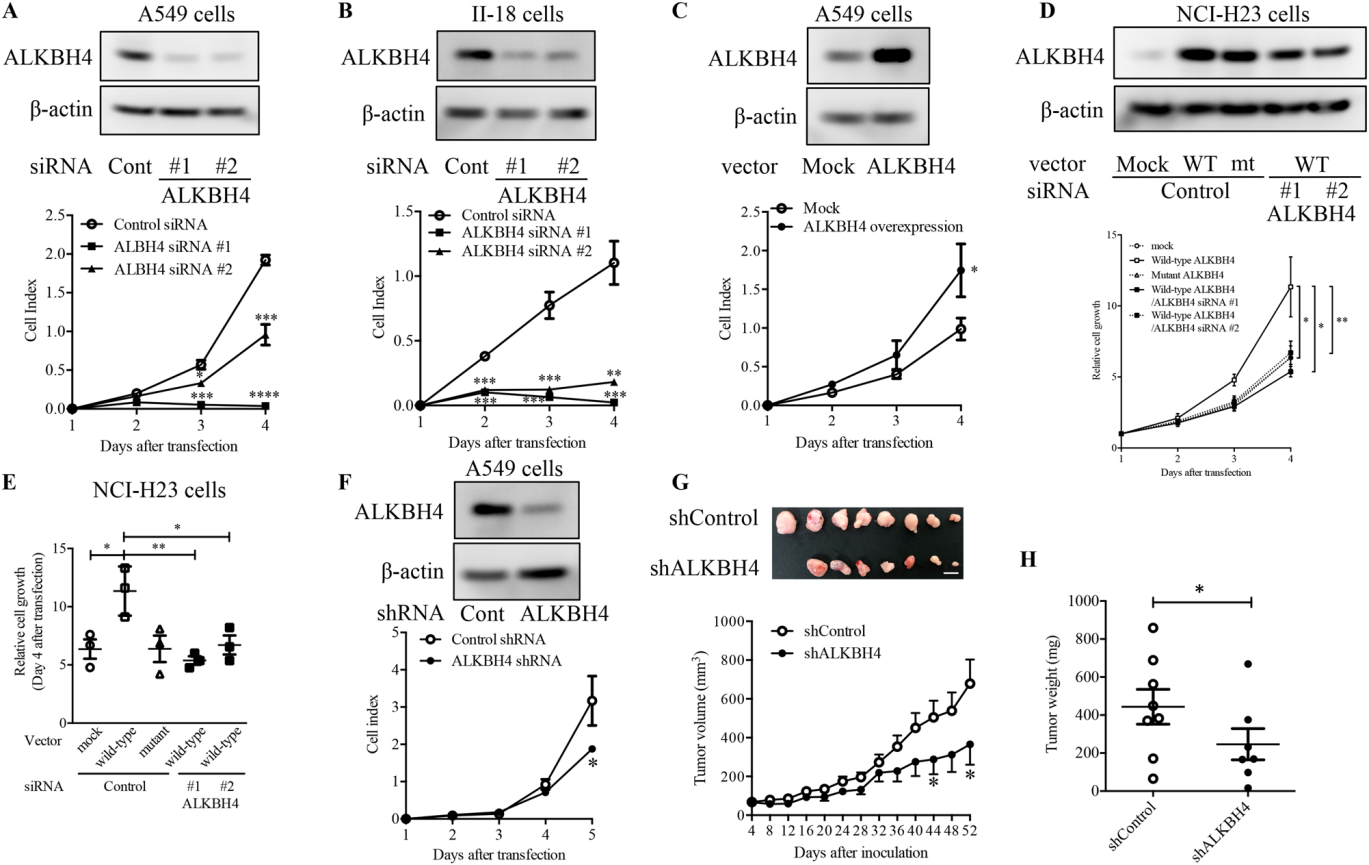
ALKBH4 knockdown suppressed NSCLC cells proliferation. Lysates of A549 (**A**) and II-18 (**B**) cells transfected with two ALKBH4 siRNAs (#1 and #2) or control siRNA, A549 cells transfected with pEB Multi-Neo-ALKBH4 or mock vector (**C**) were subjected to Western blot analysis with anti-ALKBH4 and anti-β-actin antibodies. Uncropped Western blot data are shown in Supplementary Fig. S9. Representative pictures of three independent experiments are shown (upper pictures). The cells which were transfected for 48 h were reseeded on a xCELLigence E-plate and their proliferation was detected using a xCELLigence DP system (lower panels). Degrees of cell proliferation expressed as cell index by the system are the means ± S.D. of three independent experiments (**A**–**C**). (**D**) Lysates of NCI-H23 cells co-transfected with pEB Multi-Neo-ALKBH4 wild-type (WT), pEB Multi-Neo-ALKBH4 mutant (mt), or mock vector and ALKBH4 siRNAs were subjected to western blot analysis with anti-ALKBH4 and anti-β-actin antibodies. Uncropped Western blot data are shown in Supplementary Fig. S9. Representative pictures of three independent experiments are shown (upper pictures). NCI-H23 cells were transfected for 48 h and reseeded in 96-well plates, and their proliferation was measured using the WST-8 assay (lower panels). The relative cell growth data on day 4 are presented in (**E**). The values are presented as the mean ± S.D. for each group. *p < 0.05; **p < 0.01 vs. wild-type ALKBH4 (one-way ANOVA with Bonferroni post-hoc tests). (**F**) A549 cells stably expressing ALKBH4 shRNA or control shRNA were subjected to Western blot analysis with anti-ALKBH4 and anti-β-actin antibodies. Uncropped Western blot data are shown in Supplementary Fig. S9. Representative pictures of three independent experiments are shown (upper pictures). Cell proliferation was detected using a xCELLigence DP system (lower panels). Degrees of cell proliferation expressed as cell index by the system are the means ± S.D. of three independent experiments (lower panels). (**G**) A549 cells stably expressing ALKBH4 shRNA (shALKBH4) and control shRNA (shControl), which are shown in (**F**), were subcutaneously injected into nude mice. The upper picture shows a xenograft tumour from mice inoculated with shControl- or shALKBH4-expressing A549 cells (shControl: n = 8; shALKBH4: n = 7). White scale bar, 1 cm. Tumour volume was calculated by measuring the tumour size every four days (lower panel). *p < 0.05 vs. control (Student’s *t*-test). (**H**) Tumour weight in shControl- and shALKBH4 xenografted mice. The values are presented as mean ± S.D. for each group. *p < 0.05 vs. shControl tumour (Student’s *t*-test).

To investigate the function of ALKBH4 in NSCLC cells, we first evaluated the expression of *ALKBH4* in 22 NSCLC cell lines (adenocarcinoma: 15 cell lines; squamous cell carcinoma: 7 cell lines). Compared to the adjacent normal lung tissues obtained from postoperative tissues of NSCLC patients, all 22 NSCLC cell lines expressed high levels of *ALKBH4* (Supplementary Fig. S1). A549 and II-18 cells with a high *ALKBH4* expression were used for the subsequent knockdown and overexpression experiments. ALKBH4 knockdown using siRNAs significantly suppressed the proliferation of A549 (Fig. 2A) and II-18 cells (Fig. 2B). Conversely, *ALKBH4* overexpression using pEB-ALKBH4 vector significantly promoted the proliferation of A549 cells (Fig. 2C). Also, in the case of NCI-H23 cells, the overexpression of ALKBH4 led to increased proliferation. However, overexpression of a catalytically inactive mutant ALKBH4 (H169A/D171A) had no significant effect on cell proliferation (Fig. 2D,E). Moreover, ALKBH4 knockdown inhibited the effect of overexpression of wild-type ALKBH4 on cell proliferation in NCI-H23 cells (Fig. 2D,E), suggesting that ALKBH4 promotes cell growth via its enzymatic activity. To clarify the tumour-promoting potential of *ALKBH4* expression in vivo, A549 cells stably expressing control or ALKBH4 shRNA were constructed. We confirmed the decreased proliferation of A549 cells transfected with ALKBH4 shRNA, compared with those transfected with control shRNA, in vitro (Fig. 2F). These cells were used as an in vivo xenograft model. Suppression of tumour volume, as well as of tumour weight, was observed in mice xenografted with *ALKBH4*-knockdown A549 cells (shALKBH4), compared with the control cells (shControl) in Fig. 2G,H, respectively. These results suggest that *ALKBH4* might function as a critical tumour promoter in NSCLC cells.

**Figure 3:**
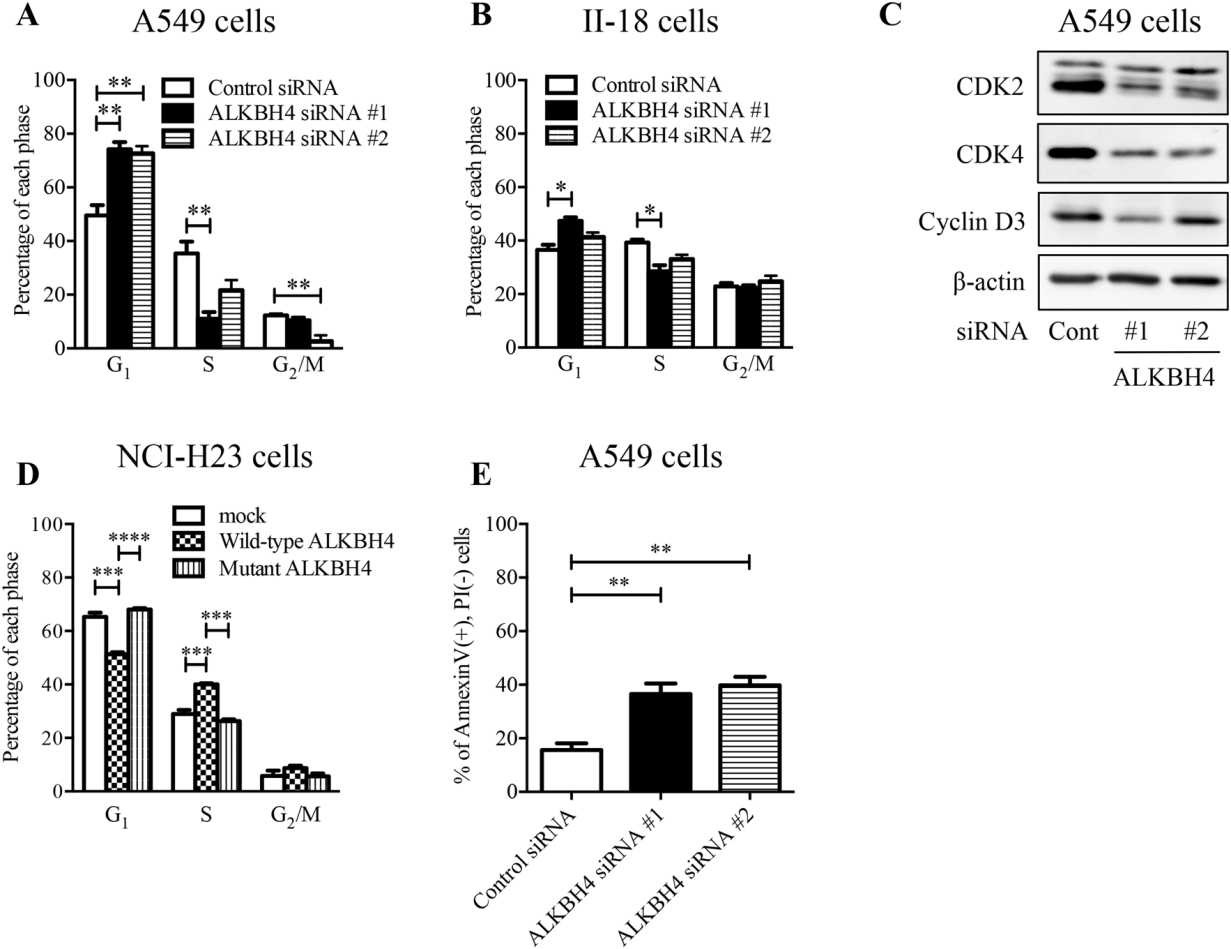
ALKBH4 knockdown induced G1 phase arrest in NSCLC cells. A549 cells (**A**) or II-18 cells (**B**) were transfected with ALKBH4 siRNAs or control siRNA for 48 h and the cell cycle was analysed using a flow cytometer. Values are represented as means ± S.D. of three independent experiments. *p < 0.05; **p < 0.01 vs. control siRNA (One-way ANOVA with Bonferroni post-hoc tests). (**C**) Lysates of A549 cells transfected with ALKBH4 siRNAs or control siRNA were subjected to Western blot analysis with anti-CDK2, anti-CDK4, anti-Cyclin D3, and anti-β-actin antibodies. Uncropped Western blot data are shown in Supplementary Fig. S9. Representative pictures of three independent experiments are shown. (**D**) NCI-H23 cells were transfected with wild-type ALKBH4 or mutant ALKBH4 for 48 h and the cell cycle was analysed using a flow cytometer. Values are represented as means ± S.D. of three independent experiments. ***p < 0.001; ****p < 0.0001 (One-way ANOVA with Bonferroni post-hoc tests). (**E**) A549 cells were transfected with ALKBH4 siRNAs or control siRNA for 48 h and the apoptosis was analysed using a flow cytometer. Values are represented as means ± S.D. of three independent experiments. **p < 0.01 vs. control siRNA (One-way ANOVA with Bonferroni post-hoc tests).

To clarify the function of *ALKBH4* on cell proliferation, we conducted gene array analysis using the total RNA of A549 cells transfected with either ALKBH4 siRNA #1 or control siRNA. *ALKBH4* knockdown affected the expression of 3572 genes (fold-change ≥|1.5|, 1561 upregulated genes, and 2011 downregulated genes) in A549 cells (Supplementary Table S2). Gene ontology enrichment analysis using several databases (Bioplanet, KEGG, and Reactome) revealed that these 2011 genes were mostly associated with cell cycle processes (Supplementary Table S3). Upregulated 1561 genes were associated with p53 signaling pathway and ECM-receptor interaction (Supplementary Table S4). To examine whether the suppression of cell proliferation induced by *ALKBH4* knockdown was due to cell cycle arrest, we performed cell cycle analysis. *ALKBH4* knockdown elevated the ratio of G_1_ phase cells and decreased the ratio of S phase cells in A549 (Fig. 3A) and II-18 cells (Fig. 3B). Moreover, cyclin-dependent kinase 2 (CDK2), cyclin-dependent kinase 4 (CDK4), and cyclin D3, the key proteins for the progression of the G_1_ phase, were markedly downregulated after *ALKBH4* knockdown in A549 cells (Fig. 3C). Conversely, overexpression of wild-type ALKBH4 increased the ratio of S phase cells in NCI-H23 cells (Fig. 3D). Apoptosis analysis showed that ALKBH4 knockdown significantly induced apoptosis in A549 cells compared to control siRNA transfection (Fig. 3E). To further clarify the function of ALKBH4 in the cell cycle, we conducted G_0_-marker assays through β-galactosidase staining. ALKBH4 knockdown had no significant effect on senescence-associated β-galactosidase expression in A549 cells (Supplementary Fig. S2). These results suggest that highly expressed *ALKBH4* promotes cell proliferation via regulation of the progression of the G_1_ phase in NSCLC cells.

Using Gene Expression Profiling Interactive Analysis (GEPIA)^27^, an interactive web server for analysing RNA sequencing expression data of tumours and normal samples from The Cancer Genome Atlas (TCGA) and The Genotype-Tissue Expression (GTEx) database (https://gtexportal.org/home/), we found that a broad range of cancer tissues, including lung adenocarcinoma and lung squamous cell carcinoma, express higher levels of *ALKBH4* mRNA than each corresponding normal tissue (Supplementary Fig. S3A). To examine whether ALKBH4 specifically promotes cell proliferation in NSCLC cells or not, first, we examined the expression of *ALKBH4* in some cancer cell lines (prostate, liver, lung, breast, renal, and pancreatic cancer cell lines). As shown in Supplementary Fig. S3B, *ALKBH4* was highly expressed in many of the cancer cell lines examined. Then, we used MCF7 breast cancer cells to confirm the effect of ALKBH4 siRNAs on cell proliferation. *ALKBH4* knockdown significantly suppressed the proliferation of MCF7 cells (Supplementary Fig. S3C,D). Moreover, *ALKBH4* knockdown elevated the ratio of G_1_ phase cells and decreased the ratio of S phase cells in MCF7 cells (Supplementary Fig. S3E), suggesting that *ALKBH4* plays an important role in the regulation of the proliferation of, not only NSCLC cells, but also other cancer cells.

### ALKBH4 knockdown downregulated E2F1 signalling in NSCLC cells

**Figure 4:**
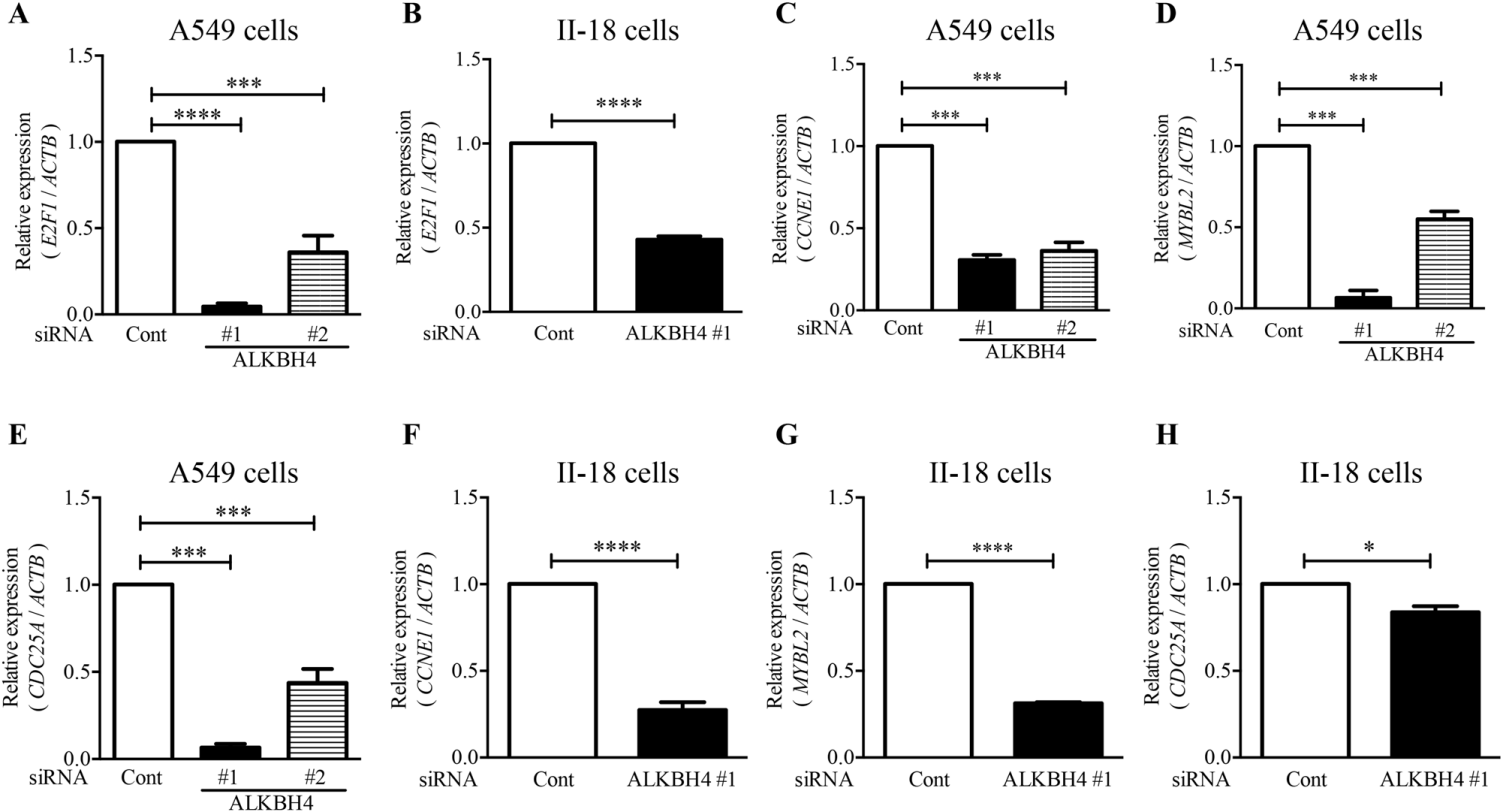
ALKBH4 knockdown downregulated E2F1 and E2F1-target genes in A549 and II-18 cells. A549 cells (**A,C**–**E**) or II-18 cells (**B,F**–**H**) transfected with ALKBH4 siRNAs or control siRNA for 48 h were subjected to qPCR analysis of *E2F1*, *CCNE1*, *MYBL2*, and *CDC25A* expression. The relative expression values, which were normalised according to *ACTB* expression, are represented as mean ± S.D. of three independent experiments. *p < 0.05; ***p < 0.001; ****p < 0.0001 vs. control siRNA. (One-way ANOVA with Bonferroni post-hoc tests for (**A**,**C**–**E**), and Student’s *t*-test for (**B**,**F**–**H**)).

Since *ALKBH4* knockdown induced G_1_ phase arrest in NSCLC cells, we focused on *E2F1* being known as a critical regulator of G_1_/S phase transition. *ALKBH4* knockdown significantly reduced *E2F1* expression in A549 and II-18 cells (Fig. 4A,B, respectively). Enrichment analysis using ChEA database^28^ showed that 215 E2F1-target genes were also downregulated via *ALKBH4* knockdown (Supplementary Table S5). Since phospho-Ser/Thr phosphatase cdc25A (CDC25A), cyclin E1 (CCNE1), and Myb-like protein 2 (MYBL2) have been reported as tumour promoters^29–31^ and are known as cell cycle regulators in NSCLC, we focused on these genes. To verify the results of gene array analysis, the expression of E2F1-target genes was determined using qPCR. *ALKBH4* knockdown significantly downregulated the target genes of E2F1 both in A549 and in II-18 cells (Fig. 4C–H).

**Figure 5:**
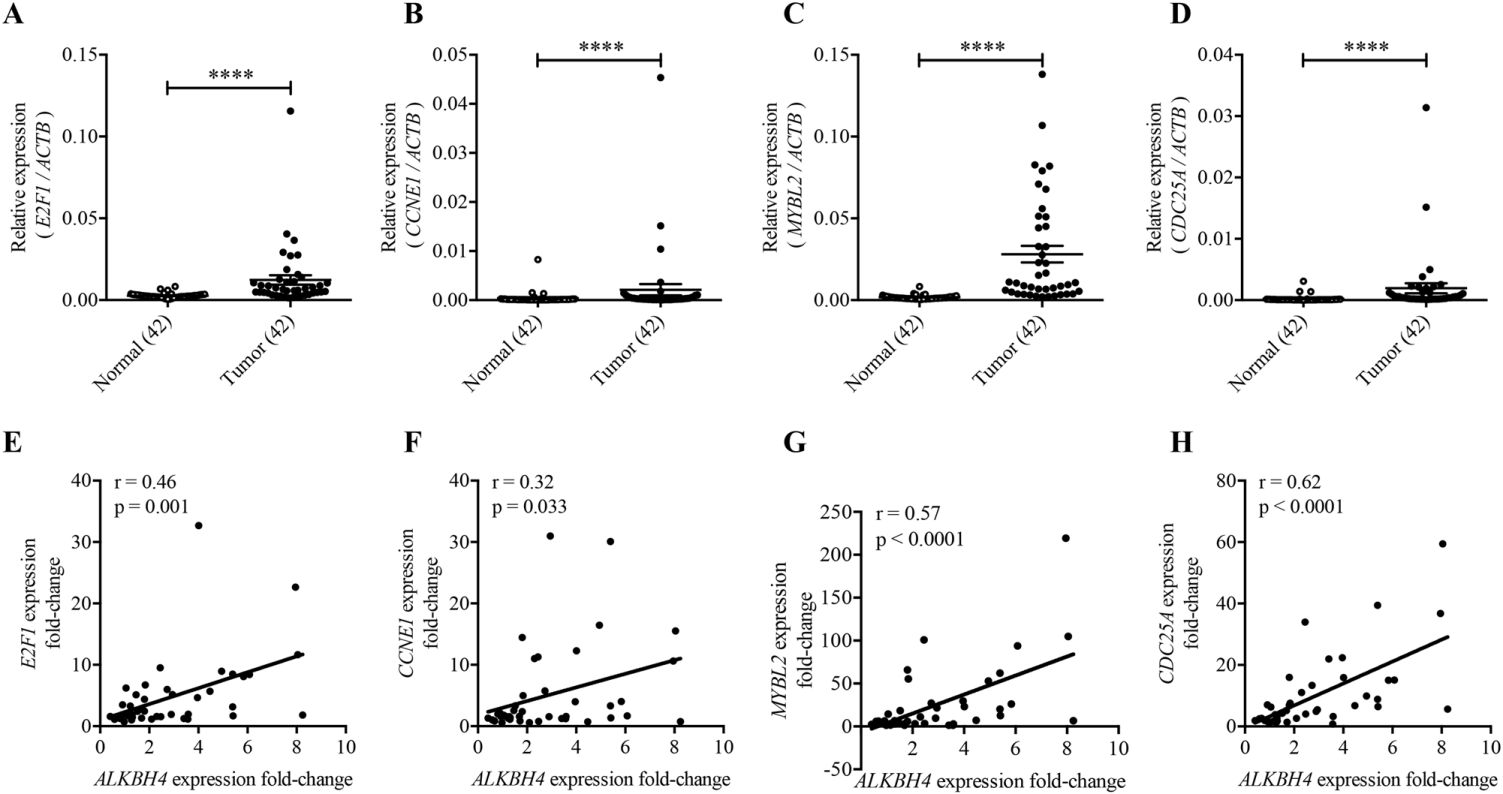
The expression of *ALKBH4* and *E2F1*of E2F1-target genes has a positive correlation in NSCLC clinical specimens. *E2F1* (**A**) and E2F1-target gene ((**B**) *CCNE1*, (**C**) *MYBL2*, and (**D**) *CDC25A*) expression levels were measured using qPCR and were compared between normal and tumour tissues in NSCLC clinical specimens. Relative expression normalised to *ACTB* is shown. Data are represented as means ± S.D. ****p < 0.0001 for paired *t*-test. Correlation analysis was performed with the relative *ALKBH4* expression and the relative *E2F1* expression (**E**), *CCNE1* expression (**F**), *MYBL2* expression (**G**), and *CDC25A* expression (**H**) using 42-matched pairs of NSCLC samples. Pearson correlation analysis was conducted.

The relationship between *ALKBH4* and *E2F1* expression or those of E2F1-target genes was further confirmed in NSCLC specimens. Compared to the normal adjacent lung tissues, the expression of *E2F1* (Fig. 5A) and of its target genes (Fig. 5B–D) was significantly high in tumour tissues. Importantly, there was a positive correlation (r = 0.46, p = 0.001) between *ALKBH4* and *E2F1* expression in NSCLC specimens (Fig. 5E). A significant positive correlation was also observed between *ALKBH4* and each of the E2F1-target genes (Fig. 5F–H). The positive correlation between the expression of *ALKBH4* and *E2F1*, as well as between that of *ALKBH4* and of each of the E2F1-target genes was observed, regardless of the presence or absence of *EGFR* gene mutation (Supplementary Fig. S4A–H). In addition, only stage I, but not late stage (≥ stage II) NSCLC, showed a positive correlation between *ALKBH4* and *E2F1*, or each of the E2F1-target genes (Supplementary Fig. S5A–H). Moreover, we recognised the positive correlation between the expression of *ALKBH4* and *E2F1*, as well as between that of *ALKBH4* and of each of the E2F1-target genes, regardless of the histologic subtypes, using TCGA database (Supplementary Fig. S6A,B) in NSCLC. These results suggested that ALKBH4 upregulates the expression of *E2F1*, followed by that of its target genes, in NSCLC.

### High *ALKBH4* expression correlates with overall- and recurrence-free survival in NSCLC

**Figure 6:**
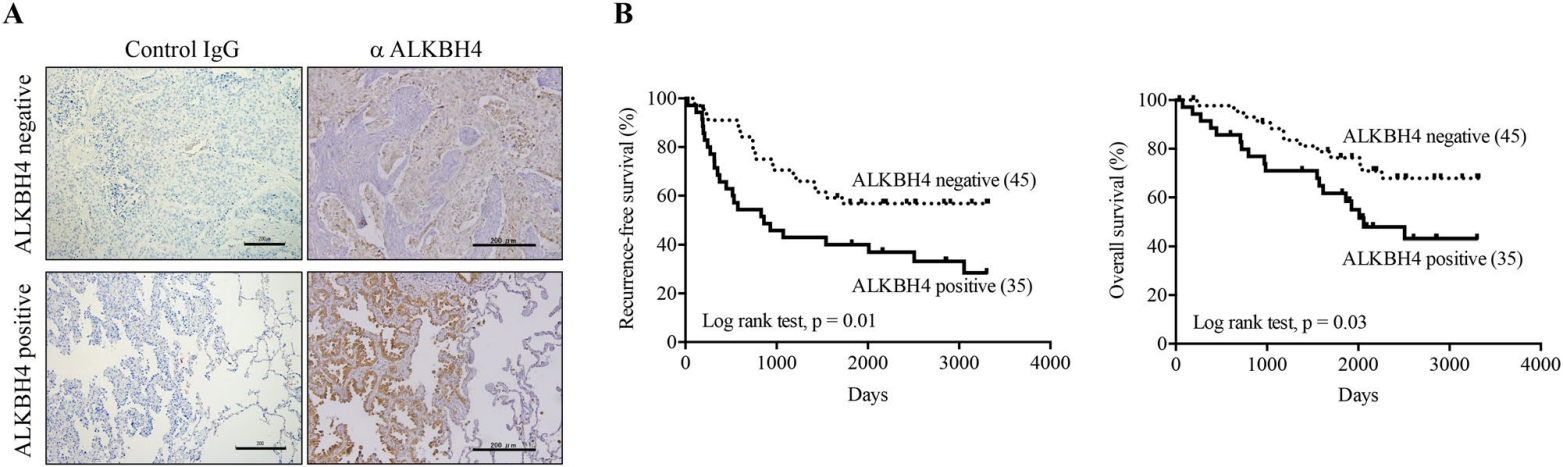
Association of ALKBH4 expression levels with recurrence-free survival and overall survival. (**A**) Expression of ALKBH4 in NSCLC specimens was examined using immunohistochemical staining. Representative results are shown. Black scale bars, 200 μm. Association of ALKBH4 expression levels with recurrence-free survival (**B**) and overall survival (**C**). NSCLC tissue specimens stained with anti-ALKBH4 were divided into two groups, according to ALKBH4 expression: negative (45 samples) and positive (35 samples). Data was statistically analysed using Log-rank test.

Finally, to clarify the relationship between *ALKBH4* expression and the prognosis of NSCLC patients, we performed immunohistochemical staining using anti-ALKBH4 antibody on NSCLC specimens. The immunohistochemical analysis showed that ALKBH4 was positive in 35 tumours and negative in 45 tumours (Fig. 6A). There was no significant intergroup heterogeneity regarding tumour size, presence or absence of pleural invasion, intrapulmonary metastasis, and nodal metastasis. During the follow up (median: 68.5 months; range: 2 to 110 months), 35 recurrences and 31 deaths occurred in a total of 80 patients. Kaplan–Meier survival curves are shown in Fig. 6B. The recurrence-free survival rate was significantly lower in the ALKBH4 positive group than in the ALKBH4 negative group (log rank test, p = 0.01). Likewise, the overall survival rate was significantly lower in the ALKBH4 positive group than in the ALKBH4 negative group (log rank test, p = 0.03). Additionally, we performed survival analysis using TCGA database. However, neither overall survival nor disease-free survival was significantly related to ALKBH4 mRNA expression levels in NSCLC (Supplementary Fig. S7).

## Discussion

In the present study, we demonstrated that *ALKBH4* is highly expressed in cell lines as well as in tumour tissues of NSCLC patients, and that it functions as a tumour promoter via its enzymatic activity. Knockdown of ALKBH4 downregulated the expression of *E2F1*, a critical regulator of the G_1_/S phase transition in NSCLC cells. *E2F1* expression was positively correlated with the expression of *ALKBH4* in NSCLC clinical samples. Moreover, the expression of E2F1-target genes (*CDC25A, CCNE1* and *MYBL2*) was positively correlated with the expression of *ALKBH4* in NSCLC clinical samples. *ALKBH4* knockdown downregulated the expression of *CDK2* and cyclin D3 encoding gene in A549 cells. Since *CDK2* and *CCND3* (cyclin D3) have also been reported as E2F1-target genes^32–35^, the downregulation of *CDK2* and *CCND3* might be due to the regulation of *E2F1* expression by ALKBH4. It is well known that *E2F1* is overexpressed in several cancers, including NSCLC^36–38^ and that an aberrant *E2F1* expression is correlated with a lower overall survival of NSCLC patients^26^. Taken together, the upregulated ALKBH4 expression plays a pivotal NSCLC-promoting role, leading to a poor prognosis in NSCLC patients.

Although we have shown that ALKBH4 overexpression promotes cell growth via its enzymatic activity (Fig. 2D,E), the effect of ALKBH4 enzymatic activity on downstream effector molecules such as E2F1 and E2F1-target genes remains unclear and requires further investigation.

Recently, it was reported that ALKBH4 functions as a demethylase for N6-adenosine modification in DNA^39^, and the *C. elegans* ALKBH4 ortholog (denoted NMAD-1) also has such a demethylase function^40^. Abrogation of ALKBH4 (or NMAD-1) function appears to have strong effects on various phenomena related to cell cycle progression, such as cytokinesis, DNA replication, and meiosis^15,41,42^. As shown in Supplementary Table S3, ALKBH4 knockdown also affected DNA replication and meiosis-related genes in A549 cells. Moreover, the enzymatic domain of ALKBH4 was critical for the upregulation of cell proliferation in NSCLC cells, suggesting that ALKBH4 may function as a tumour promoter by targeting N6-adenosine modification in NSCLC cells.

Although few publications suggest that gene expression is mostly regulated at the mRNA level^43,44^, many studies have reported a discrepancy between mRNA and protein levels^45,46^. Protein levels more closely reflect the cancer phenotype because these proteins execute major intracellular molecular functions. Therefore, we believe that survival analysis using IHC data reflects the true nature of ALKBH4 in NSCLC.

A high expression of *ALKBH4* is correlated with an overall- and recurrence-free survival in NSCLC. On the contrary, Supplementary Fig. S4 shows a positive correlation between the expression of *ALKBH4* and *E2F1*, as well as between that of *ALKBH4* and E2F1-target genes, which were only observed in early stage NSCLC tumour tissues, but not in those of late stage NSCLC. Among the Polo-like kinase (PLK) family, *PLK1* and *PLK4* are highly expressed in NSCLC^47,48^ and promote metastasis via epithelial–mesenchymal transition (EMT) induction^49,50^. Since *ALKBH4* knockdown downregulated the expression of *PLK1* and *PLK4* (Supplementary Table S2), ALKBH4 may function as a tumour promoter by accelerating cancer cell proliferation via upregulation of *E2F1* signalling in early stage, and by promoting metastasis via upregulation of PLK signalling in late-stage NSCLC.

ALKBH4 knockdown had no significant effect on the population of G_0_ phase cells in A549 cells. Although our gene array data showed that upstream regulators of E2F1 (Rb1 and cyclin D1) expression remained unchanged upon ALKBH4 knockdown, ubiquitin C-terminal hydrolase L5 (UCHL5, also known as UCH37, ubiquitin C-terminal hydrolase 37), which has been reported as a deubiquitinating enzyme of E2F1^51^, was downregulated upon ALKBH4 knockdown (Supplementary Table S2, Fold-change =  − 2.1). UCHL5 activates the E2F1 transcriptional activity by decreasing Lys-63-linked ubiquitination of E2F1. E2F1 can activate gene transcription of the E2F family (E2F1, E2F2, and E2F3), which in turn induces positive feedback on E2F1 gene transcription^52–55^. Moreover, *ALKBH4* knockdown downregulated the expression of *E2F2* (Supplementary Table S2). In addition, UCHL5 knockdown induces apoptosis in A549 cells^56^. Therefore, we believe that ALKBH4 knockdown-induced E2F1 reduction is due to the downregulation of UCHL5 expression in NSCLC cells.

We found that ALKBH4 functions as a tumour promoter in NSCLC. However, Shen et al. recently reported that *ALKBH4* expression is decreased in colon cancer tissues, compared to adjacent normal tissues, and functions as a tumour suppressor by decreasing H3K4me3 levels by competitively binding to methyltransferase WDR5^17^. WDR5 is overexpressed in colon cancer and the depletion of WDR5 reduces cell viability of colon cancer cell lines^57^. On the contrary, an overexpression of *WDR5* was reported to induce G_1_ arrest in A549 cells, independently of the H3K4me3 status, and WDR5 was suggested to possibly have different functions in different cancers^58^. Therefore, although the underlying mechanism should be analysed, the tumour promoting role of ALKBH4 in NSCLC might partly rely on WDR5.

Cancer cells adapt their metabolism to promote tumour growth. One important metabolic feature of cancer cells is the Warburg effect, which leads to high rates of glucose utilisation and lactate production^59^. It has been reported that E2F1 promotes this effect by enhancing glycolysis and repressing glucose oxidation in the mitochondria^60,61^. Moreover, E2F1-mediated repression of oxidative metabolism results in the self-renewal of cancer stem cells^62^, suggesting that ALKBH4 may confer the Warburg effect through an increased expression of *E2F1*, leading to efficient recurrence in NSCLC patients.

The specific types of mutations that confer drug sensitivity to EGFR-targeted drugs are present in the tyrosine kinase domain of the *EGFR* gene, corresponding to exons 19 and 21 with 5–20% of overall incidence^63^. In contrast, upregulated *ALKBH4* expression was independent of the mutation status of the *EGFR* gene in NSCLC specimens (Fig. 1G,H). Moreover, an increased *ALKBH4* expression was shown in broad types of cancer (Supplementary Fig. S3A). We showed that *ALKBH4* knockdown induced the downregulation of cell proliferation and G_1_ arrest in breast cancer cell line MCF-7 cells, as well as in NSCLC cells (Supplementary Fig. S3D,E, respectively). Therefore, we propose that ALKBH4 may be an important molecular target for, not only NSCLC, but also for a wide range of cancers.

## Materials and methods

### Clinical specimens

Specimens of NSCLC tissues and adjacent non-cancerous tissues were obtained from the patients who had undergone primary curative resection of a lung tumour at Kagoshima University Hospital (Japan) as described before^64^. All enrolled patients were diagnosed via pathological examination staged by specialised oncologists via the 7th edition of the International Association for the Study of Lung Cancer TNM Classification. Written informed consent was obtained from each of the patients and the study was approved by the Ethical Committee of Kagoshima University Hospital (registration No. 351). Experiments using clinical specimens were approved by the institutional review boards of the Graduate School of Pharmaceutical Sciences, Osaka University. All methods were carried out in accordance with relevant guidelines and regulations. Clinical and histopathological data related to the clinical specimens are presented in Supplementary Table S1.

### Antibodies

Monoclonal anti-ALKBH4 antibody (ab195379) was purchased from Abcam (Cambridge, UK) and used for Western blotting. Polyclonal anti-ALKBH4 antibody (NBP2-14737) was purchased from Novus Biologicals (Littleton, Colorado, USA) and used for IHC and ICC experiments. Monoclonal anti-CDK2 antibody was purchased from BD Transduction Laboratories (San Jose, CA, USA). Monoclonal anti-β-actin antibody was purchased from Sigma (St. Louis, Missouri, United States). Monoclonal anti-CDK4 and anti-cyclin D3 antibodies and anti-rabbit IgG, HRP-linked antibody (#7074) and anti-mouse IgG, HRP-linked antibody (#7076) were purchased from Cell signalling technology (Danvers, MA 01923, USA).

### Cell culture

Twenty-three human lung cancer cell lines (Calu-3, NCI-H2228, NCI-H1792, NCI-H1437, NCI-H1650, NCI-H1755, NCI-H1838, RERF-LC-KJ, NCI-H23, NCI-H1975, PC-9, LC-2/ad, HLC-1, A549, II-18, RERF-LC-AI, NCI-H226, Sq-1, NCI-H520, LK-2, EBC-1, and LC-1F cells) obtained from the American Type Culture Collection (ATCC), were cultured in RPMI1640 medium (Wako) supplemented with 10% foetal bovine serum, 100 U/mL penicillin G, and 0.1 μg/mL streptomycin.

### Western blotting

Western blotting was conducted as described before^65^. Protein samples were separated on a 7.5–15% sodium dodecyl sulphate (SDS)-polyacrylamide gel electrophoresis (PAGE) gel, and then transferred to a polyvinylidene difluoride (PVDF) membrane by using the Bio-Rad semidry transfer system (1 h, 12 V) (Hercules, CA, USA). Immunoreactive proteins were visualised by treatment with a detection reagent (ECL Prime western blotting detection reagent, GE Healthcare), using the antibodies described above, in an ImageQuant LAS 4000 mini system (GE Healthcare). Densitometric analysis was performed using the National Institute of Health (NIH) Image J software.

### Construction of the ALKBH4 expression vector

PCR-amplified wild-type *ALKBH4* was cloned into pEB Multi-Neo vector (Wako). The primer sequences used for the gene amplification were as follows: forward primer, 5′-GAATTCGCGGAAATGGCTGGGAGGGG-3′ and reverse primer, 5′-GGGACATAGTAGATTACAGGTGG-3′. Inserted *ALKBH4* sequence was confirmed via sequencing. Catalytic domain-mutated ALKBH4 (H169A/D171A) was purchased from GenScript (Piscataway, NJ, USA).

### Cell cycle analysis

A549 or II-18 cells were transfected with the ALKBH4 siRNA or control siRNA. NCI-H23 cells were transfected with the pEB Multi-Neo-ALKBH4 vector (wild-type or mutant). After incubation for 48 h, cells were fixed with 70% ethanol and were washed with phosphate buffered saline (PBS). Cells were suspended in a hypotonic solution containing 5 mg/mL propidium iodide (PI). PI-stained samples (1 × 10^5^ cells) were analysed for fluorescence using an FACS Calibur (Becton–Dickinson, Franklin Lakes, New Jersey, USA).

### Apoptosis analysis

A549 cells transfected with ALKBH4 or control siRNAs were cultured for 48 h and stained with ethidium homodimer III and fluorescein isothiocyanate-conjugated Annexin V (Apoptotic & Necrotic Cell Detection Kits, TaKaRa) according to the manufacturer's protocol. Flow cytometric analysis was conducted using MACSQuant X (MiltenyiBiotec, BergischGladbach, Germany).

### G_0_-marker analysis

A549 cells transfected with ALKBH4 or control siRNAs were cultured for 72 h, and G_0_-marker assay was conducted using senescence-associated β-galactosidase (Senescence Detection Kit, BioVision, Milpitas, CA, USA) according to the manufacturer’s protocol. β-galactosidase-positive cells were counted in randomly selected 25 fields.

### Cell proliferation assay

Cell proliferation was examined using the xCELLigenceReal Time Cell Analyzer Dual Purpose (RTCA DP) system (Roche, Basel, Switzerland). A549 or II-18 cells were transfected with the ALKBH4 siRNA or control siRNA. After 24 h of incubation, cells were reseeded on an E-plate 16 (A549 cells: 1 × 10^3^ cells/well; II-18 cells: 3 × 10^3^ cells/well) and incubated for the indicated times. Water-soluble tetrazolium salt-8 (WST-8) reagent (Dojindo) was used for the cell proliferation assay (Fig. 2D), which was conducted as described previously^66^. NCI-H23 cells transfected with the pEB Multi-Neo-ALKBH4 vector (wild-type or mutant) and ALKBH4 siRNAs were reseeded in a 96-well plate and incubated for the indicated times. After incubation for 2 h with WST-8 reagent at 37 °C and 5% CO_2_, the optical density was determined at 450/630 nm (Ex/Em).

### siRNA and DNA transfection

siRNA duplexes were used to downregulate ALKBH4 mRNA (ALKBH4 stealth siRNA #1: ACAUACCGUUUCAUUUACUGCUCCG; ALKBH4 stealth siRNA #2: ACAGAGGAGUCUGACUUUGAGGGCU) and stealth RNAi siRNA negative control low GC were purchased from Life technologies (Carlsbad, CA, USA). For all siRNA transfection studies, A549 cells (4 × 10^4^ cells/well) or II-18 (5 × 10^4^ cells/well) were transfected on a 12-well plate using Lipofectamine RNAiMAX (Life Technologies). For DNA transfection, A549 cells (4 × 10^4^ cells/well) seeded on a 24-well plate were transfected with pEB Multi-Neo vector or pEB Multi-Neo-ALKBH4 vector using Lipofectamine 3000 and P3000 reagent (Life Technologies).

### Co-transfection of siRNA and expression vector

NCI-H23 cells (15 × 10^4^ cells/well) seeded on a 12-well plate were transfected with pEB Multi-Neo-ALKBH4 vector (wild-type or mutant) and ALKBH4 siRNAs using Lipofectamine 2000 (Life Technologies).

### Quantitative PCR (qPCR)

Total RNA was isolated by using RNeasy Plus Mini Kit (QIAGEN, Hilden, Germany). Prime Script RT reagent Kit (Takara, Mountain View, USA) was used to prepare cDNA from 500 ng of total RNA. The Light cycler 96 plate (Roche) was used for qPCR analysis. The thermal cycling conditions used were as follows: an initial step at 95 °C for 10 s and 40 cycles of 95 °C for 5 s, and 60 °C for 20 s for *ALKBH4*; an initial step at 95 °C for 10 s and 40 cycles of 95 °C for 5 s, and 60 °C for 20 s for β-actin gene (*ACTB*); an initial step at 95 °C for 30 s and 50 cycles of 95 °C for 5 s, 60 °C for 10 s, and 68 °C for 15 s for *E2F1* and *CDC25A*; and an initial step at 95 °C for 30 s and 50 cycles of 95 °C for 5 s, 58 °C for 10 s and 68 °C for 15 s for *CCNE1* and *MYBL2*. The primer sequences used for gene amplification were as follows: *ALKBH4* forward, 5′-TGATGCTGATCGAGGACTTTGTG-3′, and reverse, 5′-AAGCCCTCGGTCTTTAGCTTCTG-3′; *ACTB* forward, 5′-TGGCACCCAGCACAATGAA-3′, and reverse, 5′-CTAAGTCATAGTCCGCCTAGAAGCA-3′; *E2F1* forward, 5′-CAAGAAGTCCAAGAACCACATCC-3′, and reverse, 5′-AGATATTCATCAGGTGGTCCAGC-3′; *CDC25A* forward, 5′-TTGTTGTGTTTCACTGCGAGTTTT-3′, and reverse, 5′-AGGGTAGTGGAGTTTGGGGTATTC-3′; *CCNE1* forward, 5′-GCCAGCCTTGGGACAATAATG-3′, and reverse, 5′-CTTGCACGTTGAGTTTGGGT-3′; *MYBL2* forward, 5′-CATTGTGGATGAGGATGTGAAGC-3′, and reverse, 5′-TGGTTGAGCAAGCTGTTGTCTTC-3′.

### Gene array analysis

Total RNA was obtained from the *ALKBH4* knockdown cells and from cells with an overexpression of *ALKBH4* by using miRNeasy Mini Kit (QIAGEN). Total RNA (100 ng) was converted to cDNA and biotinylated using Ambion WT Expression Kit (Thermo Fisher Scientific, Waltham, Massachusetts, United States). Subsequently, biotinylated cDNA was hybridised to the GeneChip Human 2.0 ST Array (Affymetrix, Santa Clara, California, United States) and scanned using GeneChip Scanner 3000 (Affymetrix). The obtained gene array data were analysed using Partek Genomic Suite 6.6 software. Enrichment analysis was conducted by using Enricher (https://amp.pharm.mssm.edu/Enrichr/)^67^.

### Establishment of ALKBH4 shRNA stable cells

A549 cells were seeded on the day before lentivirus infection and cultured in DMEM (Wako) supplemented with 10% foetal bovine serum and 100 mg/L kanamycin at 37 °C under a 5% CO_2_ atmosphere. Lentiviral particles, which were purchased from Sigma Aldrich (control shRNA: SHC002V and ALKBH4 shRNA: SHCLNV-NM_17621), were added to the culture medium to 4 multiplicity of infection (MOI), and polybrene (Thermo Fisher Scientific) was added at a final concentration of 8 ng/µL. After selection by 5 µg/mL puromycin (Sigma Aldrich), the expression levels of *ALKBH4* were confirmed using qPCR, and cell lines with a high *ALKBH4* knockdown efficiency were used for the next experiments.

### Establishment of ALKBH4 shRNA stable cell-xenografted mice

Female BALB/c nude mice were obtained from Oriental Yeast Corporation (Tokyo, JAPAN). Five-week-old mice were used for ALKBH4 shRNA stable cell-xenograft experiments. Animals were kept under 12 h light–dark cycles at 22–24 °C. A549 cells, which had been stably transfected with ALKBH4 shRNA (A549-shALKBH4) and control shRNA (A549-shControl), were both adjusted to a concentration of 0.6 × 10^7^ cells in 100 μL of serum-free DMEM. The cell suspensions, together with 100 μL of Matrigel Matrix High Concentration (Corning, New York, USA) were then injected subcutaneously into the right flanks of BALB/c nude mice (A549-shALKBH4, n = 8; A549-shControl, n = 8). One xenografted mouse, which was inoculated with A549-shALKBH4, died during the experiment. The tumour volume was calculated as follows: (tumour length × tumour width^2^)/2. All procedures were performed under a protocol approved by the Animal Experimentation Committee at Osaka University. All methods were carried out in accordance with relevant ARRIVE guidelines and regulations. We confirmed that all methods were carried out in accordance with relevant guidelines and regulations. Developed tumours were resected 52 days after xenografts.

### ALKBH4 immunohistochemistry in clinical cases

Eighty patients who underwent radical operation for primary lung adenocarcinoma at Kagoshima University Hospital from January 2001 to December 2007 were subjected to immunohistochemistry for ALKBH4. Immunohistochemistry was conducted as described before^64^. Paraffin-embedded section (3 μm of thickness) were deparaffinised and dehydrated. The endogenous peroxidase activity of specimens was blocked using a 0.3% hydrogen peroxide solution in methanol. The sections were blocked with 1% bovine serum albumin and were incubated with the rabbit polyclonal antibody against human ALKBH4 (1:200; Novus Biologicals, NBP2-14737) overnight at 4 °C, followed by staining with a streptavidin–biotin peroxidase kit (Vector Laboratories, CA, USA). The immune complex was visualised by incubating the sections with diaminobenzidine tetrahydrochloride. The sections were counterstained with haematoxylin and mounted. Non-cancerous colon samples were used as positive controls for ALKBH4. ALKBH4 expression was determined by counting the number of cancer cells in which the cytoplasm was stained with the anti-ALKBH4 antibody. Two investigators evaluated ALKBH4 expression via immunohistochemistry within each tumour by assessing a total of 1000 cancer cells in 10 selected fields (100 cells/field) using high-power (× 200) microscopy, in an independent manner. The average labelling index of ALKBH4 was assessed according to the proportion of positive cells present in each field. Tumours with an average labelling index of 20% or more were defined as ALKBH4-positive. The specificity of the anti-ALKBH4 antibody was verified through immunofluorescence staining using A549 cells transfected with shControl and shALKBH4 (Supplementary Fig. S8).

### Statistics

The results were expressed as the mean ± standard deviation (S.D.). Differences between the values were statistically analysed using the Student’s *t*-test, paired *t*-test or one-way analysis of variance (ANOVA) with Bonferroni post-hoc tests (GraphPad Prism 6.0, GraphPad software). Pearson correlation analysis was used for the correlation analysis. A *p*-value < 0.05 was considered statistically significant.

### Ethical approval

The animal experiments were approved by the Animal Experimentation Committee at Osaka University. All animal experiments were performed in accordance with relevant guideline and regulations. For human research, written informed consent was obtained from each of the patients and the study was approved by the Ethical Committee of Kagoshima University Hospital (registration No. 351). Experiments using clinical specimens were approved by the institutional review boards of the Graduate School of Pharmaceutical Sciences, Osaka University.

## Supplementary Information

Supplementary Information.
