# Supplementary material for: ALKBH4 promotes tumourigenesis with a poor prognosis in non-small-cell lung cancer
Source: Sci Rep. 2021 Apr 21;11:8677. doi: 10.1038/s41598-021-87763-1 (PMC8060266; doi:10.1038/s41598-021-87763-1)
Supplement: Supplementary file 1 — Supplementary Information. [file 41598_2021_87763_MOESM1_ESM.pdf]

ALKBH4 promotes tumourigenesis with a poor prognosis in non-small-cell lung cancer

Kentaro Jingushi[1*], Masaya Aoki[2*], Kazuhiro Ueda[2], Takahiro Kogaki[1], Masaya Tanimoto[1], Yuya Monoe[1], Masayuki Ando[1], Takuya Matsumoto[1], Kentaro Minami[3], Yuko Ueda[1], Kaori Kitae[1], Hiroaki Hase[1], Toshiyuki Nagata[2], Aya Harada Takeda[2], Masatatsu Yamamoto[3], Kohichi Kawahara[3], Kazuhiro Tabata[4], Tatsuhiko Furukawa[3], Masami Sato[2], and Kazutake Tsujikawa[1]

[1]Laboratory of Molecular and Cellular Physiology, Graduate School of Pharmaceutical Sciences, Osaka University, 1-6 Yamadaoka, Suita, Osaka 565-0871, Japan

[2]Department of General Thoracic Surgery, Graduate School of Medical and Dental Sciences, Kagoshima University, 8-35-1 Sakuragaoka, Kagoshima, Kagoshima 890-8520, Japan

[3]Department of Molecular Oncology, Graduate School of Medical and Dental Sciences, Kagoshima University, 8-35-1 Sakuragaoka, Kagoshima, Kagoshima 890-8544, Japan

[4]Human Pathology, Kagoshima University Graduate School of Medical and Dental Sciences, 8-35-1 Sakuragaoka, Kagoshima City, 890-8544, Japan

Supplementary Information

Supplementary tables

Supplementary table 1. Clinical and histopathological information.

Clinical information of matched-pair NSCLC samples used in Fig. 1

**Histological type**		**Stage classification**	
Adenocarcinoma	50	IA	34
Adenocarcinoma with mixed subtypes	16	IB	26
Squamous cell carcinoma	23	IIA	9
**Age (year)**		IIB	4
Mean	70	IIIA	16
Range	44-88	**TNM classification**	
**Gender**		**Tumour (T)**	
Male	55	T1	41
Female	34	T2	43
***EGFR* gene status (adenocarcinoma)**		T3	4
Wild-type	26	T4	1

Mutant (exon 18, 19, 21)	21	**Node (N)**	
unknown	3	N0	64
		N1	8
		N2	15
		Nx	2
		**Metastasis (M)**	
		M0	89

Clinical information of matched-pair NSCLC samples used in Fig. 5 and SFig. 4

**Histological type**		**Stage classification**	
Adenocarcinoma	37	IA	18
Squamous cell carcinoma	5	IB	11
**Age (year)**		IIA	3
Mean	70	IIB	3
Range	44-87	IIIA	7
**Gender**		**TNM classification**	
Male	25	**T (Tumour)**	
Female	17	T1	22
***EGFR* gene status (adenocarcinoma)**		T2	17

Wild-type	22	T3	2
Mutant (exon 18, 19, 21)	17	T4	1
unknown	3	**N (Node)**	
		N0	31
		N1	5
		N2	6
		**M (Metastasis)**	
		M0	42

Characteristics according to presence or absence of ALKBH4 immunoreactivity in Fig. 6

**Variables**		**Total (n=80)**	**ALKBH4**		**P**
			**Positive (n=35)**	**Negative (n=45)**	
**Age**	Years	69.2 +/- 8.2	71.1 +/- 7.4	67.7 +/- 8.6	0.069
**Sex**	Male / Female	44 / 36	18 / 17	26 / 19	0.653
**Smoking**	Yes / No	34 / 46	16 / 19	18 / 27	0.653
**Tumour size**	mm	36.2 +/- 15.7	37.8 +/- 17.3	34.9 +/- 14.4	0.420
**PL**	+ / -	32 / 48	14 / 21	18 / 27	1.000
**PM**	+ / -	9 / 71	5 / 30	4 / 41	0.745

**T (Tumour) descriptor**	T1 / >T2	25 / 55	11 / 24	14 / 31	1.000
**N (Node) descriptor**	N0 / >N1	20 / 60	10 / 25	10 / 35	0.606

PL; pleural invasion, PM; intrapulmonary metastasis

Supplementary table 2. Genes affected by ALKBH4 knockdown (FC>|1.5|)

Gene Symbol	Fold-Change (ALKBH4 KD vs. Control)	(Description)
APOLD1	-16.6038	siABH4#1 5nM down vs siC 5nM
HIST1H1B	-16.492	siABH4#1 5nM down vs siC 5nM
PLK4	-16.4602	siABH4#1 5nM down vs siC 5nM
NCAPG	-16.0112	siABH4#1 5nM down vs siC 5nM
KIF14	-15.8056	siABH4#1 5nM down vs siC 5nM
KIF11	-15.7553	siABH4#1 5nM down vs siC 5nM
NDC80	-15.2768	siABH4#1 5nM down vs siC 5nM
TTK	-15.0455	siABH4#1 5nM down vs siC 5nM
PBK	-14.922	siABH4#1 5nM down vs siC 5nM
MKI67	-14.896	siABH4#1 5nM down vs siC 5nM
TICRR	-14.7299	siABH4#1 5nM down vs siC 5nM
ASPM	-14.5225	siABH4#1 5nM down vs siC 5nM
FAM111B	-13.9955	siABH4#1 5nM down vs siC 5nM
CCNA2	-13.9424	siABH4#1 5nM down vs siC 5nM
CKAP2L	-13.9418	siABH4#1 5nM down vs siC 5nM
GINS2	-13.7191	siABH4#1 5nM down vs siC 5nM
SHCBP1	-13.3965	siABH4#1 5nM down vs siC 5nM
CENPE	-13.3735	siABH4#1 5nM down vs siC 5nM
DLGAP5	-13.2239	siABH4#1 5nM down vs siC 5nM

DTL	-12.8806	siABH4#1 5nM down vs siC 5nM
KIF20A	-12.6702	siABH4#1 5nM down vs siC 5nM
MCM10	-12.5643	siABH4#1 5nM down vs siC 5nM
BUB1	-12.3764	siABH4#1 5nM down vs siC 5nM
HMMR	-12.1054	siABH4#1 5nM down vs siC 5nM
NUF2	-11.5648	siABH4#1 5nM down vs siC 5nM
CCNB2	-11.5638	siABH4#1 5nM down vs siC 5nM
CDCA2	-11.2596	siABH4#1 5nM down vs siC 5nM
ESCO2	-10.9047	siABH4#1 5nM down vs siC 5nM
KIF20B	-10.8747	siABH4#1 5nM down vs siC 5nM
AURKB	-10.754	siABH4#1 5nM down vs siC 5nM
MND1	-10.6562	siABH4#1 5nM down vs siC 5nM
MAD2L1	-10.4999	siABH4#1 5nM down vs siC 5nM
CEP55	-10.4921	siABH4#1 5nM down vs siC 5nM
SGOL1	-10.4698	siABH4#1 5nM down vs siC 5nM
PLK1	-10.2261	siABH4#1 5nM down vs siC 5nM
DSCC1	-10.1619	siABH4#1 5nM down vs siC 5nM
NEK2	-10.0751	siABH4#1 5nM down vs siC 5nM
SKA1	-10.053	siABH4#1 5nM down vs siC 5nM
EXO1	-9.92716	siABH4#1 5nM down vs siC 5nM
FAM72D	-9.88441	siABH4#1 5nM down vs siC 5nM
ANLN	-9.74445	siABH4#1 5nM down vs siC 5nM
FAM64A	-9.66299	siABH4#1 5nM down vs siC 5nM
TOP2A	-9.57711	siABH4#1 5nM down vs siC 5nM
ATAD2	-9.53211	siABH4#1 5nM down vs siC 5nM
SPC24	-9.47497	siABH4#1 5nM down vs siC 5nM
BRCA2	-9.36104	siABH4#1 5nM down vs siC 5nM
SKA3	-9.18649	siABH4#1 5nM down vs siC 5nM
PRR11	-9.02498	siABH4#1 5nM down vs siC 5nM
RRM2	-9.02179	siABH4#1 5nM down vs siC 5nM
PRC1	-8.87233	siABH4#1 5nM down vs siC 5nM
CENPF	-8.86193	siABH4#1 5nM down vs siC 5nM

NCAPH	-8.60178	siABH4#1 5nM down vs siC 5nM
CCNB1	-8.39253	siABH4#1 5nM down vs siC 5nM
CENPI	-8.38065	siABH4#1 5nM down vs siC 5nM
KIAA0101	-8.3741	siABH4#1 5nM down vs siC 5nM
CDC20	-8.37284	siABH4#1 5nM down vs siC 5nM
ARHGAP11A	-8.32363	siABH4#1 5nM down vs siC 5nM
RFC3	-8.31795	siABH4#1 5nM down vs siC 5nM
CASC5	-8.21945	siABH4#1 5nM down vs siC 5nM
CDK1	-8.12241	siABH4#1 5nM down vs siC 5nM
HIST1H2BB	-8.11403	siABH4#1 5nM down vs siC 5nM
CDCA8	-8.02194	siABH4#1 5nM down vs siC 5nM
DEPDC1B	-8.01651	siABH4#1 5nM down vs siC 5nM
RAD51AP1	-7.72857	siABH4#1 5nM down vs siC 5nM
CENPK	-7.60228	siABH4#1 5nM down vs siC 5nM
KIF15	-7.56282	siABH4#1 5nM down vs siC 5nM
HELLS	-7.56267	siABH4#1 5nM down vs siC 5nM
POLQ	-7.49298	siABH4#1 5nM down vs siC 5nM
FANCD2	-7.40383	siABH4#1 5nM down vs siC 5nM
MYBL2	-7.39086	siABH4#1 5nM down vs siC 5nM
GSG2	-7.35282	siABH4#1 5nM down vs siC 5nM
CENPH	-7.33772	siABH4#1 5nM down vs siC 5nM
KIF2C	-7.26555	siABH4#1 5nM down vs siC 5nM
CDC6	-7.17895	siABH4#1 5nM down vs siC 5nM
KIAA1524	-7.08003	siABH4#1 5nM down vs siC 5nM
ARHGAP11B	-7.02902	siABH4#1 5nM down vs siC 5nM
FOXM1	-7.02794	siABH4#1 5nM down vs siC 5nM
C5orf34	-6.97375	siABH4#1 5nM down vs siC 5nM
KIF18A	-6.94117	siABH4#1 5nM down vs siC 5nM
OIP5	-6.90794	siABH4#1 5nM down vs siC 5nM
BIRC5	-6.84899	siABH4#1 5nM down vs siC 5nM
E2F8	-6.83216	siABH4#1 5nM down vs siC 5nM
FANCI	-6.81707	siABH4#1 5nM down vs siC 5nM

HIST1H2AB	-6.81241	siABH4#1 5nM down vs siC 5nM
TPX2	-6.72578	siABH4#1 5nM down vs siC 5nM
BLM	-6.71038	siABH4#1 5nM down vs siC 5nM
NUSAP1	-6.69165	siABH4#1 5nM down vs siC 5nM
KIF4B	-6.59987	siABH4#1 5nM down vs siC 5nM
ESPL1	-6.59585	siABH4#1 5nM down vs siC 5nM
WDHD1	-6.57117	siABH4#1 5nM down vs siC 5nM
HIST1H2AM	-6.31514	siABH4#1 5nM down vs siC 5nM
MELK	-6.301	siABH4#1 5nM down vs siC 5nM
DEPDC1	-6.28611	siABH4#1 5nM down vs siC 5nM
DBF4	-6.23555	siABH4#1 5nM down vs siC 5nM
ZWILCH	-6.21751	siABH4#1 5nM down vs siC 5nM
STIL	-6.18652	siABH4#1 5nM down vs siC 5nM
ATAD5	-6.15193	siABH4#1 5nM down vs siC 5nM
KNTC1	-6.13994	siABH4#1 5nM down vs siC 5nM
GTSE1	-6.13855	siABH4#1 5nM down vs siC 5nM
MNS1	-6.10306	siABH4#1 5nM down vs siC 5nM
HIST1H2AI	-6.08972	siABH4#1 5nM down vs siC 5nM
XRCC2	-6.0895	siABH4#1 5nM down vs siC 5nM
POLE2	-5.93548	siABH4#1 5nM down vs siC 5nM
HIST1H3J	-5.90828	siABH4#1 5nM down vs siC 5nM
MCM6	-5.87355	siABH4#1 5nM down vs siC 5nM
CENPU	-5.85924	siABH4#1 5nM down vs siC 5nM
ORC6	-5.82719	siABH4#1 5nM down vs siC 5nM
KIF18B	-5.81819	siABH4#1 5nM down vs siC 5nM
HIST1H2BM	-5.76136	siABH4#1 5nM down vs siC 5nM
NCAPG2	-5.74406	siABH4#1 5nM down vs siC 5nM
E2F1	-5.65427	siABH4#1 5nM down vs siC 5nM
IFI30	-5.62471	siABH4#1 5nM down vs siC 5nM
FAM83D	-5.62223	siABH4#1 5nM down vs siC 5nM
SPAG5	-5.58672	siABH4#1 5nM down vs siC 5nM
SGOL2	-5.55609	siABH4#1 5nM down vs siC 5nM

PARPBP	-5.52789	siABH4#1 5nM down vs siC 5nM
MCM7	-5.4882	siABH4#1 5nM down vs siC 5nM
PRIM1	-5.46362	siABH4#1 5nM down vs siC 5nM
SMC2	-5.43353	siABH4#1 5nM down vs siC 5nM
BORA	-5.43208	siABH4#1 5nM down vs siC 5nM
CIT	-5.43057	siABH4#1 5nM down vs siC 5nM
LMNB1	-5.3732	siABH4#1 5nM down vs siC 5nM
MCM4	-5.36643	siABH4#1 5nM down vs siC 5nM
MASTL	-5.32236	siABH4#1 5nM down vs siC 5nM
RBL1	-5.32118	siABH4#1 5nM down vs siC 5nM
DIAPH3	-5.3179	siABH4#1 5nM down vs siC 5nM
ASF1B	-5.3107	siABH4#1 5nM down vs siC 5nM
NEIL3	-5.30784	siABH4#1 5nM down vs siC 5nM
KIF4A	-5.28081	siABH4#1 5nM down vs siC 5nM
TRIP13	-5.27463	siABH4#1 5nM down vs siC 5nM
WDR76	-5.2711	siABH4#1 5nM down vs siC 5nM
KIF22	-5.24802	siABH4#1 5nM down vs siC 5nM
SMC4	-5.23923	siABH4#1 5nM down vs siC 5nM
UBE2C	-5.22773	siABH4#1 5nM down vs siC 5nM
CDC25A	-5.20816	siABH4#1 5nM down vs siC 5nM
RPS28	-5.19491	siABH4#1 5nM down vs siC 5nM
HIST1H2BE	-5.11654	siABH4#1 5nM down vs siC 5nM
TK1	-5.10068	siABH4#1 5nM down vs siC 5nM
HIST1H2AK	-5.08953	siABH4#1 5nM down vs siC 5nM
CDKN3	-5.07022	siABH4#1 5nM down vs siC 5nM
HIST1H2BL	-5.05341	siABH4#1 5nM down vs siC 5nM
CLSPN	-4.99926	siABH4#1 5nM down vs siC 5nM
BRCA1	-4.99075	siABH4#1 5nM down vs siC 5nM
SPC25	-4.96752	siABH4#1 5nM down vs siC 5nM
SLC35A4	-4.95311	siABH4#1 5nM down vs siC 5nM
RAD51	-4.87188	siABH4#1 5nM down vs siC 5nM
CENPN	-4.87163	siABH4#1 5nM down vs siC 5nM

AURKA	-4.85495	siABH4#1 5nM down vs siC 5nM
GAS2L3	-4.84213	siABH4#1 5nM down vs siC 5nM
FANCB	-4.80545	siABH4#1 5nM down vs siC 5nM
MCM8	-4.77755	siABH4#1 5nM down vs siC 5nM
HIST2H3A	-4.77456	siABH4#1 5nM down vs siC 5nM
HIST2H3A	-4.77456	siABH4#1 5nM down vs siC 5nM
CCDC34	-4.76496	siABH4#1 5nM down vs siC 5nM
HJURP	-4.75546	siABH4#1 5nM down vs siC 5nM
C1orf112	-4.64941	siABH4#1 5nM down vs siC 5nM
MCM5	-4.64867	siABH4#1 5nM down vs siC 5nM
KIF23	-4.57384	siABH4#1 5nM down vs siC 5nM
SLF1	-4.53451	siABH4#1 5nM down vs siC 5nM
SUV39H1	-4.50538	siABH4#1 5nM down vs siC 5nM
IQGAP3	-4.47391	siABH4#1 5nM down vs siC 5nM
SYT11	-4.47321	siABH4#1 5nM down vs siC 5nM
MIR924HG	-4.43035	siABH4#1 5nM down vs siC 5nM
UBE2T	-4.4206	siABH4#1 5nM down vs siC 5nM
FANCM	-4.40303	siABH4#1 5nM down vs siC 5nM
HIST1H4B	-4.39541	siABH4#1 5nM down vs siC 5nM
CDC45	-4.39064	siABH4#1 5nM down vs siC 5nM
MIR891A	-4.37523	siABH4#1 5nM down vs siC 5nM
CDCA3	-4.36528	siABH4#1 5nM down vs siC 5nM
MIS18BP1	-4.35988	siABH4#1 5nM down vs siC 5nM
BRIP1	-4.35476	siABH4#1 5nM down vs siC 5nM
FBXO5	-4.33098	siABH4#1 5nM down vs siC 5nM
RAD54L	-4.30687	siABH4#1 5nM down vs siC 5nM
FANCA	-4.26537	siABH4#1 5nM down vs siC 5nM
KNSTRN	-4.2132	siABH4#1 5nM down vs siC 5nM
NEMP1	-4.20239	siABH4#1 5nM down vs siC 5nM
C18orf54	-4.19493	siABH4#1 5nM down vs siC 5nM
NCAPD2	-4.19304	siABH4#1 5nM down vs siC 5nM
HIST1H2AJ	-4.19056	siABH4#1 5nM down vs siC 5nM

PRTFDC1	-4.16527	siABH4#1 5nM down vs siC 5nM
POLA2	-4.14265	siABH4#1 5nM down vs siC 5nM
MCM2	-4.1169	siABH4#1 5nM down vs siC 5nM
VRK1	-4.10896	siABH4#1 5nM down vs siC 5nM
CHAF1B	-4.08779	siABH4#1 5nM down vs siC 5nM
CEP128	-4.08001	siABH4#1 5nM down vs siC 5nM
MTBP	-4.06215	siABH4#1 5nM down vs siC 5nM
HIST1H4D	-4.06038	siABH4#1 5nM down vs siC 5nM
HIST1H3B	-4.03407	siABH4#1 5nM down vs siC 5nM
PSMC3IP	-4.01797	siABH4#1 5nM down vs siC 5nM
FIGNL1	-3.95905	siABH4#1 5nM down vs siC 5nM
POLA1	-3.94853	siABH4#1 5nM down vs siC 5nM
LRR1	-3.92456	siABH4#1 5nM down vs siC 5nM
CENPW	-3.89243	siABH4#1 5nM down vs siC 5nM
MCM3	-3.8761	siABH4#1 5nM down vs siC 5nM
MIR4712	-3.87428	siABH4#1 5nM down vs siC 5nM
SASS6	-3.86981	siABH4#1 5nM down vs siC 5nM
SLC16A6	-3.86598	siABH4#1 5nM down vs siC 5nM
ZNF724P	-3.86299	siABH4#1 5nM down vs siC 5nM
RAB39A	-3.81617	siABH4#1 5nM down vs siC 5nM
RIBC2	-3.81437	siABH4#1 5nM down vs siC 5nM
CDC25C	-3.80585	siABH4#1 5nM down vs siC 5nM
ORC1	-3.80224	siABH4#1 5nM down vs siC 5nM
KIF24	-3.77962	siABH4#1 5nM down vs siC 5nM
EME1	-3.76465	siABH4#1 5nM down vs siC 5nM
PTTG1	-3.76384	siABH4#1 5nM down vs siC 5nM
HIST1H4A	-3.75718	siABH4#1 5nM down vs siC 5nM
UNG	-3.75083	siABH4#1 5nM down vs siC 5nM
DSN1	-3.73656	siABH4#1 5nM down vs siC 5nM
CEP152	-3.73005	siABH4#1 5nM down vs siC 5nM
HIST1H3I	-3.72719	siABH4#1 5nM down vs siC 5nM
DDIAS	-3.72139	siABH4#1 5nM down vs siC 5nM

CENPQ	-3.70559	siABH4#1 5nM down vs siC 5nM
MUC5AC	-3.70082	siABH4#1 5nM down vs siC 5nM
CENPJ	-3.66567	siABH4#1 5nM down vs siC 5nM
SHMT1	-3.657	siABH4#1 5nM down vs siC 5nM
WDR62	-3.65209	siABH4#1 5nM down vs siC 5nM
CENPP	-3.63928	siABH4#1 5nM down vs siC 5nM
CDCA5	-3.63728	siABH4#1 5nM down vs siC 5nM
PAK6	-3.63046	siABH4#1 5nM down vs siC 5nM
ANP32E	-3.62537	siABH4#1 5nM down vs siC 5nM
GINS3	-3.60597	siABH4#1 5nM down vs siC 5nM
TMPO	-3.59943	siABH4#1 5nM down vs siC 5nM
MIR4668	-3.59208	siABH4#1 5nM down vs siC 5nM
NCAPD3	-3.58621	siABH4#1 5nM down vs siC 5nM
ND6	-3.56063	siABH4#1 5nM down vs siC 5nM
CCNF	-3.55301	siABH4#1 5nM down vs siC 5nM
RTKN2	-3.54821	siABH4#1 5nM down vs siC 5nM
RRM1	-3.54669	siABH4#1 5nM down vs siC 5nM
ERCC6L	-3.54171	siABH4#1 5nM down vs siC 5nM
LOC399815	-3.54093	siABH4#1 5nM down vs siC 5nM
MMS22L	-3.53569	siABH4#1 5nM down vs siC 5nM
USP1	-3.53314	siABH4#1 5nM down vs siC 5nM
ASB4	-3.52452	siABH4#1 5nM down vs siC 5nM
HIST1H4L	-3.51949	siABH4#1 5nM down vs siC 5nM
HAUS8	-3.5171	siABH4#1 5nM down vs siC 5nM
DBF4B	-3.49138	siABH4#1 5nM down vs siC 5nM
POLE	-3.46654	siABH4#1 5nM down vs siC 5nM
INCENP	-3.44305	siABH4#1 5nM down vs siC 5nM
MTFR2	-3.44174	siABH4#1 5nM down vs siC 5nM
HMGB2	-3.42835	siABH4#1 5nM down vs siC 5nM
EZH2	-3.39208	siABH4#1 5nM down vs siC 5nM
RTTN	-3.37899	siABH4#1 5nM down vs siC 5nM
SNHG1	-3.37355	siABH4#1 5nM down vs siC 5nM

ZWINT	-3.36117	siABH4#1 5nM down vs siC 5nM
TOPBP1	-3.34041	siABH4#1 5nM down vs siC 5nM
RFC2	-3.33546	siABH4#1 5nM down vs siC 5nM
GINS4	-3.33275	siABH4#1 5nM down vs siC 5nM
TACC3	-3.3232	siABH4#1 5nM down vs siC 5nM
CDKN2C	-3.3139	siABH4#1 5nM down vs siC 5nM
NUCB1	-3.31294	siABH4#1 5nM down vs siC 5nM
ARHGAP11B	-3.31287	siABH4#1 5nM down vs siC 5nM
CCDC18	-3.31163	siABH4#1 5nM down vs siC 5nM
PDK4	-3.30617	siABH4#1 5nM down vs siC 5nM
SMC3	-3.30398	siABH4#1 5nM down vs siC 5nM
CENPO	-3.28991	siABH4#1 5nM down vs siC 5nM
RMI2	-3.2895	siABH4#1 5nM down vs siC 5nM
RFWD3	-3.28861	siABH4#1 5nM down vs siC 5nM
YARS	-3.28853	siABH4#1 5nM down vs siC 5nM
MIR4433A	-3.27701	siABH4#1 5nM down vs siC 5nM
DMC1	-3.27146	siABH4#1 5nM down vs siC 5nM
CENPA	-3.24485	siABH4#1 5nM down vs siC 5nM
GMNN	-3.2306	siABH4#1 5nM down vs siC 5nM
TMEM109	-3.21988	siABH4#1 5nM down vs siC 5nM
SLC31A1	-3.21496	siABH4#1 5nM down vs siC 5nM
CNN2	-3.21176	siABH4#1 5nM down vs siC 5nM
CHAF1A	-3.21042	siABH4#1 5nM down vs siC 5nM
TROAP	-3.19907	siABH4#1 5nM down vs siC 5nM
TTF2	-3.19364	siABH4#1 5nM down vs siC 5nM
CDK2	-3.18786	siABH4#1 5nM down vs siC 5nM
INHBB	-3.17981	siABH4#1 5nM down vs siC 5nM
GINS1	-3.17338	siABH4#1 5nM down vs siC 5nM
RFC5	-3.16633	siABH4#1 5nM down vs siC 5nM
HIST1H4K	-3.1571	siABH4#1 5nM down vs siC 5nM
HIST1H1D	-3.14904	siABH4#1 5nM down vs siC 5nM
CCDC138	-3.12529	siABH4#1 5nM down vs siC 5nM

NASP	-3.12178	siABH4#1 5nM down vs siC 5nM
CENPM	-3.117	siABH4#1 5nM down vs siC 5nM
GPSM2	-3.11245	siABH4#1 5nM down vs siC 5nM
C17orf53	-3.10345	siABH4#1 5nM down vs siC 5nM
MOG	-3.08967	siABH4#1 5nM down vs siC 5nM
ARHGEF39	-3.07774	siABH4#1 5nM down vs siC 5nM
FBXO43	-3.07229	siABH4#1 5nM down vs siC 5nM
EMP2	-3.06387	siABH4#1 5nM down vs siC 5nM
SUV39H2	-3.0486	siABH4#1 5nM down vs siC 5nM
BARD1	-3.04767	siABH4#1 5nM down vs siC 5nM
RMI1	-3.04468	siABH4#1 5nM down vs siC 5nM
AREG	-3.04019	siABH4#1 5nM down vs siC 5nM
CCP110	-3.0226	siABH4#1 5nM down vs siC 5nM
HIST1H3H	-3.01616	siABH4#1 5nM down vs siC 5nM
UBR7	-3.01357	siABH4#1 5nM down vs siC 5nM
DEK	-3.01202	siABH4#1 5nM down vs siC 5nM
HIST2H2AB	-3.00598	siABH4#1 5nM down vs siC 5nM
ZNF738	-3.0046	siABH4#1 5nM down vs siC 5nM
RFC4	-3.00358	siABH4#1 5nM down vs siC 5nM
TRIM59	-2.98077	siABH4#1 5nM down vs siC 5nM
RPL22L1	-2.96591	siABH4#1 5nM down vs siC 5nM
SLFN11	-2.9656	siABH4#1 5nM down vs siC 5nM
MSH6	-2.9654	siABH4#1 5nM down vs siC 5nM
LOC728715	-2.95559	siABH4#1 5nM down vs siC 5nM
MSH2	-2.95554	siABH4#1 5nM down vs siC 5nM
MIR548T	-2.94648	siABH4#1 5nM down vs siC 5nM
FGA	-2.94486	siABH4#1 5nM down vs siC 5nM
CKAP2	-2.94449	siABH4#1 5nM down vs siC 5nM
SNORD18B	-2.94097	siABH4#1 5nM down vs siC 5nM
ARMC6	-2.93663	siABH4#1 5nM down vs siC 5nM
PRAMEF18	-2.93289	siABH4#1 5nM down vs siC 5nM
LBR	-2.93084	siABH4#1 5nM down vs siC 5nM

DLEU2	-2.92431	siABH4#1 5nM down vs siC 5nM
DNMT1	-2.92157	siABH4#1 5nM down vs siC 5nM
H2AFX	-2.91457	siABH4#1 5nM down vs siC 5nM
CCNE1	-2.91155	siABH4#1 5nM down vs siC 5nM
NDC1	-2.90987	siABH4#1 5nM down vs siC 5nM
HAT1	-2.89895	siABH4#1 5nM down vs siC 5nM
NFIL3	-2.89165	siABH4#1 5nM down vs siC 5nM
TIPIN	-2.89066	siABH4#1 5nM down vs siC 5nM
FAM189B	-2.87614	siABH4#1 5nM down vs siC 5nM
THAP10	-2.87321	siABH4#1 5nM down vs siC 5nM
ITGB3BP	-2.86904	siABH4#1 5nM down vs siC 5nM
SUPT16H	-2.86861	siABH4#1 5nM down vs siC 5nM
BRDTP1	-2.86792	siABH4#1 5nM down vs siC 5nM
EHD2	-2.86578	siABH4#1 5nM down vs siC 5nM
SPATA5	-2.85025	siABH4#1 5nM down vs siC 5nM
MT1A	-2.84832	siABH4#1 5nM down vs siC 5nM
RAD18	-2.84656	siABH4#1 5nM down vs siC 5nM
UHRF1	-2.84483	siABH4#1 5nM down vs siC 5nM
PPM1G	-2.84341	siABH4#1 5nM down vs siC 5nM
AREG	-2.82699	siABH4#1 5nM down vs siC 5nM
F2RL2	-2.8264	siABH4#1 5nM down vs siC 5nM
DNA2	-2.81577	siABH4#1 5nM down vs siC 5nM
MIR23C	-2.81238	siABH4#1 5nM down vs siC 5nM
NUCKS1	-2.8088	siABH4#1 5nM down vs siC 5nM
SPTLC3	-2.80653	siABH4#1 5nM down vs siC 5nM
IMPDH1	-2.79889	siABH4#1 5nM down vs siC 5nM
RNF26	-2.79688	siABH4#1 5nM down vs siC 5nM
ECT2	-2.79037	siABH4#1 5nM down vs siC 5nM
HIST1H4C	-2.78565	siABH4#1 5nM down vs siC 5nM
HPRT1	-2.77984	siABH4#1 5nM down vs siC 5nM
G2E3	-2.76302	siABH4#1 5nM down vs siC 5nM
MRPS10	-2.75676	siABH4#1 5nM down vs siC 5nM

SCARNA9	-2.75543	siABH4#1 5nM down vs siC 5nM
MIR4461	-2.75514	siABH4#1 5nM down vs siC 5nM
SNORD19	-2.75042	siABH4#1 5nM down vs siC 5nM
SGOL1-AS1	-2.7426	siABH4#1 5nM down vs siC 5nM
LOC101927746	-2.73875	siABH4#1 5nM down vs siC 5nM
MIR3122	-2.73853	siABH4#1 5nM down vs siC 5nM
GGH	-2.73594	siABH4#1 5nM down vs siC 5nM
H2AFZ	-2.7353	siABH4#1 5nM down vs siC 5nM
MIR924	-2.73425	siABH4#1 5nM down vs siC 5nM
UACA	-2.72459	siABH4#1 5nM down vs siC 5nM
KIFC1	-2.7047	siABH4#1 5nM down vs siC 5nM
ARHGAP29	-2.69963	siABH4#1 5nM down vs siC 5nM
CENPV	-2.69096	siABH4#1 5nM down vs siC 5nM
TIMELESS	-2.69009	siABH4#1 5nM down vs siC 5nM
NUP107	-2.68913	siABH4#1 5nM down vs siC 5nM
PRDX1	-2.68357	siABH4#1 5nM down vs siC 5nM
NRM	-2.67713	siABH4#1 5nM down vs siC 5nM
HAS2	-2.67575	siABH4#1 5nM down vs siC 5nM
GEMIN4	-2.67405	siABH4#1 5nM down vs siC 5nM
TAF5	-2.67161	siABH4#1 5nM down vs siC 5nM
HIST1H4J	-2.66872	siABH4#1 5nM down vs siC 5nM
ULK4P3	-2.66778	siABH4#1 5nM down vs siC 5nM
RAD54B	-2.66669	siABH4#1 5nM down vs siC 5nM
CKS1B	-2.64945	siABH4#1 5nM down vs siC 5nM
BCAT1	-2.64606	siABH4#1 5nM down vs siC 5nM
PHGDH	-2.64577	siABH4#1 5nM down vs siC 5nM
LYAR	-2.64113	siABH4#1 5nM down vs siC 5nM
OR2B6	-2.63837	siABH4#1 5nM down vs siC 5nM
PKMYT1	-2.63339	siABH4#1 5nM down vs siC 5nM
LRRN3	-2.62884	siABH4#1 5nM down vs siC 5nM
PRIM2	-2.62392	siABH4#1 5nM down vs siC 5nM
FEN1	-2.62347	siABH4#1 5nM down vs siC 5nM

KIAA0101	-2.61995	siABH4#1 5nM down vs siC 5nM
CCDC77	-2.61349	siABH4#1 5nM down vs siC 5nM
MPP1	-2.61226	siABH4#1 5nM down vs siC 5nM
GPD2	-2.61095	siABH4#1 5nM down vs siC 5nM
ULK4P1	-2.6091	siABH4#1 5nM down vs siC 5nM
EXOSC8	-2.6016	siABH4#1 5nM down vs siC 5nM
UBL7-AS1	-2.6013	siABH4#1 5nM down vs siC 5nM
CCNE2	-2.58974	siABH4#1 5nM down vs siC 5nM
SLC4A8	-2.58803	siABH4#1 5nM down vs siC 5nM
MIR4659A	-2.58127	siABH4#1 5nM down vs siC 5nM
MLEC	-2.57276	siABH4#1 5nM down vs siC 5nM
CCNYL1	-2.57252	siABH4#1 5nM down vs siC 5nM
IMPA2	-2.56901	siABH4#1 5nM down vs siC 5nM
SNHG5	-2.56795	siABH4#1 5nM down vs siC 5nM
RNASEH2A	-2.55655	siABH4#1 5nM down vs siC 5nM
AGR2	-2.55535	siABH4#1 5nM down vs siC 5nM
ZGRF1	-2.55451	siABH4#1 5nM down vs siC 5nM
LRRC49	-2.55397	siABH4#1 5nM down vs siC 5nM
GPX2	-2.5405	siABH4#1 5nM down vs siC 5nM
CTDNEP1	-2.53886	siABH4#1 5nM down vs siC 5nM
PARP2	-2.53643	siABH4#1 5nM down vs siC 5nM
CDCA7L	-2.53119	siABH4#1 5nM down vs siC 5nM
LOC105379280	-2.52635	siABH4#1 5nM down vs siC 5nM
GEM	-2.52515	siABH4#1 5nM down vs siC 5nM
CREB3L3	-2.51481	siABH4#1 5nM down vs siC 5nM
FANCG	-2.51414	siABH4#1 5nM down vs siC 5nM
GSTCD	-2.51259	siABH4#1 5nM down vs siC 5nM
PPIH	-2.50981	siABH4#1 5nM down vs siC 5nM
ACD	-2.50193	siABH4#1 5nM down vs siC 5nM
CWF19L2	-2.4947	siABH4#1 5nM down vs siC 5nM
RPA2	-2.4916	siABH4#1 5nM down vs siC 5nM
C3orf14	-2.48678	siABH4#1 5nM down vs siC 5nM

SLFN13	-2.48325	siABH4#1 5nM down vs siC 5nM
ASNS	-2.48286	siABH4#1 5nM down vs siC 5nM
UBE2S	-2.47493	siABH4#1 5nM down vs siC 5nM
MIR4328	-2.47445	siABH4#1 5nM down vs siC 5nM
KLHDC3	-2.47339	siABH4#1 5nM down vs siC 5nM
DCLK1	-2.47225	siABH4#1 5nM down vs siC 5nM
ZNF358	-2.46892	siABH4#1 5nM down vs siC 5nM
POC1A	-2.46628	siABH4#1 5nM down vs siC 5nM
TTC30B	-2.46579	siABH4#1 5nM down vs siC 5nM
ADSS	-2.46493	siABH4#1 5nM down vs siC 5nM
PAQR4	-2.46477	siABH4#1 5nM down vs siC 5nM
CKS2	-2.45502	siABH4#1 5nM down vs siC 5nM
LOC102723825	-2.45035	siABH4#1 5nM down vs siC 5nM
TEX30	-2.44815	siABH4#1 5nM down vs siC 5nM
MIS18A	-2.4469	siABH4#1 5nM down vs siC 5nM
DTYMK	-2.4384	siABH4#1 5nM down vs siC 5nM
AUNIP	-2.43806	siABH4#1 5nM down vs siC 5nM
EXOSC9	-2.43455	siABH4#1 5nM down vs siC 5nM
TUBA1B	-2.43426	siABH4#1 5nM down vs siC 5nM
ZNF367	-2.42225	siABH4#1 5nM down vs siC 5nM
DCLRE1A	-2.42137	siABH4#1 5nM down vs siC 5nM
CDCA7	-2.41964	siABH4#1 5nM down vs siC 5nM
AIM1	-2.4182	siABH4#1 5nM down vs siC 5nM
NPR3	-2.41321	siABH4#1 5nM down vs siC 5nM
CENPL	-2.41023	siABH4#1 5nM down vs siC 5nM
BRIX1	-2.40268	siABH4#1 5nM down vs siC 5nM
NUP205	-2.39866	siABH4#1 5nM down vs siC 5nM
MTHFD2	-2.39287	siABH4#1 5nM down vs siC 5nM
LOC81691	-2.39014	siABH4#1 5nM down vs siC 5nM
LOC105377540	-2.38992	siABH4#1 5nM down vs siC 5nM
DNAJC9	-2.3883	siABH4#1 5nM down vs siC 5nM
SLC25A10	-2.38757	siABH4#1 5nM down vs siC 5nM

LIN9	-2.38411	siABH4#1 5nM down vs siC 5nM
EFNB2	-2.37833	siABH4#1 5nM down vs siC 5nM
C17orf58	-2.37792	siABH4#1 5nM down vs siC 5nM
POLR1E	-2.37765	siABH4#1 5nM down vs siC 5nM
TAF6	-2.37763	siABH4#1 5nM down vs siC 5nM
ULK4P1	-2.37322	siABH4#1 5nM down vs siC 5nM
TUBGCP3	-2.36963	siABH4#1 5nM down vs siC 5nM
TBC1D13	-2.36808	siABH4#1 5nM down vs siC 5nM
C19orf48	-2.36747	siABH4#1 5nM down vs siC 5nM
HIST2H2BB	-2.35895	siABH4#1 5nM down vs siC 5nM
MAZ	-2.35893	siABH4#1 5nM down vs siC 5nM
HIST2H2BA	-2.35479	siABH4#1 5nM down vs siC 5nM
MBOAT1	-2.35367	siABH4#1 5nM down vs siC 5nM
TTF2	-2.35265	siABH4#1 5nM down vs siC 5nM
LOC105373547	-2.35023	siABH4#1 5nM down vs siC 5nM
MIR15B	-2.3432	siABH4#1 5nM down vs siC 5nM
RECQL4	-2.34294	siABH4#1 5nM down vs siC 5nM
HIST1H2BI	-2.33872	siABH4#1 5nM down vs siC 5nM
YEATS4	-2.33593	siABH4#1 5nM down vs siC 5nM
PREX1	-2.3351	siABH4#1 5nM down vs siC 5nM
LY6K	-2.3332	siABH4#1 5nM down vs siC 5nM
MIR1244-1	-2.33048	siABH4#1 5nM down vs siC 5nM
MIR1244-1	-2.33048	siABH4#1 5nM down vs siC 5nM
MIR1244-1	-2.33048	siABH4#1 5nM down vs siC 5nM
FAM27E3	-2.32919	siABH4#1 5nM down vs siC 5nM
NUP155	-2.32816	siABH4#1 5nM down vs siC 5nM
GEN1	-2.32178	siABH4#1 5nM down vs siC 5nM
CAPNS1	-2.32109	siABH4#1 5nM down vs siC 5nM
SLC35B2	-2.31673	siABH4#1 5nM down vs siC 5nM
TMPO-AS1	-2.3139	siABH4#1 5nM down vs siC 5nM
DEPDC1-AS1	-2.31351	siABH4#1 5nM down vs siC 5nM
LINC00173	-2.31142	siABH4#1 5nM down vs siC 5nM

RACGAP1	-2.30958	siABH4#1 5nM down vs siC 5nM
NIF3L1	-2.30797	siABH4#1 5nM down vs siC 5nM
CHRFAM7A	-2.307	siABH4#1 5nM down vs siC 5nM
TRAIP	-2.30549	siABH4#1 5nM down vs siC 5nM
ANGPT1	-2.29779	siABH4#1 5nM down vs siC 5nM
BCL2L12	-2.29756	siABH4#1 5nM down vs siC 5nM
LOC102724642	-2.29721	siABH4#1 5nM down vs siC 5nM
ANAPC1	-2.29245	siABH4#1 5nM down vs siC 5nM
CWF19L1	-2.28961	siABH4#1 5nM down vs siC 5nM
HACD2	-2.28423	siABH4#1 5nM down vs siC 5nM
CHEK1	-2.28322	siABH4#1 5nM down vs siC 5nM
ZNF649	-2.27685	siABH4#1 5nM down vs siC 5nM
URM1	-2.27468	siABH4#1 5nM down vs siC 5nM
HIST3H2BB	-2.27215	siABH4#1 5nM down vs siC 5nM
EXO1	-2.26761	siABH4#1 5nM down vs siC 5nM
MPV17L	-2.26365	siABH4#1 5nM down vs siC 5nM
POLR2E	-2.26219	siABH4#1 5nM down vs siC 5nM
CHAC2	-2.25908	siABH4#1 5nM down vs siC 5nM
AVPI1	-2.2571	siABH4#1 5nM down vs siC 5nM
ZNF519	-2.25085	siABH4#1 5nM down vs siC 5nM
MIR516A1	-2.24904	siABH4#1 5nM down vs siC 5nM
LOC105377538	-2.24674	siABH4#1 5nM down vs siC 5nM
FAAP24	-2.24525	siABH4#1 5nM down vs siC 5nM
HIP1	-2.24216	siABH4#1 5nM down vs siC 5nM
SNORD99	-2.23974	siABH4#1 5nM down vs siC 5nM
PSRC1	-2.23955	siABH4#1 5nM down vs siC 5nM
PIF1	-2.23648	siABH4#1 5nM down vs siC 5nM
HIST1H2BO	-2.23337	siABH4#1 5nM down vs siC 5nM
C2orf44	-2.23317	siABH4#1 5nM down vs siC 5nM
SCAMP5	-2.23315	siABH4#1 5nM down vs siC 5nM
GREM1	-2.23288	siABH4#1 5nM down vs siC 5nM
MMD	-2.23072	siABH4#1 5nM down vs siC 5nM

GDAP1	-2.22775	siABH4#1 5nM down vs siC 5nM
POLR3K	-2.22179	siABH4#1 5nM down vs siC 5nM
UBA2	-2.22157	siABH4#1 5nM down vs siC 5nM
LOC730102	-2.21992	siABH4#1 5nM down vs siC 5nM
RNF126	-2.21765	siABH4#1 5nM down vs siC 5nM
LOC105376017	-2.21546	siABH4#1 5nM down vs siC 5nM
CASP8AP2	-2.21244	siABH4#1 5nM down vs siC 5nM
SCARA3	-2.21089	siABH4#1 5nM down vs siC 5nM
POLR3G	-2.20805	siABH4#1 5nM down vs siC 5nM
DDX21	-2.20374	siABH4#1 5nM down vs siC 5nM
MIR548A2	-2.20192	siABH4#1 5nM down vs siC 5nM
SNRPD1	-2.20164	siABH4#1 5nM down vs siC 5nM
LOC101927978	-2.19354	siABH4#1 5nM down vs siC 5nM
PHF19	-2.19128	siABH4#1 5nM down vs siC 5nM
DCLRE1B	-2.18841	siABH4#1 5nM down vs siC 5nM
TIMM21	-2.18722	siABH4#1 5nM down vs siC 5nM
ADPGK	-2.18447	siABH4#1 5nM down vs siC 5nM
NUP88	-2.18224	siABH4#1 5nM down vs siC 5nM
URB2	-2.18217	siABH4#1 5nM down vs siC 5nM
AKAP12	-2.18148	siABH4#1 5nM down vs siC 5nM
ANKRD36C	-2.17639	siABH4#1 5nM down vs siC 5nM
MYBL1	-2.1763	siABH4#1 5nM down vs siC 5nM
RPGRIP1L	-2.17608	siABH4#1 5nM down vs siC 5nM
HIST1H1E	-2.17113	siABH4#1 5nM down vs siC 5nM
LOC100505817	-2.16927	siABH4#1 5nM down vs siC 5nM
ABCG2	-2.16411	siABH4#1 5nM down vs siC 5nM
MAP1A	-2.16074	siABH4#1 5nM down vs siC 5nM
RNF10	-2.15992	siABH4#1 5nM down vs siC 5nM
UPP1	-2.15964	siABH4#1 5nM down vs siC 5nM
CNTRL	-2.15511	siABH4#1 5nM down vs siC 5nM
ABCF2	-2.15324	siABH4#1 5nM down vs siC 5nM
CSE1L	-2.14936	siABH4#1 5nM down vs siC 5nM

CBX3	-2.14014	siABH4#1 5nM down vs siC 5nM
DHFR	-2.13906	siABH4#1 5nM down vs siC 5nM
STMN1	-2.1388	siABH4#1 5nM down vs siC 5nM
MIR4438	-2.13393	siABH4#1 5nM down vs siC 5nM
HIST1H1C	-2.13334	siABH4#1 5nM down vs siC 5nM
PPP2R5D	-2.13228	siABH4#1 5nM down vs siC 5nM
PDS5B	-2.13195	siABH4#1 5nM down vs siC 5nM
HMGN2	-2.1308	siABH4#1 5nM down vs siC 5nM
RCE1	-2.12925	siABH4#1 5nM down vs siC 5nM
GFRA2	-2.12892	siABH4#1 5nM down vs siC 5nM
FGB	-2.11935	siABH4#1 5nM down vs siC 5nM
FLRT3	-2.11495	siABH4#1 5nM down vs siC 5nM
ABCE1	-2.11494	siABH4#1 5nM down vs siC 5nM
DONSON	-2.11479	siABH4#1 5nM down vs siC 5nM
UCHL5	-2.11335	siABH4#1 5nM down vs siC 5nM
SNRPF	-2.11285	siABH4#1 5nM down vs siC 5nM
PIGW	-2.11169	siABH4#1 5nM down vs siC 5nM
GPR19	-2.11056	siABH4#1 5nM down vs siC 5nM
GPX8	-2.10794	siABH4#1 5nM down vs siC 5nM
TUBA3E	-2.10595	siABH4#1 5nM down vs siC 5nM
H2AFV	-2.10284	siABH4#1 5nM down vs siC 5nM
MRPL16	-2.09644	siABH4#1 5nM down vs siC 5nM
IGHV3-38	-2.09641	siABH4#1 5nM down vs siC 5nM
ZNF675	-2.09509	siABH4#1 5nM down vs siC 5nM
FAM72A	-2.09416	siABH4#1 5nM down vs siC 5nM
MIR4279	-2.09126	siABH4#1 5nM down vs siC 5nM
PYCR1	-2.08958	siABH4#1 5nM down vs siC 5nM
TRPA1	-2.08848	siABH4#1 5nM down vs siC 5nM
GJC1	-2.08509	siABH4#1 5nM down vs siC 5nM
LIN52	-2.08427	siABH4#1 5nM down vs siC 5nM
PEF1	-2.08176	siABH4#1 5nM down vs siC 5nM
ANKRD20A1	-2.07968	siABH4#1 5nM down vs siC 5nM

TTF1	-2.07918	siABH4#1 5nM down vs siC 5nM
HSPD1	-2.07864	siABH4#1 5nM down vs siC 5nM
ZNF280C	-2.07692	siABH4#1 5nM down vs siC 5nM
CARM1	-2.07503	siABH4#1 5nM down vs siC 5nM
SLC10A3	-2.07405	siABH4#1 5nM down vs siC 5nM
PGAP2	-2.07367	siABH4#1 5nM down vs siC 5nM
TMEM158	-2.07349	siABH4#1 5nM down vs siC 5nM
ATF6	-2.07299	siABH4#1 5nM down vs siC 5nM
ZNF714	-2.07091	siABH4#1 5nM down vs siC 5nM
SNORD20	-2.06996	siABH4#1 5nM down vs siC 5nM
DPY19L2P1	-2.0685	siABH4#1 5nM down vs siC 5nM
EMC3-AS1	-2.0685	siABH4#1 5nM down vs siC 5nM
RAD1	-2.06259	siABH4#1 5nM down vs siC 5nM
HAUS3	-2.06168	siABH4#1 5nM down vs siC 5nM
TGM2	-2.0573	siABH4#1 5nM down vs siC 5nM
MIR3939	-2.05679	siABH4#1 5nM down vs siC 5nM
HYOU1	-2.05672	siABH4#1 5nM down vs siC 5nM
RIMBP3B	-2.05586	siABH4#1 5nM down vs siC 5nM
LOC105373652	-2.05255	siABH4#1 5nM down vs siC 5nM
TBC1D8	-2.0512	siABH4#1 5nM down vs siC 5nM
IFIT1	-2.05022	siABH4#1 5nM down vs siC 5nM
XPO1	-2.05008	siABH4#1 5nM down vs siC 5nM
CCDC30	-2.04978	siABH4#1 5nM down vs siC 5nM
HAUS1	-2.04977	siABH4#1 5nM down vs siC 5nM
MIR544B	-2.04949	siABH4#1 5nM down vs siC 5nM
MIR4738	-2.04905	siABH4#1 5nM down vs siC 5nM
RPS2P46	-2.04857	siABH4#1 5nM down vs siC 5nM
ZNF430	-2.0471	siABH4#1 5nM down vs siC 5nM
RPL39L	-2.04598	siABH4#1 5nM down vs siC 5nM
CRISPLD2	-2.04591	siABH4#1 5nM down vs siC 5nM
LOC105372906	-2.0449	siABH4#1 5nM down vs siC 5nM
CTNS	-2.04198	siABH4#1 5nM down vs siC 5nM

MTRNR2L2	-2.04176	siABH4#1 5nM down vs siC 5nM
LRRC59	-2.04139	siABH4#1 5nM down vs siC 5nM
ORC3	-2.03946	siABH4#1 5nM down vs siC 5nM
REXO1	-2.03731	siABH4#1 5nM down vs siC 5nM
EBP	-2.03454	siABH4#1 5nM down vs siC 5nM
CD14	-2.03378	siABH4#1 5nM down vs siC 5nM
HSP90AB3P	-2.03276	siABH4#1 5nM down vs siC 5nM
PHF5A	-2.02937	siABH4#1 5nM down vs siC 5nM
EFCAB12	-2.02749	siABH4#1 5nM down vs siC 5nM
NETO2	-2.0268	siABH4#1 5nM down vs siC 5nM
NCR3LG1	-2.02667	siABH4#1 5nM down vs siC 5nM
RPL23AP87	-2.02605	siABH4#1 5nM down vs siC 5nM
ZNF431	-2.02518	siABH4#1 5nM down vs siC 5nM
ZNF229	-2.02406	siABH4#1 5nM down vs siC 5nM
RNU6-72P	-2.02306	siABH4#1 5nM down vs siC 5nM
ALYREF	-2.02291	siABH4#1 5nM down vs siC 5nM
ZNF215	-2.02188	siABH4#1 5nM down vs siC 5nM
MID1	-2.02145	siABH4#1 5nM down vs siC 5nM
REEP4	-2.02012	siABH4#1 5nM down vs siC 5nM
RAF1	-2.01904	siABH4#1 5nM down vs siC 5nM
PCED1B-AS1	-2.01594	siABH4#1 5nM down vs siC 5nM
CHST14	-2.01526	siABH4#1 5nM down vs siC 5nM
PSIP1	-2.01466	siABH4#1 5nM down vs siC 5nM
ATXN7L3B	-2.014	siABH4#1 5nM down vs siC 5nM
ERAL1	-2.0124	siABH4#1 5nM down vs siC 5nM
HNMT	-2.01238	siABH4#1 5nM down vs siC 5nM
CLDN12	-2.01165	siABH4#1 5nM down vs siC 5nM
NR5A2	-2.00787	siABH4#1 5nM down vs siC 5nM
IRS2	-2.00616	siABH4#1 5nM down vs siC 5nM
SERPINB9	-2.00599	siABH4#1 5nM down vs siC 5nM
NUP210	-2.0059	siABH4#1 5nM down vs siC 5nM
MT1X	-2.00589	siABH4#1 5nM down vs siC 5nM

WDYHV1	-2.00387	siABH4#1 5nM down vs siC 5nM
CDT1	-2.00057	siABH4#1 5nM down vs siC 5nM
TBPL1	-2.00048	siABH4#1 5nM down vs siC 5nM
SUZ12	-1.99955	siABH4#1 5nM down vs siC 5nM
ACER3	-1.9988	siABH4#1 5nM down vs siC 5nM
HMGB3	-1.99794	siABH4#1 5nM down vs siC 5nM
FOPNL	-1.9938	siABH4#1 5nM down vs siC 5nM
MPHOSPH6	-1.99036	siABH4#1 5nM down vs siC 5nM
CEP135	-1.98902	siABH4#1 5nM down vs siC 5nM
DIRAS3	-1.98826	siABH4#1 5nM down vs siC 5nM
MAPRE2	-1.98677	siABH4#1 5nM down vs siC 5nM
CRNDE	-1.98613	siABH4#1 5nM down vs siC 5nM
OAS3	-1.98557	siABH4#1 5nM down vs siC 5nM
RPL21P28	-1.98502	siABH4#1 5nM down vs siC 5nM
LSM6	-1.98489	siABH4#1 5nM down vs siC 5nM
HSF2	-1.98343	siABH4#1 5nM down vs siC 5nM
LTN1	-1.98224	siABH4#1 5nM down vs siC 5nM
NOP16	-1.98224	siABH4#1 5nM down vs siC 5nM
C19orf24	-1.9819	siABH4#1 5nM down vs siC 5nM
MAPRE1	-1.98114	siABH4#1 5nM down vs siC 5nM
MIR3685	-1.98043	siABH4#1 5nM down vs siC 5nM
SOX2	-1.97851	siABH4#1 5nM down vs siC 5nM
CORO1A	-1.97843	siABH4#1 5nM down vs siC 5nM
LOC100507209	-1.97685	siABH4#1 5nM down vs siC 5nM
CHD1	-1.97672	siABH4#1 5nM down vs siC 5nM
UMPS	-1.97553	siABH4#1 5nM down vs siC 5nM
ZNF114	-1.97486	siABH4#1 5nM down vs siC 5nM
XPOT	-1.97361	siABH4#1 5nM down vs siC 5nM
RAD51C	-1.97351	siABH4#1 5nM down vs siC 5nM
SCML2	-1.97231	siABH4#1 5nM down vs siC 5nM
PSMB2	-1.97205	siABH4#1 5nM down vs siC 5nM
TPI1P3	-1.97158	siABH4#1 5nM down vs siC 5nM

METTL4	-1.96965	siABH4#1 5nM down vs siC 5nM
TMEM107	-1.96908	siABH4#1 5nM down vs siC 5nM
CCDC189	-1.96763	siABH4#1 5nM down vs siC 5nM
CERS6	-1.96696	siABH4#1 5nM down vs siC 5nM
MIR3198-1	-1.96689	siABH4#1 5nM down vs siC 5nM
SLC25A39	-1.96582	siABH4#1 5nM down vs siC 5nM
PIP4K2C	-1.96346	siABH4#1 5nM down vs siC 5nM
COMMD8	-1.96254	siABH4#1 5nM down vs siC 5nM
CDK5RAP2	-1.96232	siABH4#1 5nM down vs siC 5nM
HMGB3P1	-1.96188	siABH4#1 5nM down vs siC 5nM
RSL24D1	-1.96134	siABH4#1 5nM down vs siC 5nM
SLC51B	-1.96068	siABH4#1 5nM down vs siC 5nM
VBP1	-1.95991	siABH4#1 5nM down vs siC 5nM
E2F2	-1.9599	siABH4#1 5nM down vs siC 5nM
NEDD1	-1.95945	siABH4#1 5nM down vs siC 5nM
HAUS4	-1.95847	siABH4#1 5nM down vs siC 5nM
NUP85	-1.95841	siABH4#1 5nM down vs siC 5nM
CCT4	-1.9579	siABH4#1 5nM down vs siC 5nM
NCLN	-1.95629	siABH4#1 5nM down vs siC 5nM
CEACAM6	-1.95357	siABH4#1 5nM down vs siC 5nM
TRAJ16	-1.95323	siABH4#1 5nM down vs siC 5nM
GPAT3	-1.95273	siABH4#1 5nM down vs siC 5nM
CENPC	-1.95103	siABH4#1 5nM down vs siC 5nM
LOC101929140	-1.95093	siABH4#1 5nM down vs siC 5nM
TDP1	-1.95049	siABH4#1 5nM down vs siC 5nM
CCDC68	-1.94827	siABH4#1 5nM down vs siC 5nM
ARCN1	-1.94777	siABH4#1 5nM down vs siC 5nM
PHB	-1.94756	siABH4#1 5nM down vs siC 5nM
HIST1H2AC	-1.94685	siABH4#1 5nM down vs siC 5nM
LMNB2	-1.94684	siABH4#1 5nM down vs siC 5nM
UBE2I	-1.94615	siABH4#1 5nM down vs siC 5nM
TOE1	-1.94449	siABH4#1 5nM down vs siC 5nM

NSA2	-1.94426	siABH4#1 5nM down vs siC 5nM
RPA1	-1.94358	siABH4#1 5nM down vs siC 5nM
CCDC150	-1.94311	siABH4#1 5nM down vs siC 5nM
DHRS9	-1.9429	siABH4#1 5nM down vs siC 5nM
LOC105372580	-1.94252	siABH4#1 5nM down vs siC 5nM
LINC00261	-1.94234	siABH4#1 5nM down vs siC 5nM
GRB2	-1.9423	siABH4#1 5nM down vs siC 5nM
GLA	-1.9423	siABH4#1 5nM down vs siC 5nM
PUDP	-1.94052	siABH4#1 5nM down vs siC 5nM
GNE	-1.93998	siABH4#1 5nM down vs siC 5nM
UBE2R2	-1.9398	siABH4#1 5nM down vs siC 5nM
EBPL	-1.93833	siABH4#1 5nM down vs siC 5nM
SLC7A5	-1.93674	siABH4#1 5nM down vs siC 5nM
C2orf81	-1.93529	siABH4#1 5nM down vs siC 5nM
NCR3LG1	-1.93508	siABH4#1 5nM down vs siC 5nM
SNORD88B	-1.93441	siABH4#1 5nM down vs siC 5nM
SYNE2	-1.93363	siABH4#1 5nM down vs siC 5nM
MRTO4	-1.9335	siABH4#1 5nM down vs siC 5nM
SCGB1D2	-1.93076	siABH4#1 5nM down vs siC 5nM
DHCR24	-1.93022	siABH4#1 5nM down vs siC 5nM
STAC	-1.92716	siABH4#1 5nM down vs siC 5nM
TTC26	-1.92631	siABH4#1 5nM down vs siC 5nM
NEK4	-1.92623	siABH4#1 5nM down vs siC 5nM
LYPD1	-1.92493	siABH4#1 5nM down vs siC 5nM
CSNK1G2	-1.92473	siABH4#1 5nM down vs siC 5nM
MIR1322	-1.92404	siABH4#1 5nM down vs siC 5nM
SNX12	-1.92383	siABH4#1 5nM down vs siC 5nM
INPP5A	-1.92377	siABH4#1 5nM down vs siC 5nM
SMCHD1	-1.92369	siABH4#1 5nM down vs siC 5nM
RPA3	-1.92365	siABH4#1 5nM down vs siC 5nM
FLJ33534	-1.92346	siABH4#1 5nM down vs siC 5nM
RANBP1	-1.92299	siABH4#1 5nM down vs siC 5nM

TRBV3-1	-1.92261	siABH4#1 5nM down vs siC 5nM
PNO1	-1.92212	siABH4#1 5nM down vs siC 5nM
CDCA4	-1.92201	siABH4#1 5nM down vs siC 5nM
SMG1P7	-1.91972	siABH4#1 5nM down vs siC 5nM
ZBED8	-1.91965	siABH4#1 5nM down vs siC 5nM
LTBP1	-1.91933	siABH4#1 5nM down vs siC 5nM
ARID3B	-1.91781	siABH4#1 5nM down vs siC 5nM
ADGRL4	-1.91773	siABH4#1 5nM down vs siC 5nM
CBX6	-1.91723	siABH4#1 5nM down vs siC 5nM
NUP93	-1.91687	siABH4#1 5nM down vs siC 5nM
TRBV5-6	-1.91658	siABH4#1 5nM down vs siC 5nM
NIPA2	-1.91466	siABH4#1 5nM down vs siC 5nM
AARS	-1.91306	siABH4#1 5nM down vs siC 5nM
ELOVL2	-1.91206	siABH4#1 5nM down vs siC 5nM
HAUS6	-1.90981	siABH4#1 5nM down vs siC 5nM
CEP78	-1.90839	siABH4#1 5nM down vs siC 5nM
U2SURP	-1.90834	siABH4#1 5nM down vs siC 5nM
IPO11	-1.90756	siABH4#1 5nM down vs siC 5nM
CD55	-1.90745	siABH4#1 5nM down vs siC 5nM
ISOC1	-1.90729	siABH4#1 5nM down vs siC 5nM
MRPS11	-1.90633	siABH4#1 5nM down vs siC 5nM
H3F3C	-1.90525	siABH4#1 5nM down vs siC 5nM
PFAS	-1.90294	siABH4#1 5nM down vs siC 5nM
TAF1D	-1.90121	siABH4#1 5nM down vs siC 5nM
SLC7A5P1	-1.89892	siABH4#1 5nM down vs siC 5nM
CDKN2AIPNL	-1.89806	siABH4#1 5nM down vs siC 5nM
GCLC	-1.89788	siABH4#1 5nM down vs siC 5nM
MRGBP	-1.8972	siABH4#1 5nM down vs siC 5nM
METTL10	-1.89673	siABH4#1 5nM down vs siC 5nM
NPAT	-1.89582	siABH4#1 5nM down vs siC 5nM
KDM2A	-1.89566	siABH4#1 5nM down vs siC 5nM
FBL	-1.89431	siABH4#1 5nM down vs siC 5nM

GFPT2	-1.89357	siABH4#1 5nM down vs siC 5nM
GABPB1	-1.89163	siABH4#1 5nM down vs siC 5nM
NUP160	-1.89072	siABH4#1 5nM down vs siC 5nM
TPRKB	-1.89015	siABH4#1 5nM down vs siC 5nM
CEP152	-1.88985	siABH4#1 5nM down vs siC 5nM
SRSF7	-1.88895	siABH4#1 5nM down vs siC 5nM
TEX21P	-1.88893	siABH4#1 5nM down vs siC 5nM
LOC100506473	-1.88888	siABH4#1 5nM down vs siC 5nM
SNRNP40	-1.88814	siABH4#1 5nM down vs siC 5nM
WRAP53	-1.8881	siABH4#1 5nM down vs siC 5nM
BZW2	-1.88793	siABH4#1 5nM down vs siC 5nM
CCL26	-1.88502	siABH4#1 5nM down vs siC 5nM
RBBP8	-1.88484	siABH4#1 5nM down vs siC 5nM
IFI44L	-1.88444	siABH4#1 5nM down vs siC 5nM
EIF1AD	-1.88405	siABH4#1 5nM down vs siC 5nM
PLA2G4A	-1.88322	siABH4#1 5nM down vs siC 5nM
HMGN2P15	-1.88177	siABH4#1 5nM down vs siC 5nM
NELFB	-1.88148	siABH4#1 5nM down vs siC 5nM
RPL14	-1.88147	siABH4#1 5nM down vs siC 5nM
AHR	-1.88065	siABH4#1 5nM down vs siC 5nM
TCP1	-1.87973	siABH4#1 5nM down vs siC 5nM
ALG10	-1.87937	siABH4#1 5nM down vs siC 5nM
CHN1	-1.87814	siABH4#1 5nM down vs siC 5nM
CEP192	-1.87806	siABH4#1 5nM down vs siC 5nM
GPN3	-1.8776	siABH4#1 5nM down vs siC 5nM
CKAP5	-1.87682	siABH4#1 5nM down vs siC 5nM
LOC105370700	-1.87634	siABH4#1 5nM down vs siC 5nM
FBXO27	-1.87571	siABH4#1 5nM down vs siC 5nM
TMEM99	-1.87455	siABH4#1 5nM down vs siC 5nM
LIN54	-1.87397	siABH4#1 5nM down vs siC 5nM
GATSL2	-1.87325	siABH4#1 5nM down vs siC 5nM
POLD1	-1.8732	siABH4#1 5nM down vs siC 5nM

C4orf46	-1.87277	siABH4#1 5nM down vs siC 5nM
SNORD1A	-1.87166	siABH4#1 5nM down vs siC 5nM
ZNF511	-1.87036	siABH4#1 5nM down vs siC 5nM
TGFBR2	-1.86992	siABH4#1 5nM down vs siC 5nM
RPP30	-1.86945	siABH4#1 5nM down vs siC 5nM
ZBTB2	-1.86833	siABH4#1 5nM down vs siC 5nM
PLEKHH2	-1.86789	siABH4#1 5nM down vs siC 5nM
PLCXD1	-1.86773	siABH4#1 5nM down vs siC 5nM
NPM3	-1.86728	siABH4#1 5nM down vs siC 5nM
NUP50	-1.86711	siABH4#1 5nM down vs siC 5nM
HNRNPA1P10	-1.86656	siABH4#1 5nM down vs siC 5nM
GTF3C2	-1.86651	siABH4#1 5nM down vs siC 5nM
GINM1	-1.86545	siABH4#1 5nM down vs siC 5nM
RIOK1	-1.8639	siABH4#1 5nM down vs siC 5nM
COPS8	-1.86371	siABH4#1 5nM down vs siC 5nM
IK	-1.86366	siABH4#1 5nM down vs siC 5nM
SLC29A1	-1.86352	siABH4#1 5nM down vs siC 5nM
UBE2E3	-1.8632	siABH4#1 5nM down vs siC 5nM
POLDIP2	-1.86311	siABH4#1 5nM down vs siC 5nM
CKMT1A	-1.86298	siABH4#1 5nM down vs siC 5nM
HMGXB4	-1.86099	siABH4#1 5nM down vs siC 5nM
MED28	-1.86026	siABH4#1 5nM down vs siC 5nM
GTF2H2	-1.85874	siABH4#1 5nM down vs siC 5nM
NUDT15	-1.85817	siABH4#1 5nM down vs siC 5nM
PMP22	-1.85791	siABH4#1 5nM down vs siC 5nM
FAM122B	-1.85733	siABH4#1 5nM down vs siC 5nM
MYOCD	-1.85669	siABH4#1 5nM down vs siC 5nM
LOC646813	-1.85654	siABH4#1 5nM down vs siC 5nM
LL0XNC01-250H12.3	-1.85472	siABH4#1 5nM down vs siC 5nM
MCM8-AS1	-1.85395	siABH4#1 5nM down vs siC 5nM
ZNF680	-1.85371	siABH4#1 5nM down vs siC 5nM
FANCL	-1.85338	siABH4#1 5nM down vs siC 5nM

CBX5	-1.85256	siABH4#1 5nM down vs siC 5nM
OR2V2	-1.85178	siABH4#1 5nM down vs siC 5nM
NAT10	-1.85129	siABH4#1 5nM down vs siC 5nM
LRRCC1	-1.85013	siABH4#1 5nM down vs siC 5nM
TYMS	-1.8498	siABH4#1 5nM down vs siC 5nM
SPRR2F	-1.84959	siABH4#1 5nM down vs siC 5nM
LOC101559451	-1.84942	siABH4#1 5nM down vs siC 5nM
PKN3	-1.84829	siABH4#1 5nM down vs siC 5nM
NUP214	-1.84727	siABH4#1 5nM down vs siC 5nM
SFRP5	-1.84664	siABH4#1 5nM down vs siC 5nM
PUM3	-1.84622	siABH4#1 5nM down vs siC 5nM
APITD1-CORT	-1.84584	siABH4#1 5nM down vs siC 5nM
SP4	-1.84523	siABH4#1 5nM down vs siC 5nM
SFRP1	-1.8444	siABH4#1 5nM down vs siC 5nM
ARL6IP1	-1.84335	siABH4#1 5nM down vs siC 5nM
NPM1	-1.84296	siABH4#1 5nM down vs siC 5nM
PCNT	-1.84242	siABH4#1 5nM down vs siC 5nM
PTTG2	-1.84184	siABH4#1 5nM down vs siC 5nM
CEP295	-1.84177	siABH4#1 5nM down vs siC 5nM
THOC3	-1.84172	siABH4#1 5nM down vs siC 5nM
ZNF639	-1.8417	siABH4#1 5nM down vs siC 5nM
OR2A1	-1.8417	siABH4#1 5nM down vs siC 5nM
HSD17B11	-1.84158	siABH4#1 5nM down vs siC 5nM
HIST1H3F	-1.84152	siABH4#1 5nM down vs siC 5nM
PIK3R4	-1.84108	siABH4#1 5nM down vs siC 5nM
LOC105370062	-1.84088	siABH4#1 5nM down vs siC 5nM
FOXRED2	-1.83995	siABH4#1 5nM down vs siC 5nM
ICMT	-1.83952	siABH4#1 5nM down vs siC 5nM
JRK	-1.83908	siABH4#1 5nM down vs siC 5nM
RAB31	-1.83904	siABH4#1 5nM down vs siC 5nM
FAM20B	-1.83864	siABH4#1 5nM down vs siC 5nM
EBAG9	-1.8384	siABH4#1 5nM down vs siC 5nM

ZNF544	-1.83717	siABH4#1 5nM down vs siC 5nM
KCTD1	-1.83582	siABH4#1 5nM down vs siC 5nM
ATL2	-1.83281	siABH4#1 5nM down vs siC 5nM
EPRS	-1.83197	siABH4#1 5nM down vs siC 5nM
TRDJ2	-1.83191	siABH4#1 5nM down vs siC 5nM
MIR16-2	-1.83172	siABH4#1 5nM down vs siC 5nM
FAM161A	-1.83152	siABH4#1 5nM down vs siC 5nM
GMPS	-1.83129	siABH4#1 5nM down vs siC 5nM
ZDHHC12	-1.83122	siABH4#1 5nM down vs siC 5nM
GOLGA7B	-1.83097	siABH4#1 5nM down vs siC 5nM
C11orf70	-1.83095	siABH4#1 5nM down vs siC 5nM
ZNF718	-1.83011	siABH4#1 5nM down vs siC 5nM
ZNF141	-1.8291	siABH4#1 5nM down vs siC 5nM
GPATCH1	-1.82804	siABH4#1 5nM down vs siC 5nM
LOC105374980	-1.82737	siABH4#1 5nM down vs siC 5nM
METTL7B	-1.82731	siABH4#1 5nM down vs siC 5nM
MAN1A1	-1.82666	siABH4#1 5nM down vs siC 5nM
JAGN1	-1.82637	siABH4#1 5nM down vs siC 5nM
CDC7	-1.82304	siABH4#1 5nM down vs siC 5nM
OR4S2	-1.82258	siABH4#1 5nM down vs siC 5nM
COA6	-1.81962	siABH4#1 5nM down vs siC 5nM
KAT6A	-1.81872	siABH4#1 5nM down vs siC 5nM
RIF1	-1.81735	siABH4#1 5nM down vs siC 5nM
WHSC1	-1.81704	siABH4#1 5nM down vs siC 5nM
CIB1	-1.81698	siABH4#1 5nM down vs siC 5nM
TRAF7	-1.81692	siABH4#1 5nM down vs siC 5nM
SNORD16	-1.8169	siABH4#1 5nM down vs siC 5nM
HIST2H2AC	-1.81676	siABH4#1 5nM down vs siC 5nM
CDK11A	-1.81654	siABH4#1 5nM down vs siC 5nM
NOTCH3	-1.81631	siABH4#1 5nM down vs siC 5nM
HENMT1	-1.8161	siABH4#1 5nM down vs siC 5nM
RNPS1	-1.81467	siABH4#1 5nM down vs siC 5nM

SLC7A1	-1.81387	siABH4#1 5nM down vs siC 5nM
SNORD14E	-1.81371	siABH4#1 5nM down vs siC 5nM
EMP1	-1.81366	siABH4#1 5nM down vs siC 5nM
UBXN2B	-1.81311	siABH4#1 5nM down vs siC 5nM
GGA2	-1.81269	siABH4#1 5nM down vs siC 5nM
SNORD75	-1.81159	siABH4#1 5nM down vs siC 5nM
MIR1262	-1.81134	siABH4#1 5nM down vs siC 5nM
SAE1	-1.81101	siABH4#1 5nM down vs siC 5nM
NOP58	-1.8107	siABH4#1 5nM down vs siC 5nM
NUDCD1	-1.80991	siABH4#1 5nM down vs siC 5nM
LOC105377684	-1.80931	siABH4#1 5nM down vs siC 5nM
PDE4B	-1.80878	siABH4#1 5nM down vs siC 5nM
NAP1L2	-1.80814	siABH4#1 5nM down vs siC 5nM
INPP5B	-1.80754	siABH4#1 5nM down vs siC 5nM
MIR4301	-1.80745	siABH4#1 5nM down vs siC 5nM
MIR1278	-1.80693	siABH4#1 5nM down vs siC 5nM
HLA-DRA	-1.80676	siABH4#1 5nM down vs siC 5nM
FAM69A	-1.8062	siABH4#1 5nM down vs siC 5nM
ABT1	-1.80582	siABH4#1 5nM down vs siC 5nM
MLH1	-1.80557	siABH4#1 5nM down vs siC 5nM
FCGR3A	-1.80376	siABH4#1 5nM down vs siC 5nM
APH1A	-1.80308	siABH4#1 5nM down vs siC 5nM
NUP37	-1.80275	siABH4#1 5nM down vs siC 5nM
SNORD101	-1.80244	siABH4#1 5nM down vs siC 5nM
KCTD6	-1.80241	siABH4#1 5nM down vs siC 5nM
PIGA	-1.80238	siABH4#1 5nM down vs siC 5nM
MIR320D1	-1.8023	siABH4#1 5nM down vs siC 5nM
ST6GAL2	-1.80209	siABH4#1 5nM down vs siC 5nM
MCMBP	-1.80172	siABH4#1 5nM down vs siC 5nM
RPS2	-1.80167	siABH4#1 5nM down vs siC 5nM
EVA1C	-1.80152	siABH4#1 5nM down vs siC 5nM
OR51A4	-1.80024	siABH4#1 5nM down vs siC 5nM

MIR4275	-1.79956	siABH4#1 5nM down vs siC 5nM
LINC00266-3	-1.79898	siABH4#1 5nM down vs siC 5nM
PABPC4	-1.79878	siABH4#1 5nM down vs siC 5nM
SNORD70	-1.79774	siABH4#1 5nM down vs siC 5nM
TAF1D	-1.79704	siABH4#1 5nM down vs siC 5nM
TGFB2-AS1	-1.79684	siABH4#1 5nM down vs siC 5nM
LOC105370288	-1.79665	siABH4#1 5nM down vs siC 5nM
SNX16	-1.79615	siABH4#1 5nM down vs siC 5nM
IL22RA1	-1.79531	siABH4#1 5nM down vs siC 5nM
ANP32B	-1.79466	siABH4#1 5nM down vs siC 5nM
MMP3	-1.79418	siABH4#1 5nM down vs siC 5nM
MLLT11	-1.79412	siABH4#1 5nM down vs siC 5nM
NOLC1	-1.79403	siABH4#1 5nM down vs siC 5nM
CEP76	-1.79389	siABH4#1 5nM down vs siC 5nM
TUBD1	-1.79365	siABH4#1 5nM down vs siC 5nM
TTPA	-1.79365	siABH4#1 5nM down vs siC 5nM
FAR2P3	-1.79356	siABH4#1 5nM down vs siC 5nM
INTS2	-1.7932	siABH4#1 5nM down vs siC 5nM
POP1	-1.79303	siABH4#1 5nM down vs siC 5nM
PSMG1	-1.79192	siABH4#1 5nM down vs siC 5nM
OR4K1	-1.79182	siABH4#1 5nM down vs siC 5nM
CHSY1	-1.79166	siABH4#1 5nM down vs siC 5nM
LCORL	-1.79138	siABH4#1 5nM down vs siC 5nM
SSX2IP	-1.79093	siABH4#1 5nM down vs siC 5nM
RAN	-1.79078	siABH4#1 5nM down vs siC 5nM
RPS28	-1.79048	siABH4#1 5nM down vs siC 5nM
MIR520E	-1.79008	siABH4#1 5nM down vs siC 5nM
OR5K3	-1.78984	siABH4#1 5nM down vs siC 5nM
STRA13	-1.78983	siABH4#1 5nM down vs siC 5nM
GUSBP3	-1.78938	siABH4#1 5nM down vs siC 5nM
OR2M2	-1.78828	siABH4#1 5nM down vs siC 5nM
UTP15	-1.78825	siABH4#1 5nM down vs siC 5nM

SUGT1	-1.78735	siABH4#1 5nM down vs siC 5nM
RSRC2	-1.78632	siABH4#1 5nM down vs siC 5nM
ZMYND19	-1.78624	siABH4#1 5nM down vs siC 5nM
SFXN1	-1.78609	siABH4#1 5nM down vs siC 5nM
PRPF4	-1.78559	siABH4#1 5nM down vs siC 5nM
NFATC2	-1.78532	siABH4#1 5nM down vs siC 5nM
TERT	-1.78532	siABH4#1 5nM down vs siC 5nM
TCOF1	-1.78518	siABH4#1 5nM down vs siC 5nM
NSMCE4A	-1.78486	siABH4#1 5nM down vs siC 5nM
TMEM116	-1.78465	siABH4#1 5nM down vs siC 5nM
DDX46	-1.78366	siABH4#1 5nM down vs siC 5nM
PPARGC1A	-1.78301	siABH4#1 5nM down vs siC 5nM
UPF2	-1.78209	siABH4#1 5nM down vs siC 5nM
RHNO1	-1.78176	siABH4#1 5nM down vs siC 5nM
MRPL11	-1.78148	siABH4#1 5nM down vs siC 5nM
SLC38A1	-1.78045	siABH4#1 5nM down vs siC 5nM
ERI2	-1.78015	siABH4#1 5nM down vs siC 5nM
KPNA2	-1.7797	siABH4#1 5nM down vs siC 5nM
TRAPPC13	-1.77962	siABH4#1 5nM down vs siC 5nM
LOC101927267	-1.77808	siABH4#1 5nM down vs siC 5nM
ESF1	-1.77785	siABH4#1 5nM down vs siC 5nM
FAM72B	-1.77715	siABH4#1 5nM down vs siC 5nM
CLCNKA	-1.77611	siABH4#1 5nM down vs siC 5nM
ZNF239	-1.77517	siABH4#1 5nM down vs siC 5nM
AP4S1	-1.77454	siABH4#1 5nM down vs siC 5nM
STK17B	-1.77394	siABH4#1 5nM down vs siC 5nM
EIF4A3	-1.77372	siABH4#1 5nM down vs siC 5nM
PDSS1	-1.77339	siABH4#1 5nM down vs siC 5nM
HLTF	-1.77274	siABH4#1 5nM down vs siC 5nM
TAF5L	-1.77265	siABH4#1 5nM down vs siC 5nM
UTP11L	-1.77255	siABH4#1 5nM down vs siC 5nM
C1QTNF9B-AS1	-1.77173	siABH4#1 5nM down vs siC 5nM

CHMP6	-1.77147	siABH4#1 5nM down vs siC 5nM
BAGE2	-1.77104	siABH4#1 5nM down vs siC 5nM
RCC1	-1.77078	siABH4#1 5nM down vs siC 5nM
ACTL6A	-1.77064	siABH4#1 5nM down vs siC 5nM
PXK	-1.76937	siABH4#1 5nM down vs siC 5nM
KIAA0586	-1.7688	siABH4#1 5nM down vs siC 5nM
DNTTIP2	-1.76839	siABH4#1 5nM down vs siC 5nM
EML4	-1.76793	siABH4#1 5nM down vs siC 5nM
RSBN1L	-1.76782	siABH4#1 5nM down vs siC 5nM
EIF1AY	-1.76525	siABH4#1 5nM down vs siC 5nM
NUP153	-1.76494	siABH4#1 5nM down vs siC 5nM
DCAF16	-1.76403	siABH4#1 5nM down vs siC 5nM
MTPAP	-1.76358	siABH4#1 5nM down vs siC 5nM
LAMTOR3	-1.76335	siABH4#1 5nM down vs siC 5nM
TLN1	-1.76319	siABH4#1 5nM down vs siC 5nM
ZNF107	-1.76279	siABH4#1 5nM down vs siC 5nM
SLC25A44	-1.76227	siABH4#1 5nM down vs siC 5nM
ZNF507	-1.76132	siABH4#1 5nM down vs siC 5nM
CASP2	-1.76126	siABH4#1 5nM down vs siC 5nM
MB21D1	-1.76039	siABH4#1 5nM down vs siC 5nM
LOC105371443	-1.76019	siABH4#1 5nM down vs siC 5nM
SMARCAD1	-1.75997	siABH4#1 5nM down vs siC 5nM
TBC1D3P1-DHX40P1	-1.75977	siABH4#1 5nM down vs siC 5nM
RBM25	-1.75952	siABH4#1 5nM down vs siC 5nM
RBM15	-1.75927	siABH4#1 5nM down vs siC 5nM
RNF4	-1.75925	siABH4#1 5nM down vs siC 5nM
HIST1H3A	-1.75865	siABH4#1 5nM down vs siC 5nM
ZNF530	-1.75855	siABH4#1 5nM down vs siC 5nM
B4GALT2	-1.75848	siABH4#1 5nM down vs siC 5nM
CEP57	-1.75814	siABH4#1 5nM down vs siC 5nM
NMB	-1.75787	siABH4#1 5nM down vs siC 5nM
HSPA1B	-1.75742	siABH4#1 5nM down vs siC 5nM

PDK3	-1.75647	siABH4#1 5nM down vs siC 5nM
LOC105375785	-1.75635	siABH4#1 5nM down vs siC 5nM
HNRNPA2B1	-1.75445	siABH4#1 5nM down vs siC 5nM
PIH1D2	-1.75435	siABH4#1 5nM down vs siC 5nM
NHLRC2	-1.7537	siABH4#1 5nM down vs siC 5nM
SS18L2	-1.75369	siABH4#1 5nM down vs siC 5nM
BAZ1B	-1.7536	siABH4#1 5nM down vs siC 5nM
LOC105377558	-1.75319	siABH4#1 5nM down vs siC 5nM
POLR2D	-1.75303	siABH4#1 5nM down vs siC 5nM
ANKRD13A	-1.75269	siABH4#1 5nM down vs siC 5nM
ZNF735	-1.75226	siABH4#1 5nM down vs siC 5nM
ZNF670	-1.75187	siABH4#1 5nM down vs siC 5nM
ARHGAP33	-1.75158	siABH4#1 5nM down vs siC 5nM
MCTS2P	-1.75122	siABH4#1 5nM down vs siC 5nM
HECTD3	-1.75018	siABH4#1 5nM down vs siC 5nM
STT3B	-1.74834	siABH4#1 5nM down vs siC 5nM
VPS29	-1.748	siABH4#1 5nM down vs siC 5nM
SENP1	-1.74789	siABH4#1 5nM down vs siC 5nM
STK32B	-1.74787	siABH4#1 5nM down vs siC 5nM
GH1	-1.74664	siABH4#1 5nM down vs siC 5nM
MRPL15	-1.74593	siABH4#1 5nM down vs siC 5nM
HTR3D	-1.74469	siABH4#1 5nM down vs siC 5nM
HSPA1A	-1.74327	siABH4#1 5nM down vs siC 5nM
ZW10	-1.74252	siABH4#1 5nM down vs siC 5nM
C1orf174	-1.74185	siABH4#1 5nM down vs siC 5nM
CCNC	-1.74137	siABH4#1 5nM down vs siC 5nM
LOC105372158	-1.74005	siABH4#1 5nM down vs siC 5nM
CEP44	-1.73965	siABH4#1 5nM down vs siC 5nM
SEH1L	-1.73893	siABH4#1 5nM down vs siC 5nM
CYBRD1	-1.73876	siABH4#1 5nM down vs siC 5nM
C2CD5	-1.73863	siABH4#1 5nM down vs siC 5nM
CALM3	-1.73824	siABH4#1 5nM down vs siC 5nM

FAM35DP	-1.73806	siABH4#1 5nM down vs siC 5nM
SAAL1	-1.73769	siABH4#1 5nM down vs siC 5nM
TBL1X	-1.73769	siABH4#1 5nM down vs siC 5nM
SNORD11	-1.73751	siABH4#1 5nM down vs siC 5nM
TPP2	-1.73727	siABH4#1 5nM down vs siC 5nM
APOLD1	-1.73717	siABH4#1 5nM down vs siC 5nM
MED31	-1.73714	siABH4#1 5nM down vs siC 5nM
SMG8	-1.73555	siABH4#1 5nM down vs siC 5nM
KIAA1715	-1.73442	siABH4#1 5nM down vs siC 5nM
MZT1	-1.73434	siABH4#1 5nM down vs siC 5nM
AMD1	-1.73433	siABH4#1 5nM down vs siC 5nM
MIR571	-1.73398	siABH4#1 5nM down vs siC 5nM
SLC35A3	-1.7339	siABH4#1 5nM down vs siC 5nM
JMJD6	-1.73324	siABH4#1 5nM down vs siC 5nM
RAD21	-1.73247	siABH4#1 5nM down vs siC 5nM
UTP20	-1.73229	siABH4#1 5nM down vs siC 5nM
GRB14	-1.73034	siABH4#1 5nM down vs siC 5nM
YWHAH	-1.72931	siABH4#1 5nM down vs siC 5nM
KRTAP29-1	-1.72927	siABH4#1 5nM down vs siC 5nM
MFSD3	-1.72862	siABH4#1 5nM down vs siC 5nM
HOXA2	-1.72831	siABH4#1 5nM down vs siC 5nM
LOC648570	-1.72623	siABH4#1 5nM down vs siC 5nM
COPS7B	-1.72611	siABH4#1 5nM down vs siC 5nM
FLOT2	-1.72494	siABH4#1 5nM down vs siC 5nM
SNHG6	-1.72448	siABH4#1 5nM down vs siC 5nM
C2orf69	-1.72437	siABH4#1 5nM down vs siC 5nM
CCT8	-1.72433	siABH4#1 5nM down vs siC 5nM
SNRPEP2	-1.72431	siABH4#1 5nM down vs siC 5nM
CTSLP2	-1.72348	siABH4#1 5nM down vs siC 5nM
CTSLP2	-1.72348	siABH4#1 5nM down vs siC 5nM
TRERF1	-1.72238	siABH4#1 5nM down vs siC 5nM
HIST2H2AA4	-1.7223	siABH4#1 5nM down vs siC 5nM

RNF219	-1.72229	siABH4#1 5nM down vs siC 5nM
C9orf91	-1.72211	siABH4#1 5nM down vs siC 5nM
CYP2C18	-1.72197	siABH4#1 5nM down vs siC 5nM
DUT	-1.72181	siABH4#1 5nM down vs siC 5nM
PTCH1	-1.72082	siABH4#1 5nM down vs siC 5nM
FN3KRP	-1.72037	siABH4#1 5nM down vs siC 5nM
EIF2A	-1.72014	siABH4#1 5nM down vs siC 5nM
PHYHIPL	-1.7201	siABH4#1 5nM down vs siC 5nM
FAM27E3	-1.71841	siABH4#1 5nM down vs siC 5nM
CRLF3	-1.71819	siABH4#1 5nM down vs siC 5nM
PDE12	-1.71802	siABH4#1 5nM down vs siC 5nM
DHX15	-1.7164	siABH4#1 5nM down vs siC 5nM
TIMM10	-1.71622	siABH4#1 5nM down vs siC 5nM
SLITRK6	-1.71587	siABH4#1 5nM down vs siC 5nM
CEBPZ	-1.71545	siABH4#1 5nM down vs siC 5nM
FANCE	-1.71533	siABH4#1 5nM down vs siC 5nM
SNRNP25	-1.71502	siABH4#1 5nM down vs siC 5nM
GTPBP8	-1.71497	siABH4#1 5nM down vs siC 5nM
NOP56	-1.71491	siABH4#1 5nM down vs siC 5nM
FKBP5	-1.71484	siABH4#1 5nM down vs siC 5nM
ATG13	-1.71482	siABH4#1 5nM down vs siC 5nM
HSPH1	-1.71475	siABH4#1 5nM down vs siC 5nM
RBBP5	-1.7144	siABH4#1 5nM down vs siC 5nM
RPS6KA3	-1.71397	siABH4#1 5nM down vs siC 5nM
CNTLN	-1.71358	siABH4#1 5nM down vs siC 5nM
F2RL1	-1.71343	siABH4#1 5nM down vs siC 5nM
AMMECR1L	-1.71249	siABH4#1 5nM down vs siC 5nM
HSPA14	-1.71233	siABH4#1 5nM down vs siC 5nM
CYB5B	-1.71225	siABH4#1 5nM down vs siC 5nM
FIGN	-1.71221	siABH4#1 5nM down vs siC 5nM
BVES-AS1	-1.71207	siABH4#1 5nM down vs siC 5nM
LOC105376085	-1.71064	siABH4#1 5nM down vs siC 5nM

TM4SF1-AS1	-1.71037	siABH4#1 5nM down vs siC 5nM
CLN6	-1.71017	siABH4#1 5nM down vs siC 5nM
AKAP11	-1.70995	siABH4#1 5nM down vs siC 5nM
INTS7	-1.70965	siABH4#1 5nM down vs siC 5nM
HAUS7	-1.70961	siABH4#1 5nM down vs siC 5nM
C5AR2	-1.7096	siABH4#1 5nM down vs siC 5nM
SCARNA9L	-1.70757	siABH4#1 5nM down vs siC 5nM
MPHOSPH9	-1.70754	siABH4#1 5nM down vs siC 5nM
OR6C2	-1.70714	siABH4#1 5nM down vs siC 5nM
HNRNPH3	-1.70691	siABH4#1 5nM down vs siC 5nM
TAF1D	-1.7068	siABH4#1 5nM down vs siC 5nM
TMEM237	-1.70608	siABH4#1 5nM down vs siC 5nM
LINC00581	-1.70592	siABH4#1 5nM down vs siC 5nM
RANGAP1	-1.70587	siABH4#1 5nM down vs siC 5nM
CTNNAL1	-1.70571	siABH4#1 5nM down vs siC 5nM
DARS2	-1.70543	siABH4#1 5nM down vs siC 5nM
EREG	-1.70502	siABH4#1 5nM down vs siC 5nM
SLC25A19	-1.70309	siABH4#1 5nM down vs siC 5nM
CETN3	-1.70238	siABH4#1 5nM down vs siC 5nM
ICE1	-1.70182	siABH4#1 5nM down vs siC 5nM
ATP2A1-AS1	-1.70169	siABH4#1 5nM down vs siC 5nM
WHAMMP1	-1.70164	siABH4#1 5nM down vs siC 5nM
KPNA4	-1.69989	siABH4#1 5nM down vs siC 5nM
PPIG	-1.69988	siABH4#1 5nM down vs siC 5nM
TAF1A	-1.69946	siABH4#1 5nM down vs siC 5nM
NDUFAB1	-1.69942	siABH4#1 5nM down vs siC 5nM
TMEM216	-1.69896	siABH4#1 5nM down vs siC 5nM
THOC6	-1.69822	siABH4#1 5nM down vs siC 5nM
ERH	-1.69808	siABH4#1 5nM down vs siC 5nM
CABLES1	-1.69787	siABH4#1 5nM down vs siC 5nM
POLR1E	-1.6976	siABH4#1 5nM down vs siC 5nM
MGC27382	-1.69751	siABH4#1 5nM down vs siC 5nM

LOC105373305	-1.69689	siABH4#1 5nM down vs siC 5nM
CABLES2	-1.696	siABH4#1 5nM down vs siC 5nM
CPLX2	-1.69588	siABH4#1 5nM down vs siC 5nM
ZFAND1	-1.69569	siABH4#1 5nM down vs siC 5nM
ZRANB2-AS2	-1.69541	siABH4#1 5nM down vs siC 5nM
CACNG7	-1.6942	siABH4#1 5nM down vs siC 5nM
TRA2B	-1.69376	siABH4#1 5nM down vs siC 5nM
FH	-1.69314	siABH4#1 5nM down vs siC 5nM
EED	-1.69207	siABH4#1 5nM down vs siC 5nM
OR4C16	-1.69163	siABH4#1 5nM down vs siC 5nM
CDK7	-1.69163	siABH4#1 5nM down vs siC 5nM
FKBP1B	-1.69157	siABH4#1 5nM down vs siC 5nM
SNORD104	-1.69052	siABH4#1 5nM down vs siC 5nM
CHAMP1	-1.68996	siABH4#1 5nM down vs siC 5nM
LOC105376207	-1.68929	siABH4#1 5nM down vs siC 5nM
UBE2NL	-1.6891	siABH4#1 5nM down vs siC 5nM
ZNF833P	-1.68907	siABH4#1 5nM down vs siC 5nM
CLEC2B	-1.6883	siABH4#1 5nM down vs siC 5nM
ADRA1B	-1.68824	siABH4#1 5nM down vs siC 5nM
KCTD2	-1.68814	siABH4#1 5nM down vs siC 5nM
NR0B1	-1.68745	siABH4#1 5nM down vs siC 5nM
SRBD1	-1.6871	siABH4#1 5nM down vs siC 5nM
THRAP3	-1.6869	siABH4#1 5nM down vs siC 5nM
LOC105379049	-1.68669	siABH4#1 5nM down vs siC 5nM
BRI3BP	-1.68651	siABH4#1 5nM down vs siC 5nM
KRAS	-1.68635	siABH4#1 5nM down vs siC 5nM
SLC25A24	-1.68628	siABH4#1 5nM down vs siC 5nM
TNPO3	-1.68624	siABH4#1 5nM down vs siC 5nM
RARS	-1.68601	siABH4#1 5nM down vs siC 5nM
DNLZ	-1.68568	siABH4#1 5nM down vs siC 5nM
LOC105378859	-1.68525	siABH4#1 5nM down vs siC 5nM
LOC105375321	-1.68418	siABH4#1 5nM down vs siC 5nM

TAF1D	-1.68363	siABH4#1 5nM down vs siC 5nM
CBX2	-1.68337	siABH4#1 5nM down vs siC 5nM
CASC3	-1.68292	siABH4#1 5nM down vs siC 5nM
NUP133	-1.6826	siABH4#1 5nM down vs siC 5nM
MIR3671	-1.68183	siABH4#1 5nM down vs siC 5nM
FARSB	-1.68178	siABH4#1 5nM down vs siC 5nM
TM4SF1	-1.68176	siABH4#1 5nM down vs siC 5nM
MTF2	-1.68157	siABH4#1 5nM down vs siC 5nM
WDR75	-1.68119	siABH4#1 5nM down vs siC 5nM
KRR1	-1.68076	siABH4#1 5nM down vs siC 5nM
SSX1	-1.68041	siABH4#1 5nM down vs siC 5nM
SULT2A1	-1.68032	siABH4#1 5nM down vs siC 5nM
AHSA1	-1.67936	siABH4#1 5nM down vs siC 5nM
LOC100509780	-1.67935	siABH4#1 5nM down vs siC 5nM
NSUN2	-1.67932	siABH4#1 5nM down vs siC 5nM
ZYG11A	-1.67877	siABH4#1 5nM down vs siC 5nM
LINC01043	-1.67871	siABH4#1 5nM down vs siC 5nM
DCAF15	-1.67831	siABH4#1 5nM down vs siC 5nM
DKC1	-1.6781	siABH4#1 5nM down vs siC 5nM
ETV4	-1.67801	siABH4#1 5nM down vs siC 5nM
PCMT1	-1.67737	siABH4#1 5nM down vs siC 5nM
TOMM70A	-1.67731	siABH4#1 5nM down vs siC 5nM
PPID	-1.67715	siABH4#1 5nM down vs siC 5nM
LOC100128242	-1.67703	siABH4#1 5nM down vs siC 5nM
SEP15	-1.67695	siABH4#1 5nM down vs siC 5nM
RBBP7	-1.67676	siABH4#1 5nM down vs siC 5nM
AHCTF1	-1.67666	siABH4#1 5nM down vs siC 5nM
CSGALNACT1	-1.67632	siABH4#1 5nM down vs siC 5nM
ELOVL6	-1.67629	siABH4#1 5nM down vs siC 5nM
PRKAR2B	-1.67611	siABH4#1 5nM down vs siC 5nM
MYEF2	-1.67545	siABH4#1 5nM down vs siC 5nM
LOC105374876	-1.67491	siABH4#1 5nM down vs siC 5nM

EFCAB7	-1.67395	siABH4#1 5nM down vs siC 5nM
RNVU1-19	-1.67388	siABH4#1 5nM down vs siC 5nM
LRRC40	-1.67385	siABH4#1 5nM down vs siC 5nM
LILRB1	-1.6737	siABH4#1 5nM down vs siC 5nM
RNF43	-1.67302	siABH4#1 5nM down vs siC 5nM
EHD4	-1.67289	siABH4#1 5nM down vs siC 5nM
MEN1	-1.6723	siABH4#1 5nM down vs siC 5nM
CDC25B	-1.67222	siABH4#1 5nM down vs siC 5nM
NAA16	-1.67199	siABH4#1 5nM down vs siC 5nM
RBM48	-1.67198	siABH4#1 5nM down vs siC 5nM
NUP188	-1.67147	siABH4#1 5nM down vs siC 5nM
ROPN1L	-1.67129	siABH4#1 5nM down vs siC 5nM
MIR590	-1.67113	siABH4#1 5nM down vs siC 5nM
TMEM138	-1.67092	siABH4#1 5nM down vs siC 5nM
PAGE3	-1.67076	siABH4#1 5nM down vs siC 5nM
RDM1	-1.67066	siABH4#1 5nM down vs siC 5nM
RECQL	-1.6705	siABH4#1 5nM down vs siC 5nM
ZC3H13	-1.67003	siABH4#1 5nM down vs siC 5nM
PHTF2	-1.66993	siABH4#1 5nM down vs siC 5nM
TSPAN13	-1.66959	siABH4#1 5nM down vs siC 5nM
PSMD11	-1.66954	siABH4#1 5nM down vs siC 5nM
C19orf47	-1.66937	siABH4#1 5nM down vs siC 5nM
AGFG2	-1.66778	siABH4#1 5nM down vs siC 5nM
CLEC4GP1	-1.66744	siABH4#1 5nM down vs siC 5nM
LNP1	-1.66711	siABH4#1 5nM down vs siC 5nM
MRE11A	-1.66711	siABH4#1 5nM down vs siC 5nM
TM4SF20	-1.6671	siABH4#1 5nM down vs siC 5nM
SSRP1	-1.66567	siABH4#1 5nM down vs siC 5nM
SIRT1	-1.66556	siABH4#1 5nM down vs siC 5nM
WBP11	-1.66416	siABH4#1 5nM down vs siC 5nM
GPATCH11	-1.66372	siABH4#1 5nM down vs siC 5nM
TBC1D31	-1.66361	siABH4#1 5nM down vs siC 5nM

UBTD2	-1.66345	siABH4#1 5nM down vs siC 5nM
LOC105375760	-1.66272	siABH4#1 5nM down vs siC 5nM
RPF2	-1.66249	siABH4#1 5nM down vs siC 5nM
NOL11	-1.66246	siABH4#1 5nM down vs siC 5nM
FAM96A	-1.66213	siABH4#1 5nM down vs siC 5nM
NSUN6	-1.66212	siABH4#1 5nM down vs siC 5nM
RQCD1	-1.66179	siABH4#1 5nM down vs siC 5nM
DCK	-1.66166	siABH4#1 5nM down vs siC 5nM
THUMPD1	-1.66149	siABH4#1 5nM down vs siC 5nM
MED4	-1.66119	siABH4#1 5nM down vs siC 5nM
CA5BP1	-1.66115	siABH4#1 5nM down vs siC 5nM
SNHG17	-1.66032	siABH4#1 5nM down vs siC 5nM
MANF	-1.65981	siABH4#1 5nM down vs siC 5nM
MYO19	-1.65966	siABH4#1 5nM down vs siC 5nM
LSM5	-1.65931	siABH4#1 5nM down vs siC 5nM
LOC101929112	-1.65889	siABH4#1 5nM down vs siC 5nM
LOC101927575	-1.65785	siABH4#1 5nM down vs siC 5nM
SF3B3	-1.65734	siABH4#1 5nM down vs siC 5nM
BMPR1B-AS1	-1.65704	siABH4#1 5nM down vs siC 5nM
YEATS4	-1.65677	siABH4#1 5nM down vs siC 5nM
RFXAP	-1.65642	siABH4#1 5nM down vs siC 5nM
FERMT1	-1.65602	siABH4#1 5nM down vs siC 5nM
MIR378D2	-1.6556	siABH4#1 5nM down vs siC 5nM
LOC100132612	-1.6552	siABH4#1 5nM down vs siC 5nM
MIR1224	-1.65498	siABH4#1 5nM down vs siC 5nM
LOC101928105	-1.65402	siABH4#1 5nM down vs siC 5nM
THOC7	-1.65386	siABH4#1 5nM down vs siC 5nM
GAS5	-1.65369	siABH4#1 5nM down vs siC 5nM
HEATR1	-1.65363	siABH4#1 5nM down vs siC 5nM
LOC105372009	-1.65358	siABH4#1 5nM down vs siC 5nM
LOC102724479	-1.6533	siABH4#1 5nM down vs siC 5nM
DCUN1D5	-1.65311	siABH4#1 5nM down vs siC 5nM

TAF9B	-1.65299	siABH4#1 5nM down vs siC 5nM
PPME1	-1.6525	siABH4#1 5nM down vs siC 5nM
MYADM	-1.65201	siABH4#1 5nM down vs siC 5nM
SLC4A9	-1.65186	siABH4#1 5nM down vs siC 5nM
BFAR	-1.65182	siABH4#1 5nM down vs siC 5nM
CNTNAP5	-1.65182	siABH4#1 5nM down vs siC 5nM
BLMH	-1.65168	siABH4#1 5nM down vs siC 5nM
BCL2L11	-1.65066	siABH4#1 5nM down vs siC 5nM
SART3	-1.65059	siABH4#1 5nM down vs siC 5nM
ITGA4	-1.6499	siABH4#1 5nM down vs siC 5nM
LOC105375675	-1.64906	siABH4#1 5nM down vs siC 5nM
PNPT1	-1.64868	siABH4#1 5nM down vs siC 5nM
NELFCD	-1.64851	siABH4#1 5nM down vs siC 5nM
CEND1	-1.64848	siABH4#1 5nM down vs siC 5nM
GTF2H2	-1.64828	siABH4#1 5nM down vs siC 5nM
MAGEB2	-1.64784	siABH4#1 5nM down vs siC 5nM
SSSCA1	-1.64773	siABH4#1 5nM down vs siC 5nM
CCL11	-1.64714	siABH4#1 5nM down vs siC 5nM
LOC102724612	-1.64714	siABH4#1 5nM down vs siC 5nM
MIR519D	-1.64689	siABH4#1 5nM down vs siC 5nM
YARS2	-1.64672	siABH4#1 5nM down vs siC 5nM
NT5C2	-1.64662	siABH4#1 5nM down vs siC 5nM
CHML	-1.64626	siABH4#1 5nM down vs siC 5nM
WISP1	-1.64618	siABH4#1 5nM down vs siC 5nM
CASC23	-1.64595	siABH4#1 5nM down vs siC 5nM
VKORC1L1	-1.64569	siABH4#1 5nM down vs siC 5nM
GOLGA8H	-1.64566	siABH4#1 5nM down vs siC 5nM
SAP30	-1.64505	siABH4#1 5nM down vs siC 5nM
SGCB	-1.64494	siABH4#1 5nM down vs siC 5nM
NCKAP5	-1.64491	siABH4#1 5nM down vs siC 5nM
MIR1912	-1.64465	siABH4#1 5nM down vs siC 5nM
DDX12P	-1.6446	siABH4#1 5nM down vs siC 5nM

POLE3	-1.64429	siABH4#1 5nM down vs siC 5nM
PRAC1	-1.64423	siABH4#1 5nM down vs siC 5nM
NAE1	-1.64334	siABH4#1 5nM down vs siC 5nM
GPR3	-1.6428	siABH4#1 5nM down vs siC 5nM
KC6	-1.64229	siABH4#1 5nM down vs siC 5nM
SMIM21	-1.64217	siABH4#1 5nM down vs siC 5nM
USP37	-1.64209	siABH4#1 5nM down vs siC 5nM
C1orf131	-1.64172	siABH4#1 5nM down vs siC 5nM
LOC728026	-1.64171	siABH4#1 5nM down vs siC 5nM
FAIM	-1.64165	siABH4#1 5nM down vs siC 5nM
GTF2H2B	-1.64159	siABH4#1 5nM down vs siC 5nM
POP7	-1.64141	siABH4#1 5nM down vs siC 5nM
LOC105370232	-1.6414	siABH4#1 5nM down vs siC 5nM
LOC105370495	-1.64137	siABH4#1 5nM down vs siC 5nM
CEP57L1	-1.64048	siABH4#1 5nM down vs siC 5nM
MIR548D1	-1.64036	siABH4#1 5nM down vs siC 5nM
POM121L4P	-1.64031	siABH4#1 5nM down vs siC 5nM
IQCF5-AS1	-1.64007	siABH4#1 5nM down vs siC 5nM
AIMP2	-1.63998	siABH4#1 5nM down vs siC 5nM
GPRC5B	-1.63992	siABH4#1 5nM down vs siC 5nM
CHEK2	-1.63972	siABH4#1 5nM down vs siC 5nM
PRDM1	-1.63926	siABH4#1 5nM down vs siC 5nM
NAA60	-1.63924	siABH4#1 5nM down vs siC 5nM
LOC105373953	-1.63891	siABH4#1 5nM down vs siC 5nM
BUB3	-1.63836	siABH4#1 5nM down vs siC 5nM
MAGOHB	-1.63821	siABH4#1 5nM down vs siC 5nM
ZNF180	-1.63806	siABH4#1 5nM down vs siC 5nM
TAF4B	-1.63729	siABH4#1 5nM down vs siC 5nM
LOC105374057	-1.63697	siABH4#1 5nM down vs siC 5nM
LOC150051	-1.63645	siABH4#1 5nM down vs siC 5nM
SCARNA11	-1.63589	siABH4#1 5nM down vs siC 5nM
HSPA9	-1.63579	siABH4#1 5nM down vs siC 5nM

MBD4	-1.6354	siABH4#1 5nM down vs siC 5nM
XPO7	-1.63512	siABH4#1 5nM down vs siC 5nM
ISY1	-1.63507	siABH4#1 5nM down vs siC 5nM
SNHG19	-1.63464	siABH4#1 5nM down vs siC 5nM
RPS13	-1.63461	siABH4#1 5nM down vs siC 5nM
GCLM	-1.63431	siABH4#1 5nM down vs siC 5nM
NACA2	-1.63401	siABH4#1 5nM down vs siC 5nM
TUBB	-1.63369	siABH4#1 5nM down vs siC 5nM
SLC12A2	-1.63313	siABH4#1 5nM down vs siC 5nM
NPRL3	-1.63282	siABH4#1 5nM down vs siC 5nM
SRSF1	-1.63275	siABH4#1 5nM down vs siC 5nM
ZNHIT6	-1.63252	siABH4#1 5nM down vs siC 5nM
RNGTT	-1.63232	siABH4#1 5nM down vs siC 5nM
HADHA	-1.63213	siABH4#1 5nM down vs siC 5nM
PSMD12	-1.63171	siABH4#1 5nM down vs siC 5nM
ST6GAL2	-1.63109	siABH4#1 5nM down vs siC 5nM
DLL3	-1.63095	siABH4#1 5nM down vs siC 5nM
RPSA	-1.63091	siABH4#1 5nM down vs siC 5nM
PSMA3	-1.63081	siABH4#1 5nM down vs siC 5nM
DCPS	-1.63044	siABH4#1 5nM down vs siC 5nM
XRCC6BP1	-1.63017	siABH4#1 5nM down vs siC 5nM
LINC01603	-1.62998	siABH4#1 5nM down vs siC 5nM
MAATS1	-1.62989	siABH4#1 5nM down vs siC 5nM
ZNF562	-1.62983	siABH4#1 5nM down vs siC 5nM
HNRNPA1	-1.62891	siABH4#1 5nM down vs siC 5nM
TVP23B	-1.62874	siABH4#1 5nM down vs siC 5nM
HPS5	-1.62831	siABH4#1 5nM down vs siC 5nM
CA5BP1	-1.62801	siABH4#1 5nM down vs siC 5nM
CPSF3	-1.62797	siABH4#1 5nM down vs siC 5nM
APOC1	-1.62765	siABH4#1 5nM down vs siC 5nM
KPNB1	-1.62744	siABH4#1 5nM down vs siC 5nM
SCGB3A2	-1.62729	siABH4#1 5nM down vs siC 5nM

CHRNA5	-1.62718	siABH4#1 5nM down vs siC 5nM
FFAR4	-1.62708	siABH4#1 5nM down vs siC 5nM
CYCS	-1.62691	siABH4#1 5nM down vs siC 5nM
GMCL1	-1.6268	siABH4#1 5nM down vs siC 5nM
PTGES3	-1.62641	siABH4#1 5nM down vs siC 5nM
C16orf59	-1.6253	siABH4#1 5nM down vs siC 5nM
CCT5	-1.62526	siABH4#1 5nM down vs siC 5nM
MIR542	-1.62521	siABH4#1 5nM down vs siC 5nM
LOC101928324	-1.62521	siABH4#1 5nM down vs siC 5nM
LOC101929492	-1.62501	siABH4#1 5nM down vs siC 5nM
LOC101927993	-1.62458	siABH4#1 5nM down vs siC 5nM
TSR2	-1.62442	siABH4#1 5nM down vs siC 5nM
TNRC6A	-1.62398	siABH4#1 5nM down vs siC 5nM
MIR17HG	-1.62378	siABH4#1 5nM down vs siC 5nM
ADAMTS16	-1.62342	siABH4#1 5nM down vs siC 5nM
KCTD3	-1.62293	siABH4#1 5nM down vs siC 5nM
FUS	-1.62278	siABH4#1 5nM down vs siC 5nM
WDR36	-1.62246	siABH4#1 5nM down vs siC 5nM
COX17	-1.6222	siABH4#1 5nM down vs siC 5nM
STC1	-1.62219	siABH4#1 5nM down vs siC 5nM
NXT1	-1.62212	siABH4#1 5nM down vs siC 5nM
MIRLET7A2	-1.62198	siABH4#1 5nM down vs siC 5nM
VPS9D1-AS1	-1.62181	siABH4#1 5nM down vs siC 5nM
CWC22	-1.621	siABH4#1 5nM down vs siC 5nM
SDCBPP2	-1.62077	siABH4#1 5nM down vs siC 5nM
SNORD102	-1.62022	siABH4#1 5nM down vs siC 5nM
LYZL1	-1.62016	siABH4#1 5nM down vs siC 5nM
TSPAN11	-1.6201	siABH4#1 5nM down vs siC 5nM
CLCN3	-1.62007	siABH4#1 5nM down vs siC 5nM
RPS6KA5	-1.61996	siABH4#1 5nM down vs siC 5nM
GLMN	-1.61954	siABH4#1 5nM down vs siC 5nM
FASTKD3	-1.61916	siABH4#1 5nM down vs siC 5nM

LOC105371277	-1.61901	siABH4#1 5nM down vs siC 5nM
RBM28	-1.61879	siABH4#1 5nM down vs siC 5nM
LOC101929268	-1.61819	siABH4#1 5nM down vs siC 5nM
C1QBP	-1.61795	siABH4#1 5nM down vs siC 5nM
TADA2A	-1.61778	siABH4#1 5nM down vs siC 5nM
LOC105375163	-1.61711	siABH4#1 5nM down vs siC 5nM
SMC1A	-1.61706	siABH4#1 5nM down vs siC 5nM
SNORA2B	-1.61675	siABH4#1 5nM down vs siC 5nM
LOC728554	-1.61611	siABH4#1 5nM down vs siC 5nM
MAGOH	-1.61509	siABH4#1 5nM down vs siC 5nM
ZDHHC13	-1.61456	siABH4#1 5nM down vs siC 5nM
TTI1	-1.61413	siABH4#1 5nM down vs siC 5nM
LSM3	-1.61368	siABH4#1 5nM down vs siC 5nM
LINC00857	-1.61364	siABH4#1 5nM down vs siC 5nM
GRAMD1B	-1.61361	siABH4#1 5nM down vs siC 5nM
SRSF10	-1.61349	siABH4#1 5nM down vs siC 5nM
CTSC	-1.61315	siABH4#1 5nM down vs siC 5nM
IMP3	-1.61311	siABH4#1 5nM down vs siC 5nM
HMGA1	-1.61283	siABH4#1 5nM down vs siC 5nM
MEIS2	-1.61254	siABH4#1 5nM down vs siC 5nM
BOLA3	-1.61253	siABH4#1 5nM down vs siC 5nM
ARL4A	-1.61253	siABH4#1 5nM down vs siC 5nM
FTSJ2	-1.61133	siABH4#1 5nM down vs siC 5nM
LOC105374900	-1.61128	siABH4#1 5nM down vs siC 5nM
RNVU1-6	-1.61103	siABH4#1 5nM down vs siC 5nM
CHRAC1	-1.61056	siABH4#1 5nM down vs siC 5nM
METAP2	-1.61038	siABH4#1 5nM down vs siC 5nM
BCCIP	-1.60959	siABH4#1 5nM down vs siC 5nM
RRS1	-1.6087	siABH4#1 5nM down vs siC 5nM
MIR4697HG	-1.60866	siABH4#1 5nM down vs siC 5nM
SNRNP48	-1.60849	siABH4#1 5nM down vs siC 5nM
ZNF614	-1.60837	siABH4#1 5nM down vs siC 5nM

FUBP1	-1.60832	siABH4#1 5nM down vs siC 5nM
RBM27	-1.6083	siABH4#1 5nM down vs siC 5nM
SNX5	-1.60816	siABH4#1 5nM down vs siC 5nM
EVI2B	-1.6079	siABH4#1 5nM down vs siC 5nM
FBXO30	-1.60784	siABH4#1 5nM down vs siC 5nM
VPRBP	-1.60765	siABH4#1 5nM down vs siC 5nM
C6orf106	-1.60719	siABH4#1 5nM down vs siC 5nM
DCTPP1	-1.60671	siABH4#1 5nM down vs siC 5nM
ING3	-1.60667	siABH4#1 5nM down vs siC 5nM
RPAIN	-1.60655	siABH4#1 5nM down vs siC 5nM
EXOSC2	-1.60638	siABH4#1 5nM down vs siC 5nM
SAFB	-1.60612	siABH4#1 5nM down vs siC 5nM
MIR451B	-1.6059	siABH4#1 5nM down vs siC 5nM
SALL1	-1.60536	siABH4#1 5nM down vs siC 5nM
IGFLR1	-1.60522	siABH4#1 5nM down vs siC 5nM
LOC102723627	-1.60493	siABH4#1 5nM down vs siC 5nM
LOC105369455	-1.60474	siABH4#1 5nM down vs siC 5nM
INAFM2	-1.60474	siABH4#1 5nM down vs siC 5nM
HNRNPAB	-1.60407	siABH4#1 5nM down vs siC 5nM
POC5	-1.60402	siABH4#1 5nM down vs siC 5nM
FSTL4	-1.60378	siABH4#1 5nM down vs siC 5nM
NACAP1	-1.60334	siABH4#1 5nM down vs siC 5nM
NFE2L3	-1.60329	siABH4#1 5nM down vs siC 5nM
CEBPG	-1.60299	siABH4#1 5nM down vs siC 5nM
IGLV5-45	-1.60247	siABH4#1 5nM down vs siC 5nM
NUDT1	-1.60234	siABH4#1 5nM down vs siC 5nM
LOC105372133	-1.60218	siABH4#1 5nM down vs siC 5nM
LOC105378120	-1.60213	siABH4#1 5nM down vs siC 5nM
SLC20A1	-1.60206	siABH4#1 5nM down vs siC 5nM
ZNF488	-1.60178	siABH4#1 5nM down vs siC 5nM
PBX3	-1.60164	siABH4#1 5nM down vs siC 5nM
CYP3A7	-1.60153	siABH4#1 5nM down vs siC 5nM

FAM81A	-1.59979	siABH4#1 5nM down vs siC 5nM
TOMM6	-1.59936	siABH4#1 5nM down vs siC 5nM
ZBED9	-1.59926	siABH4#1 5nM down vs siC 5nM
CA2	-1.59914	siABH4#1 5nM down vs siC 5nM
CPT1A	-1.59848	siABH4#1 5nM down vs siC 5nM
FAM43A	-1.59844	siABH4#1 5nM down vs siC 5nM
LOC646903	-1.59826	siABH4#1 5nM down vs siC 5nM
LL22NC03-86D4.1	-1.59811	siABH4#1 5nM down vs siC 5nM
GEMIN2	-1.59793	siABH4#1 5nM down vs siC 5nM
DDX20	-1.59789	siABH4#1 5nM down vs siC 5nM
PAICS	-1.5976	siABH4#1 5nM down vs siC 5nM
CEP72	-1.59697	siABH4#1 5nM down vs siC 5nM
MIR3941	-1.59695	siABH4#1 5nM down vs siC 5nM
RARG	-1.59687	siABH4#1 5nM down vs siC 5nM
KCTD9	-1.59655	siABH4#1 5nM down vs siC 5nM
ARHGEF5	-1.5965	siABH4#1 5nM down vs siC 5nM
SHMT2	-1.5965	siABH4#1 5nM down vs siC 5nM
CLDN7	-1.59649	siABH4#1 5nM down vs siC 5nM
PCID2	-1.59632	siABH4#1 5nM down vs siC 5nM
RIMKLA	-1.59622	siABH4#1 5nM down vs siC 5nM
MIR450A2	-1.59613	siABH4#1 5nM down vs siC 5nM
PMP2	-1.59598	siABH4#1 5nM down vs siC 5nM
DHX40	-1.59591	siABH4#1 5nM down vs siC 5nM
CHORDC1	-1.59583	siABH4#1 5nM down vs siC 5nM
HPS3	-1.59582	siABH4#1 5nM down vs siC 5nM
RNU6-82P	-1.59577	siABH4#1 5nM down vs siC 5nM
LOC100996288	-1.59577	siABH4#1 5nM down vs siC 5nM
LOC105369582	-1.59557	siABH4#1 5nM down vs siC 5nM
CEP162	-1.59557	siABH4#1 5nM down vs siC 5nM
TSEN15	-1.59544	siABH4#1 5nM down vs siC 5nM
TIMM22	-1.59538	siABH4#1 5nM down vs siC 5nM
LOC105375570	-1.59511	siABH4#1 5nM down vs siC 5nM

TBC1D15	-1.59502	siABH4#1 5nM down vs siC 5nM
DLAT	-1.59466	siABH4#1 5nM down vs siC 5nM
PMS2	-1.59453	siABH4#1 5nM down vs siC 5nM
KIF5B	-1.59448	siABH4#1 5nM down vs siC 5nM
MIR3684	-1.59329	siABH4#1 5nM down vs siC 5nM
MIR1302-6	-1.59299	siABH4#1 5nM down vs siC 5nM
RPL10A	-1.5928	siABH4#1 5nM down vs siC 5nM
RNASE3	-1.59255	siABH4#1 5nM down vs siC 5nM
LOC105375566	-1.5923	siABH4#1 5nM down vs siC 5nM
ZNF331	-1.59209	siABH4#1 5nM down vs siC 5nM
SPRNP1	-1.59166	siABH4#1 5nM down vs siC 5nM
CPSF2	-1.59108	siABH4#1 5nM down vs siC 5nM
EYA3	-1.5908	siABH4#1 5nM down vs siC 5nM
LOC105373377	-1.59071	siABH4#1 5nM down vs siC 5nM
SACM1L	-1.59034	siABH4#1 5nM down vs siC 5nM
PHGR1	-1.59	siABH4#1 5nM down vs siC 5nM
MIR3148	-1.58992	siABH4#1 5nM down vs siC 5nM
FAM83C-AS1	-1.58943	siABH4#1 5nM down vs siC 5nM
USP10	-1.58901	siABH4#1 5nM down vs siC 5nM
ME2	-1.589	siABH4#1 5nM down vs siC 5nM
PPARG	-1.5889	siABH4#1 5nM down vs siC 5nM
L2HGDH	-1.58865	siABH4#1 5nM down vs siC 5nM
WDR35	-1.58783	siABH4#1 5nM down vs siC 5nM
REEP2	-1.5876	siABH4#1 5nM down vs siC 5nM
NUFIP1	-1.58744	siABH4#1 5nM down vs siC 5nM
FAM133A	-1.58713	siABH4#1 5nM down vs siC 5nM
NADK	-1.58691	siABH4#1 5nM down vs siC 5nM
PIK3CB	-1.58685	siABH4#1 5nM down vs siC 5nM
LOC100506248	-1.58666	siABH4#1 5nM down vs siC 5nM
LOC101928819	-1.58654	siABH4#1 5nM down vs siC 5nM
RHEBL1	-1.58653	siABH4#1 5nM down vs siC 5nM
MIR4529	-1.58632	siABH4#1 5nM down vs siC 5nM

MIR520C	-1.58621	siABH4#1 5nM down vs siC 5nM
G3BP2	-1.58611	siABH4#1 5nM down vs siC 5nM
HAUS2	-1.58561	siABH4#1 5nM down vs siC 5nM
PARL	-1.58559	siABH4#1 5nM down vs siC 5nM
DIS3L	-1.58534	siABH4#1 5nM down vs siC 5nM
GATAD2A	-1.58532	siABH4#1 5nM down vs siC 5nM
ATG9A	-1.58498	siABH4#1 5nM down vs siC 5nM
CD300LB	-1.58497	siABH4#1 5nM down vs siC 5nM
LOC102725021	-1.58461	siABH4#1 5nM down vs siC 5nM
MIR3134	-1.5844	siABH4#1 5nM down vs siC 5nM
RASSF5	-1.58379	siABH4#1 5nM down vs siC 5nM
FKRP	-1.58379	siABH4#1 5nM down vs siC 5nM
STK4	-1.58331	siABH4#1 5nM down vs siC 5nM
MAK16	-1.58314	siABH4#1 5nM down vs siC 5nM
IFITM1	-1.5825	siABH4#1 5nM down vs siC 5nM
DHCR7	-1.58249	siABH4#1 5nM down vs siC 5nM
TXNDC12	-1.58244	siABH4#1 5nM down vs siC 5nM
KRT78	-1.58242	siABH4#1 5nM down vs siC 5nM
CST9LP1	-1.58218	siABH4#1 5nM down vs siC 5nM
SUSD2	-1.58211	siABH4#1 5nM down vs siC 5nM
CMC2	-1.58152	siABH4#1 5nM down vs siC 5nM
RNF5P1	-1.58132	siABH4#1 5nM down vs siC 5nM
LRP3	-1.58077	siABH4#1 5nM down vs siC 5nM
LOC401585	-1.58018	siABH4#1 5nM down vs siC 5nM
CCDC15	-1.58016	siABH4#1 5nM down vs siC 5nM
OR2A25	-1.58007	siABH4#1 5nM down vs siC 5nM
TOMM22	-1.57968	siABH4#1 5nM down vs siC 5nM
DPYD	-1.57903	siABH4#1 5nM down vs siC 5nM
LXN	-1.57893	siABH4#1 5nM down vs siC 5nM
CLPB	-1.57851	siABH4#1 5nM down vs siC 5nM
NCBP3	-1.57831	siABH4#1 5nM down vs siC 5nM
E2F6	-1.57785	siABH4#1 5nM down vs siC 5nM

NUDCD2	-1.57756	siABH4#1 5nM down vs siC 5nM
LOC101929976	-1.57731	siABH4#1 5nM down vs siC 5nM
CEP85	-1.5773	siABH4#1 5nM down vs siC 5nM
CCT6P1	-1.57722	siABH4#1 5nM down vs siC 5nM
NUP98	-1.57709	siABH4#1 5nM down vs siC 5nM
ZNF525	-1.57707	siABH4#1 5nM down vs siC 5nM
MIR610	-1.57696	siABH4#1 5nM down vs siC 5nM
ZSCAN12	-1.57695	siABH4#1 5nM down vs siC 5nM
SUMO2	-1.57653	siABH4#1 5nM down vs siC 5nM
TIMP4	-1.57635	siABH4#1 5nM down vs siC 5nM
ZCCHC8	-1.57617	siABH4#1 5nM down vs siC 5nM
LOC105376439	-1.576	siABH4#1 5nM down vs siC 5nM
RPL21	-1.57599	siABH4#1 5nM down vs siC 5nM
CDKN1B	-1.57594	siABH4#1 5nM down vs siC 5nM
OPA1	-1.57514	siABH4#1 5nM down vs siC 5nM
ANKRD36B	-1.57472	siABH4#1 5nM down vs siC 5nM
FAM47C	-1.57469	siABH4#1 5nM down vs siC 5nM
PDGFRL	-1.57459	siABH4#1 5nM down vs siC 5nM
THY1	-1.5745	siABH4#1 5nM down vs siC 5nM
CARHSP1	-1.5742	siABH4#1 5nM down vs siC 5nM
PNN	-1.57406	siABH4#1 5nM down vs siC 5nM
KRTAP4-7	-1.5739	siABH4#1 5nM down vs siC 5nM
PSMA5	-1.57367	siABH4#1 5nM down vs siC 5nM
FAM85A	-1.57337	siABH4#1 5nM down vs siC 5nM
C11orf24	-1.57231	siABH4#1 5nM down vs siC 5nM
DDX39A	-1.57186	siABH4#1 5nM down vs siC 5nM
EYA4	-1.57163	siABH4#1 5nM down vs siC 5nM
TMCC1-AS1	-1.57161	siABH4#1 5nM down vs siC 5nM
AGPS	-1.57156	siABH4#1 5nM down vs siC 5nM
DNAJC1	-1.57152	siABH4#1 5nM down vs siC 5nM
UBE4B	-1.57127	siABH4#1 5nM down vs siC 5nM
RPS9	-1.57124	siABH4#1 5nM down vs siC 5nM

SNRPB	-1.57096	siABH4#1 5nM down vs siC 5nM
CNTNAP3B	-1.57062	siABH4#1 5nM down vs siC 5nM
THOC2	-1.57013	siABH4#1 5nM down vs siC 5nM
TTLL7	-1.56992	siABH4#1 5nM down vs siC 5nM
IGHV3OR16-12	-1.56972	siABH4#1 5nM down vs siC 5nM
HIST3H2A	-1.56919	siABH4#1 5nM down vs siC 5nM
EIF2S2	-1.56902	siABH4#1 5nM down vs siC 5nM
SPANXC	-1.56874	siABH4#1 5nM down vs siC 5nM
ZNF551	-1.56854	siABH4#1 5nM down vs siC 5nM
TOMM40	-1.56853	siABH4#1 5nM down vs siC 5nM
STAG3L2	-1.56824	siABH4#1 5nM down vs siC 5nM
GLYATL2	-1.56822	siABH4#1 5nM down vs siC 5nM
PTDSS1	-1.56817	siABH4#1 5nM down vs siC 5nM
USP14	-1.56797	siABH4#1 5nM down vs siC 5nM
APOH	-1.56724	siABH4#1 5nM down vs siC 5nM
EIF3L	-1.5669	siABH4#1 5nM down vs siC 5nM
PDZRN4	-1.56636	siABH4#1 5nM down vs siC 5nM
ANKRD30B	-1.56636	siABH4#1 5nM down vs siC 5nM
TMUB1	-1.56633	siABH4#1 5nM down vs siC 5nM
RASSF1	-1.56625	siABH4#1 5nM down vs siC 5nM
UTP18	-1.56604	siABH4#1 5nM down vs siC 5nM
PALB2	-1.56549	siABH4#1 5nM down vs siC 5nM
DHX9	-1.56527	siABH4#1 5nM down vs siC 5nM
LOC105377502	-1.56516	siABH4#1 5nM down vs siC 5nM
RNU6-59P	-1.56504	siABH4#1 5nM down vs siC 5nM
DDX55	-1.56474	siABH4#1 5nM down vs siC 5nM
NCL	-1.56473	siABH4#1 5nM down vs siC 5nM
GDI2	-1.56455	siABH4#1 5nM down vs siC 5nM
OSTC	-1.56453	siABH4#1 5nM down vs siC 5nM
FLVCR1-AS1	-1.56446	siABH4#1 5nM down vs siC 5nM
MDC1	-1.56422	siABH4#1 5nM down vs siC 5nM
ALKBH2	-1.56397	siABH4#1 5nM down vs siC 5nM

MIR573	-1.56373	siABH4#1 5nM down vs siC 5nM
LOC105376472	-1.56333	siABH4#1 5nM down vs siC 5nM
FANCC	-1.56328	siABH4#1 5nM down vs siC 5nM
LOC105370058	-1.56323	siABH4#1 5nM down vs siC 5nM
FBXL3	-1.56306	siABH4#1 5nM down vs siC 5nM
LOC105378376	-1.56291	siABH4#1 5nM down vs siC 5nM
MRPS12	-1.56199	siABH4#1 5nM down vs siC 5nM
LINC00862	-1.56113	siABH4#1 5nM down vs siC 5nM
PARP1	-1.56099	siABH4#1 5nM down vs siC 5nM
LIAS	-1.56085	siABH4#1 5nM down vs siC 5nM
PRKAA1	-1.56071	siABH4#1 5nM down vs siC 5nM
TOPORS	-1.56038	siABH4#1 5nM down vs siC 5nM
LOC105370878	-1.55999	siABH4#1 5nM down vs siC 5nM
C10orf113	-1.5597	siABH4#1 5nM down vs siC 5nM
SNORA33	-1.55926	siABH4#1 5nM down vs siC 5nM
RDX	-1.55924	siABH4#1 5nM down vs siC 5nM
COMMD10	-1.55906	siABH4#1 5nM down vs siC 5nM
TTL	-1.55883	siABH4#1 5nM down vs siC 5nM
MAP6	-1.55878	siABH4#1 5nM down vs siC 5nM
ACYP1	-1.55854	siABH4#1 5nM down vs siC 5nM
BYSL	-1.55826	siABH4#1 5nM down vs siC 5nM
CLASP2	-1.55795	siABH4#1 5nM down vs siC 5nM
RNFT2	-1.55751	siABH4#1 5nM down vs siC 5nM
CHUK	-1.5573	siABH4#1 5nM down vs siC 5nM
LOC101928847	-1.5566	siABH4#1 5nM down vs siC 5nM
DHRS11	-1.55655	siABH4#1 5nM down vs siC 5nM
MIR3975	-1.55626	siABH4#1 5nM down vs siC 5nM
ANXA1	-1.55616	siABH4#1 5nM down vs siC 5nM
OR56A3	-1.55611	siABH4#1 5nM down vs siC 5nM
TAF1D	-1.55607	siABH4#1 5nM down vs siC 5nM
TNFRSF21	-1.55598	siABH4#1 5nM down vs siC 5nM
INIP	-1.55584	siABH4#1 5nM down vs siC 5nM

PIGR	-1.55559	siABH4#1 5nM down vs siC 5nM
LOC101929132	-1.55551	siABH4#1 5nM down vs siC 5nM
SMARCC1	-1.5555	siABH4#1 5nM down vs siC 5nM
SNRPD3	-1.55498	siABH4#1 5nM down vs siC 5nM
COIL	-1.55496	siABH4#1 5nM down vs siC 5nM
SNHG8	-1.55472	siABH4#1 5nM down vs siC 5nM
MAP2K1	-1.5546	siABH4#1 5nM down vs siC 5nM
ACOT12	-1.55443	siABH4#1 5nM down vs siC 5nM
KIAA1429	-1.55439	siABH4#1 5nM down vs siC 5nM
ANKRD27	-1.55431	siABH4#1 5nM down vs siC 5nM
ERICH1	-1.55419	siABH4#1 5nM down vs siC 5nM
RSL1D1	-1.55418	siABH4#1 5nM down vs siC 5nM
LOC105378930	-1.55414	siABH4#1 5nM down vs siC 5nM
HSPA8	-1.55383	siABH4#1 5nM down vs siC 5nM
PAXIP1	-1.55357	siABH4#1 5nM down vs siC 5nM
EDC3	-1.55353	siABH4#1 5nM down vs siC 5nM
C15orf61	-1.55346	siABH4#1 5nM down vs siC 5nM
LOC105369941	-1.55324	siABH4#1 5nM down vs siC 5nM
HAS2-AS1	-1.55306	siABH4#1 5nM down vs siC 5nM
PTPN2	-1.55282	siABH4#1 5nM down vs siC 5nM
TBC1D14	-1.55256	siABH4#1 5nM down vs siC 5nM
FLVCR2	-1.55196	siABH4#1 5nM down vs siC 5nM
HILPDA	-1.55192	siABH4#1 5nM down vs siC 5nM
LOC105370822	-1.5517	siABH4#1 5nM down vs siC 5nM
PRDX4	-1.55153	siABH4#1 5nM down vs siC 5nM
HACD3	-1.55124	siABH4#1 5nM down vs siC 5nM
SCLT1	-1.55121	siABH4#1 5nM down vs siC 5nM
HIF1A-AS2	-1.55086	siABH4#1 5nM down vs siC 5nM
PCF11	-1.55058	siABH4#1 5nM down vs siC 5nM
SNORA66	-1.55024	siABH4#1 5nM down vs siC 5nM
LOC101929109	-1.55018	siABH4#1 5nM down vs siC 5nM
CARD8-AS1	-1.55005	siABH4#1 5nM down vs siC 5nM

MT1CP	-1.54993	siABH4#1 5nM down vs siC 5nM
RNY4P13	-1.54918	siABH4#1 5nM down vs siC 5nM
RBBP6	-1.54862	siABH4#1 5nM down vs siC 5nM
TUBB4B	-1.54849	siABH4#1 5nM down vs siC 5nM
ZNF280B	-1.54827	siABH4#1 5nM down vs siC 5nM
SOCS4	-1.54803	siABH4#1 5nM down vs siC 5nM
SCARNA8	-1.54784	siABH4#1 5nM down vs siC 5nM
ZNF362	-1.54769	siABH4#1 5nM down vs siC 5nM
MYC	-1.54758	siABH4#1 5nM down vs siC 5nM
LDLRAD3	-1.54754	siABH4#1 5nM down vs siC 5nM
DUBR	-1.54711	siABH4#1 5nM down vs siC 5nM
XRCC1	-1.54704	siABH4#1 5nM down vs siC 5nM
IFIT5	-1.54696	siABH4#1 5nM down vs siC 5nM
LAS1L	-1.54684	siABH4#1 5nM down vs siC 5nM
NOC3L	-1.54684	siABH4#1 5nM down vs siC 5nM
KIAA0895	-1.54661	siABH4#1 5nM down vs siC 5nM
LOC101929372	-1.54627	siABH4#1 5nM down vs siC 5nM
USP16	-1.54612	siABH4#1 5nM down vs siC 5nM
TEX28	-1.54604	siABH4#1 5nM down vs siC 5nM
SNORA14B	-1.54603	siABH4#1 5nM down vs siC 5nM
COPS2	-1.54599	siABH4#1 5nM down vs siC 5nM
DUS3L	-1.54576	siABH4#1 5nM down vs siC 5nM
CACYBP	-1.54571	siABH4#1 5nM down vs siC 5nM
LOC105377175	-1.54564	siABH4#1 5nM down vs siC 5nM
XK	-1.54553	siABH4#1 5nM down vs siC 5nM
MIR525	-1.54499	siABH4#1 5nM down vs siC 5nM
SIVA1	-1.54464	siABH4#1 5nM down vs siC 5nM
ZNF106	-1.54459	siABH4#1 5nM down vs siC 5nM
MYB	-1.54453	siABH4#1 5nM down vs siC 5nM
LOC105376642	-1.54445	siABH4#1 5nM down vs siC 5nM
SLC25A22	-1.54445	siABH4#1 5nM down vs siC 5nM
IGFBP1	-1.5444	siABH4#1 5nM down vs siC 5nM

EIF5A2	-1.54427	siABH4#1 5nM down vs siC 5nM
LHX2	-1.54421	siABH4#1 5nM down vs siC 5nM
MLX	-1.54403	siABH4#1 5nM down vs siC 5nM
LOC105372615	-1.54394	siABH4#1 5nM down vs siC 5nM
SIX4	-1.54332	siABH4#1 5nM down vs siC 5nM
NEIL2	-1.54314	siABH4#1 5nM down vs siC 5nM
USP21	-1.54287	siABH4#1 5nM down vs siC 5nM
LOC93622	-1.54281	siABH4#1 5nM down vs siC 5nM
ACP2	-1.54252	siABH4#1 5nM down vs siC 5nM
C15orf39	-1.5424	siABH4#1 5nM down vs siC 5nM
FBXO21	-1.54209	siABH4#1 5nM down vs siC 5nM
SMCR8	-1.54182	siABH4#1 5nM down vs siC 5nM
NR2F2	-1.54181	siABH4#1 5nM down vs siC 5nM
SLC25A51P1	-1.54152	siABH4#1 5nM down vs siC 5nM
PIGK	-1.54114	siABH4#1 5nM down vs siC 5nM
LINC01098	-1.54108	siABH4#1 5nM down vs siC 5nM
C19orf44	-1.54085	siABH4#1 5nM down vs siC 5nM
MIR509-3	-1.54008	siABH4#1 5nM down vs siC 5nM
LINC01592	-1.54006	siABH4#1 5nM down vs siC 5nM
PTMS	-1.5399	siABH4#1 5nM down vs siC 5nM
DOCK11	-1.53968	siABH4#1 5nM down vs siC 5nM
RPIA	-1.53964	siABH4#1 5nM down vs siC 5nM
TATDN2	-1.53959	siABH4#1 5nM down vs siC 5nM
EFHC1	-1.53957	siABH4#1 5nM down vs siC 5nM
ING2	-1.53951	siABH4#1 5nM down vs siC 5nM
LOC102725179	-1.53918	siABH4#1 5nM down vs siC 5nM
SPATA2	-1.53915	siABH4#1 5nM down vs siC 5nM
CCDC14	-1.53911	siABH4#1 5nM down vs siC 5nM
RIPK1	-1.53898	siABH4#1 5nM down vs siC 5nM
SNORD18A	-1.53859	siABH4#1 5nM down vs siC 5nM
GRWD1	-1.53859	siABH4#1 5nM down vs siC 5nM
PDS5A	-1.53842	siABH4#1 5nM down vs siC 5nM

EFTUD1P1	-1.53821	siABH4#1 5nM down vs siC 5nM
CFAP58-AS1	-1.53812	siABH4#1 5nM down vs siC 5nM
WDR54	-1.5381	siABH4#1 5nM down vs siC 5nM
RAP1GAP	-1.53807	siABH4#1 5nM down vs siC 5nM
ARID5B	-1.53805	siABH4#1 5nM down vs siC 5nM
MIR1290	-1.53799	siABH4#1 5nM down vs siC 5nM
PRR14L	-1.53785	siABH4#1 5nM down vs siC 5nM
MIR320B2	-1.53769	siABH4#1 5nM down vs siC 5nM
NRGN	-1.53763	siABH4#1 5nM down vs siC 5nM
DDX6	-1.53737	siABH4#1 5nM down vs siC 5nM
SNRPC	-1.53736	siABH4#1 5nM down vs siC 5nM
LOC105374437	-1.53705	siABH4#1 5nM down vs siC 5nM
ZNF532	-1.53683	siABH4#1 5nM down vs siC 5nM
COPS3	-1.53682	siABH4#1 5nM down vs siC 5nM
OLMALINC	-1.5368	siABH4#1 5nM down vs siC 5nM
CSNK1G3	-1.53677	siABH4#1 5nM down vs siC 5nM
SCARNA7	-1.53659	siABH4#1 5nM down vs siC 5nM
CDC5L	-1.53657	siABH4#1 5nM down vs siC 5nM
SPICE1	-1.53654	siABH4#1 5nM down vs siC 5nM
R3HCC1L	-1.53635	siABH4#1 5nM down vs siC 5nM
MIR4774	-1.53625	siABH4#1 5nM down vs siC 5nM
PASK	-1.53622	siABH4#1 5nM down vs siC 5nM
ANKRD28	-1.53622	siABH4#1 5nM down vs siC 5nM
MFSD14B	-1.53614	siABH4#1 5nM down vs siC 5nM
PTBP2	-1.53586	siABH4#1 5nM down vs siC 5nM
RASL11A	-1.53574	siABH4#1 5nM down vs siC 5nM
LRRC30	-1.53574	siABH4#1 5nM down vs siC 5nM
CNNM3	-1.53568	siABH4#1 5nM down vs siC 5nM
LOC102724784	-1.5354	siABH4#1 5nM down vs siC 5nM
URB1	-1.53521	siABH4#1 5nM down vs siC 5nM
MIR550A1	-1.5349	siABH4#1 5nM down vs siC 5nM
TTLL4	-1.53482	siABH4#1 5nM down vs siC 5nM

MIR449A	-1.53432	siABH4#1 5nM down vs siC 5nM
SNORD1B	-1.53429	siABH4#1 5nM down vs siC 5nM
RLF	-1.5339	siABH4#1 5nM down vs siC 5nM
MAG	-1.53351	siABH4#1 5nM down vs siC 5nM
PEG10	-1.5335	siABH4#1 5nM down vs siC 5nM
SZRD1	-1.53333	siABH4#1 5nM down vs siC 5nM
TTI2	-1.53315	siABH4#1 5nM down vs siC 5nM
LOC101929715	-1.53313	siABH4#1 5nM down vs siC 5nM
MIR576	-1.53312	siABH4#1 5nM down vs siC 5nM
SNORD119	-1.53306	siABH4#1 5nM down vs siC 5nM
TRGJP1	-1.53292	siABH4#1 5nM down vs siC 5nM
LGALS1	-1.53283	siABH4#1 5nM down vs siC 5nM
LOC105374261	-1.53259	siABH4#1 5nM down vs siC 5nM
MAP3K4	-1.53241	siABH4#1 5nM down vs siC 5nM
NET1	-1.5323	siABH4#1 5nM down vs siC 5nM
ISG20L2	-1.53212	siABH4#1 5nM down vs siC 5nM
RCC2	-1.53194	siABH4#1 5nM down vs siC 5nM
POLR3H	-1.53163	siABH4#1 5nM down vs siC 5nM
TBC1D5	-1.53119	siABH4#1 5nM down vs siC 5nM
FAM71C	-1.53116	siABH4#1 5nM down vs siC 5nM
RSRC1	-1.53111	siABH4#1 5nM down vs siC 5nM
DDX10	-1.53083	siABH4#1 5nM down vs siC 5nM
FIS1	-1.53078	siABH4#1 5nM down vs siC 5nM
MATR3	-1.5307	siABH4#1 5nM down vs siC 5nM
SRSF2	-1.53059	siABH4#1 5nM down vs siC 5nM
VAPA	-1.53057	siABH4#1 5nM down vs siC 5nM
SET	-1.53036	siABH4#1 5nM down vs siC 5nM
HNRNPD	-1.53027	siABH4#1 5nM down vs siC 5nM
ANGPTL2	-1.53013	siABH4#1 5nM down vs siC 5nM
HNRNPUL1	-1.52978	siABH4#1 5nM down vs siC 5nM
ENO2	-1.5296	siABH4#1 5nM down vs siC 5nM
UGT2A2	-1.52925	siABH4#1 5nM down vs siC 5nM

LINC01546	-1.52907	siABH4#1 5nM down vs siC 5nM
MPP6	-1.52876	siABH4#1 5nM down vs siC 5nM
EMC3	-1.52864	siABH4#1 5nM down vs siC 5nM
FLJ35934	-1.5284	siABH4#1 5nM down vs siC 5nM
WDR5	-1.52839	siABH4#1 5nM down vs siC 5nM
LOC100506083	-1.52813	siABH4#1 5nM down vs siC 5nM
LOC101929574	-1.52772	siABH4#1 5nM down vs siC 5nM
RNF139	-1.52761	siABH4#1 5nM down vs siC 5nM
POT1	-1.52758	siABH4#1 5nM down vs siC 5nM
HN1	-1.52748	siABH4#1 5nM down vs siC 5nM
COMMD4	-1.52743	siABH4#1 5nM down vs siC 5nM
RNF145	-1.52718	siABH4#1 5nM down vs siC 5nM
LINC01029	-1.52698	siABH4#1 5nM down vs siC 5nM
RFX2	-1.52684	siABH4#1 5nM down vs siC 5nM
PGAM5	-1.52674	siABH4#1 5nM down vs siC 5nM
TPGS2	-1.52635	siABH4#1 5nM down vs siC 5nM
LOC101927757	-1.52634	siABH4#1 5nM down vs siC 5nM
RAP1GAP2	-1.52634	siABH4#1 5nM down vs siC 5nM
TMEM97	-1.52618	siABH4#1 5nM down vs siC 5nM
TEX10	-1.52594	siABH4#1 5nM down vs siC 5nM
ZNF300	-1.52585	siABH4#1 5nM down vs siC 5nM
SLC25A12	-1.52559	siABH4#1 5nM down vs siC 5nM
PRRG3	-1.52545	siABH4#1 5nM down vs siC 5nM
MAGEA11	-1.52505	siABH4#1 5nM down vs siC 5nM
GARS	-1.52488	siABH4#1 5nM down vs siC 5nM
OTOL1	-1.52479	siABH4#1 5nM down vs siC 5nM
ZNF28	-1.52462	siABH4#1 5nM down vs siC 5nM
FEM1A	-1.52458	siABH4#1 5nM down vs siC 5nM
LOC105378456	-1.52435	siABH4#1 5nM down vs siC 5nM
TXNDC15	-1.5243	siABH4#1 5nM down vs siC 5nM
LUC7L3	-1.52402	siABH4#1 5nM down vs siC 5nM
LSM2	-1.52387	siABH4#1 5nM down vs siC 5nM

TOR1A	-1.52379	siABH4#1 5nM down vs siC 5nM
AGPAT5	-1.52376	siABH4#1 5nM down vs siC 5nM
RBMX2	-1.52373	siABH4#1 5nM down vs siC 5nM
THG1L	-1.52344	siABH4#1 5nM down vs siC 5nM
XRN2	-1.52311	siABH4#1 5nM down vs siC 5nM
CYP2S1	-1.52302	siABH4#1 5nM down vs siC 5nM
GLO1	-1.52283	siABH4#1 5nM down vs siC 5nM
ZC3HAV1L	-1.52266	siABH4#1 5nM down vs siC 5nM
SMN2	-1.5225	siABH4#1 5nM down vs siC 5nM
TOP1	-1.5225	siABH4#1 5nM down vs siC 5nM
DCTN3	-1.52233	siABH4#1 5nM down vs siC 5nM
LOC101927293	-1.5223	siABH4#1 5nM down vs siC 5nM
NBN	-1.52197	siABH4#1 5nM down vs siC 5nM
SOGA1	-1.52169	siABH4#1 5nM down vs siC 5nM
PER2	-1.52157	siABH4#1 5nM down vs siC 5nM
LOC105369996	-1.52124	siABH4#1 5nM down vs siC 5nM
PHACTR4	-1.52118	siABH4#1 5nM down vs siC 5nM
SEZ6	-1.52106	siABH4#1 5nM down vs siC 5nM
ATG3	-1.52105	siABH4#1 5nM down vs siC 5nM
ANKRD39	-1.52094	siABH4#1 5nM down vs siC 5nM
STAG2	-1.52076	siABH4#1 5nM down vs siC 5nM
MIR3143	-1.52059	siABH4#1 5nM down vs siC 5nM
GSTM2P1	-1.52009	siABH4#1 5nM down vs siC 5nM
DPRX	-1.51997	siABH4#1 5nM down vs siC 5nM
GLTSCR2	-1.5199	siABH4#1 5nM down vs siC 5nM
IPO9	-1.51972	siABH4#1 5nM down vs siC 5nM
KEAP1	-1.51956	siABH4#1 5nM down vs siC 5nM
DNAJC2	-1.51946	siABH4#1 5nM down vs siC 5nM
LOC101928231	-1.51945	siABH4#1 5nM down vs siC 5nM
CEP126	-1.51921	siABH4#1 5nM down vs siC 5nM
MRPS18C	-1.51903	siABH4#1 5nM down vs siC 5nM
SLBP	-1.51886	siABH4#1 5nM down vs siC 5nM

RRM1	-1.51865	siABH4#1 5nM down vs siC 5nM
ODC1	-1.51856	siABH4#1 5nM down vs siC 5nM
LOC102724913	-1.51834	siABH4#1 5nM down vs siC 5nM
SLC25A5	-1.51833	siABH4#1 5nM down vs siC 5nM
USP39	-1.51805	siABH4#1 5nM down vs siC 5nM
SKIV2L2	-1.51796	siABH4#1 5nM down vs siC 5nM
EEF1A1	-1.51791	siABH4#1 5nM down vs siC 5nM
NDUFS5	-1.51785	siABH4#1 5nM down vs siC 5nM
UBR1	-1.51766	siABH4#1 5nM down vs siC 5nM
RAD9B	-1.51765	siABH4#1 5nM down vs siC 5nM
LCLAT1	-1.51758	siABH4#1 5nM down vs siC 5nM
TRBV7-4	-1.5174	siABH4#1 5nM down vs siC 5nM
TPR	-1.51733	siABH4#1 5nM down vs siC 5nM
GALNT13	-1.51708	siABH4#1 5nM down vs siC 5nM
SOX21-AS1	-1.51704	siABH4#1 5nM down vs siC 5nM
GNL3	-1.51704	siABH4#1 5nM down vs siC 5nM
FAM169B	-1.51698	siABH4#1 5nM down vs siC 5nM
HIST1H3E	-1.51697	siABH4#1 5nM down vs siC 5nM
TUBE1	-1.51672	siABH4#1 5nM down vs siC 5nM
DPF1	-1.51653	siABH4#1 5nM down vs siC 5nM
HSP90AA1	-1.51619	siABH4#1 5nM down vs siC 5nM
DEFB115	-1.5158	siABH4#1 5nM down vs siC 5nM
RSC1A1	-1.5157	siABH4#1 5nM down vs siC 5nM
ICE2	-1.51522	siABH4#1 5nM down vs siC 5nM
PCDHB9	-1.51521	siABH4#1 5nM down vs siC 5nM
PEX3	-1.51498	siABH4#1 5nM down vs siC 5nM
ERRFI1	-1.5148	siABH4#1 5nM down vs siC 5nM
CNPY4	-1.51476	siABH4#1 5nM down vs siC 5nM
SSR3	-1.51459	siABH4#1 5nM down vs siC 5nM
SNHG15	-1.51444	siABH4#1 5nM down vs siC 5nM
ARHGAP35	-1.51421	siABH4#1 5nM down vs siC 5nM
CWC25	-1.51409	siABH4#1 5nM down vs siC 5nM

KCNAB2	-1.514	siABH4#1 5nM down vs siC 5nM
PSMG2	-1.51367	siABH4#1 5nM down vs siC 5nM
EIF5B	-1.51364	siABH4#1 5nM down vs siC 5nM
MATN3	-1.51318	siABH4#1 5nM down vs siC 5nM
FAM46A	-1.51316	siABH4#1 5nM down vs siC 5nM
LOC105379829	-1.51316	siABH4#1 5nM down vs siC 5nM
GEMIN5	-1.51308	siABH4#1 5nM down vs siC 5nM
KIAA0319	-1.51298	siABH4#1 5nM down vs siC 5nM
RILPL2	-1.51266	siABH4#1 5nM down vs siC 5nM
PCNA	-1.51214	siABH4#1 5nM down vs siC 5nM
SRSF3	-1.51207	siABH4#1 5nM down vs siC 5nM
LIPE	-1.51207	siABH4#1 5nM down vs siC 5nM
LOC105374374	-1.51183	siABH4#1 5nM down vs siC 5nM
BTN2A2	-1.51171	siABH4#1 5nM down vs siC 5nM
LOC105375904	-1.51171	siABH4#1 5nM down vs siC 5nM
CHCHD4	-1.51169	siABH4#1 5nM down vs siC 5nM
RPH3A	-1.51156	siABH4#1 5nM down vs siC 5nM
HOXA7	-1.51153	siABH4#1 5nM down vs siC 5nM
TOP3A	-1.51141	siABH4#1 5nM down vs siC 5nM
NTMT1	-1.5112	siABH4#1 5nM down vs siC 5nM
SCAI	-1.5111	siABH4#1 5nM down vs siC 5nM
SLC16A12-AS1	-1.51108	siABH4#1 5nM down vs siC 5nM
OAZ1	-1.51107	siABH4#1 5nM down vs siC 5nM
MAGEA12	-1.51097	siABH4#1 5nM down vs siC 5nM
KRTAP20-3	-1.51097	siABH4#1 5nM down vs siC 5nM
MIR4742	-1.51092	siABH4#1 5nM down vs siC 5nM
LOC101928635	-1.51058	siABH4#1 5nM down vs siC 5nM
NMD3	-1.51034	siABH4#1 5nM down vs siC 5nM
LOC105374483	-1.51023	siABH4#1 5nM down vs siC 5nM
MBD3	-1.51014	siABH4#1 5nM down vs siC 5nM
MTHFD1	-1.51007	siABH4#1 5nM down vs siC 5nM
TONSL	-1.50998	siABH4#1 5nM down vs siC 5nM

DIAPH1	-1.50993	siABH4#1 5nM down vs siC 5nM
SNHG16	-1.50962	siABH4#1 5nM down vs siC 5nM
ANAPC4	-1.50952	siABH4#1 5nM down vs siC 5nM
PPP2R4	-1.50939	siABH4#1 5nM down vs siC 5nM
ZDHHC6	-1.50938	siABH4#1 5nM down vs siC 5nM
LOC105369526	-1.50918	siABH4#1 5nM down vs siC 5nM
KIF5C	-1.50911	siABH4#1 5nM down vs siC 5nM
CNTNAP3	-1.50907	siABH4#1 5nM down vs siC 5nM
MIR4494	-1.50894	siABH4#1 5nM down vs siC 5nM
CTDSPL	-1.50892	siABH4#1 5nM down vs siC 5nM
TRMT61B	-1.50889	siABH4#1 5nM down vs siC 5nM
TSEN54	-1.50875	siABH4#1 5nM down vs siC 5nM
ILF3	-1.50856	siABH4#1 5nM down vs siC 5nM
LOC101927934	-1.50852	siABH4#1 5nM down vs siC 5nM
ZC3H15	-1.50846	siABH4#1 5nM down vs siC 5nM
ASF1A	-1.50839	siABH4#1 5nM down vs siC 5nM
SRGN	-1.50835	siABH4#1 5nM down vs siC 5nM
FAM216A	-1.50831	siABH4#1 5nM down vs siC 5nM
TMEM39B	-1.50767	siABH4#1 5nM down vs siC 5nM
RPF1	-1.50763	siABH4#1 5nM down vs siC 5nM
CLPSL2	-1.50754	siABH4#1 5nM down vs siC 5nM
SNORA8	-1.50714	siABH4#1 5nM down vs siC 5nM
LOC105369964	-1.50708	siABH4#1 5nM down vs siC 5nM
IFT122	-1.50694	siABH4#1 5nM down vs siC 5nM
OSBPL10	-1.50652	siABH4#1 5nM down vs siC 5nM
LARP7	-1.50648	siABH4#1 5nM down vs siC 5nM
CTDSP1	-1.50625	siABH4#1 5nM down vs siC 5nM
PLAA	-1.50617	siABH4#1 5nM down vs siC 5nM
HELB	-1.50613	siABH4#1 5nM down vs siC 5nM
LOC105378641	-1.5061	siABH4#1 5nM down vs siC 5nM
MIR518E	-1.50576	siABH4#1 5nM down vs siC 5nM
TMEM126B	-1.5057	siABH4#1 5nM down vs siC 5nM

ZNF518A	-1.50513	siABH4#1 5nM down vs siC 5nM
RPLP0	-1.50494	siABH4#1 5nM down vs siC 5nM
TRGC2	-1.50465	siABH4#1 5nM down vs siC 5nM
TRGV9	-1.50465	siABH4#1 5nM down vs siC 5nM
EDN1	-1.50438	siABH4#1 5nM down vs siC 5nM
NELL1	-1.50432	siABH4#1 5nM down vs siC 5nM
HIST1H2AA	-1.50432	siABH4#1 5nM down vs siC 5nM
PAPD7	-1.50427	siABH4#1 5nM down vs siC 5nM
RNASEH1-AS1	-1.50389	siABH4#1 5nM down vs siC 5nM
SLC22A8	-1.50378	siABH4#1 5nM down vs siC 5nM
LOC101927093	-1.50376	siABH4#1 5nM down vs siC 5nM
CDC23	-1.50366	siABH4#1 5nM down vs siC 5nM
SLC10A7	-1.50358	siABH4#1 5nM down vs siC 5nM
ARHGEF26	-1.50348	siABH4#1 5nM down vs siC 5nM
ARRDC1-AS1	-1.50348	siABH4#1 5nM down vs siC 5nM
LINC01264	-1.50345	siABH4#1 5nM down vs siC 5nM
ARL14	-1.50341	siABH4#1 5nM down vs siC 5nM
MIR767	-1.50316	siABH4#1 5nM down vs siC 5nM
MIR874	-1.50311	siABH4#1 5nM down vs siC 5nM
LOC101928408	-1.50289	siABH4#1 5nM down vs siC 5nM
SNRPA	-1.50219	siABH4#1 5nM down vs siC 5nM
NIPBL-AS1	-1.50186	siABH4#1 5nM down vs siC 5nM
PDE3B	-1.50171	siABH4#1 5nM down vs siC 5nM
LOC101927987	-1.50154	siABH4#1 5nM down vs siC 5nM
MIR126	-1.50154	siABH4#1 5nM down vs siC 5nM
CYCSP52	-1.50152	siABH4#1 5nM down vs siC 5nM
MIR508	-1.50152	siABH4#1 5nM down vs siC 5nM
LINC01451	-1.50145	siABH4#1 5nM down vs siC 5nM
SNORD12C	-1.50134	siABH4#1 5nM down vs siC 5nM
MIR3160-1	-1.50122	siABH4#1 5nM down vs siC 5nM
LOC105373586	-1.50121	siABH4#1 5nM down vs siC 5nM
FSTL5	-1.50092	siABH4#1 5nM down vs siC 5nM

LINC01541	-1.50063	siABH4#1 5nM down vs siC 5nM
ARV1	-1.50062	siABH4#1 5nM down vs siC 5nM
MRPL14	-1.50058	siABH4#1 5nM down vs siC 5nM
NCAPH2	-1.50056	siABH4#1 5nM down vs siC 5nM
ALKBH4	-1.50036	siABH4#1 5nM down vs siC 5nM
PACRGL	-1.50018	siABH4#1 5nM down vs siC 5nM
CD160	-1.50018	siABH4#1 5nM down vs siC 5nM
SPIN4	-1.50017	siABH4#1 5nM down vs siC 5nM
FAM83H	1.50027	siABH4#1 5nM up vs siC 5nM
WHAMM	1.50028	siABH4#1 5nM up vs siC 5nM
TCEB2	1.50056	siABH4#1 5nM up vs siC 5nM
DAB2	1.50079	siABH4#1 5nM up vs siC 5nM
FAM182A	1.50087	siABH4#1 5nM up vs siC 5nM
PBXIP1	1.50101	siABH4#1 5nM up vs siC 5nM
CLEC2A	1.50122	siABH4#1 5nM up vs siC 5nM
LINC01370	1.50122	siABH4#1 5nM up vs siC 5nM
LOC105375734	1.50134	siABH4#1 5nM up vs siC 5nM
TAPT1-AS1	1.50187	siABH4#1 5nM up vs siC 5nM
OR11H2	1.50214	siABH4#1 5nM up vs siC 5nM
CDKL4	1.50214	siABH4#1 5nM up vs siC 5nM
LOC105369727	1.50235	siABH4#1 5nM up vs siC 5nM
DUXAP10	1.50242	siABH4#1 5nM up vs siC 5nM
MYOD1	1.50243	siABH4#1 5nM up vs siC 5nM
NPR2	1.50247	siABH4#1 5nM up vs siC 5nM
RC3H2	1.50268	siABH4#1 5nM up vs siC 5nM
CDK2AP2	1.50318	siABH4#1 5nM up vs siC 5nM
DCAF4	1.50341	siABH4#1 5nM up vs siC 5nM
RNF135	1.50342	siABH4#1 5nM up vs siC 5nM
LOC105373862	1.50351	siABH4#1 5nM up vs siC 5nM
RHBDL2	1.50389	siABH4#1 5nM up vs siC 5nM
ROR1	1.50394	siABH4#1 5nM up vs siC 5nM
LOC102724050	1.50395	siABH4#1 5nM up vs siC 5nM

DLGAP4	1.50405	siABH4#1 5nM up vs siC 5nM
LOC102724078	1.50406	siABH4#1 5nM up vs siC 5nM
FRK	1.50432	siABH4#1 5nM up vs siC 5nM
TMEM234	1.50453	siABH4#1 5nM up vs siC 5nM
KRT7	1.50492	siABH4#1 5nM up vs siC 5nM
FAM89B	1.50494	siABH4#1 5nM up vs siC 5nM
SCARB1	1.5054	siABH4#1 5nM up vs siC 5nM
LOC645752	1.50544	siABH4#1 5nM up vs siC 5nM
MSRA	1.50556	siABH4#1 5nM up vs siC 5nM
TSPAN18	1.50558	siABH4#1 5nM up vs siC 5nM
CFAP70	1.50582	siABH4#1 5nM up vs siC 5nM
ODF3L1	1.50603	siABH4#1 5nM up vs siC 5nM
MIR4309	1.50614	siABH4#1 5nM up vs siC 5nM
NTN5	1.50628	siABH4#1 5nM up vs siC 5nM
CDKL5	1.5063	siABH4#1 5nM up vs siC 5nM
RASA4B	1.50643	siABH4#1 5nM up vs siC 5nM
WAS	1.50645	siABH4#1 5nM up vs siC 5nM
OLFML2A	1.50655	siABH4#1 5nM up vs siC 5nM
NMNAT1	1.50658	siABH4#1 5nM up vs siC 5nM
HSD17B3	1.50669	siABH4#1 5nM up vs siC 5nM
FNDC1	1.50672	siABH4#1 5nM up vs siC 5nM
LINC00606	1.50674	siABH4#1 5nM up vs siC 5nM
TMEM64	1.5069	siABH4#1 5nM up vs siC 5nM
OR56A1	1.50709	siABH4#1 5nM up vs siC 5nM
STAT5A	1.50747	siABH4#1 5nM up vs siC 5nM
SCD5	1.50772	siABH4#1 5nM up vs siC 5nM
CEACAM4	1.50822	siABH4#1 5nM up vs siC 5nM
OR2T35	1.50865	siABH4#1 5nM up vs siC 5nM
FRS2	1.50896	siABH4#1 5nM up vs siC 5nM
SNPH	1.50896	siABH4#1 5nM up vs siC 5nM
TNS4	1.50914	siABH4#1 5nM up vs siC 5nM
WDR78	1.50942	siABH4#1 5nM up vs siC 5nM

TPRG1	1.51003	siABH4#1 5nM up vs siC 5nM
LOC102724580	1.51012	siABH4#1 5nM up vs siC 5nM
PRICKLE4	1.51036	siABH4#1 5nM up vs siC 5nM
ATF3	1.51064	siABH4#1 5nM up vs siC 5nM
LINC00969	1.51098	siABH4#1 5nM up vs siC 5nM
OR2G6	1.51107	siABH4#1 5nM up vs siC 5nM
KANK3	1.51139	siABH4#1 5nM up vs siC 5nM
PYCARD-AS1	1.51145	siABH4#1 5nM up vs siC 5nM
MLPH	1.51158	siABH4#1 5nM up vs siC 5nM
NAV1	1.51179	siABH4#1 5nM up vs siC 5nM
PNPLA2	1.51184	siABH4#1 5nM up vs siC 5nM
MAN2B2	1.51224	siABH4#1 5nM up vs siC 5nM
LOC344887	1.51243	siABH4#1 5nM up vs siC 5nM
GALNT10	1.51263	siABH4#1 5nM up vs siC 5nM
GACAT2	1.51299	siABH4#1 5nM up vs siC 5nM
IGSF8	1.51318	siABH4#1 5nM up vs siC 5nM
LOC105370414	1.5133	siABH4#1 5nM up vs siC 5nM
MIR3118-2	1.51351	siABH4#1 5nM up vs siC 5nM
MIR3118-2	1.51351	siABH4#1 5nM up vs siC 5nM
TSPAN33	1.51352	siABH4#1 5nM up vs siC 5nM
TEAD1	1.51418	siABH4#1 5nM up vs siC 5nM
NAT2	1.51448	siABH4#1 5nM up vs siC 5nM
TTPAL	1.51457	siABH4#1 5nM up vs siC 5nM
SFXN3	1.51464	siABH4#1 5nM up vs siC 5nM
LOC105377261	1.51471	siABH4#1 5nM up vs siC 5nM
GMIP	1.515	siABH4#1 5nM up vs siC 5nM
HES2	1.51514	siABH4#1 5nM up vs siC 5nM
BACH1	1.51572	siABH4#1 5nM up vs siC 5nM
BHLHE41	1.51578	siABH4#1 5nM up vs siC 5nM
RALGPS2	1.51614	siABH4#1 5nM up vs siC 5nM
KLHDC1	1.51643	siABH4#1 5nM up vs siC 5nM
FAH	1.51647	siABH4#1 5nM up vs siC 5nM

BRMS1L	1.51661	siABH4#1 5nM up vs siC 5nM
LINC01269	1.51671	siABH4#1 5nM up vs siC 5nM
TAP1	1.51673	siABH4#1 5nM up vs siC 5nM
MIR181A1HG	1.51679	siABH4#1 5nM up vs siC 5nM
CLYBL	1.51682	siABH4#1 5nM up vs siC 5nM
PRAF2	1.51723	siABH4#1 5nM up vs siC 5nM
NPIPB15	1.51727	siABH4#1 5nM up vs siC 5nM
ZNF571	1.51745	siABH4#1 5nM up vs siC 5nM
ADAP2	1.51772	siABH4#1 5nM up vs siC 5nM
BLID	1.51799	siABH4#1 5nM up vs siC 5nM
PCSK6	1.51799	siABH4#1 5nM up vs siC 5nM
C4orf36	1.51814	siABH4#1 5nM up vs siC 5nM
EPB41L5	1.51824	siABH4#1 5nM up vs siC 5nM
SZT2	1.5185	siABH4#1 5nM up vs siC 5nM
SERHL2	1.51866	siABH4#1 5nM up vs siC 5nM
C1R	1.51881	siABH4#1 5nM up vs siC 5nM
LOC101926977	1.51897	siABH4#1 5nM up vs siC 5nM
C20orf96	1.5192	siABH4#1 5nM up vs siC 5nM
CPA4	1.51966	siABH4#1 5nM up vs siC 5nM
KCNQ1OT1	1.51997	siABH4#1 5nM up vs siC 5nM
FIBCD1	1.52055	siABH4#1 5nM up vs siC 5nM
LOC105374392	1.52072	siABH4#1 5nM up vs siC 5nM
MIR3913-2	1.52101	siABH4#1 5nM up vs siC 5nM
FN1	1.52126	siABH4#1 5nM up vs siC 5nM
SKIL	1.52143	siABH4#1 5nM up vs siC 5nM
MIR1976	1.52153	siABH4#1 5nM up vs siC 5nM
FUOM	1.52186	siABH4#1 5nM up vs siC 5nM
SNRPN	1.52191	siABH4#1 5nM up vs siC 5nM
SDHD	1.52211	siABH4#1 5nM up vs siC 5nM
MGRN1	1.52212	siABH4#1 5nM up vs siC 5nM
LOC102724571	1.5222	siABH4#1 5nM up vs siC 5nM
MIR1206	1.5222	siABH4#1 5nM up vs siC 5nM

GALNT5	1.52238	siABH4#1 5nM up vs siC 5nM
CALD1	1.52284	siABH4#1 5nM up vs siC 5nM
TMEM245	1.52294	siABH4#1 5nM up vs siC 5nM
HIVEP2	1.52338	siABH4#1 5nM up vs siC 5nM
SIRPA	1.52374	siABH4#1 5nM up vs siC 5nM
BCAR3	1.52438	siABH4#1 5nM up vs siC 5nM
SHPK	1.52444	siABH4#1 5nM up vs siC 5nM
IGKV1-37	1.52493	siABH4#1 5nM up vs siC 5nM
EGR1	1.52497	siABH4#1 5nM up vs siC 5nM
FAM221A	1.52532	siABH4#1 5nM up vs siC 5nM
RAB11B-AS1	1.52537	siABH4#1 5nM up vs siC 5nM
F11R	1.52593	siABH4#1 5nM up vs siC 5nM
BDH2	1.52626	siABH4#1 5nM up vs siC 5nM
NLGN2	1.52652	siABH4#1 5nM up vs siC 5nM
MIR219A1	1.52655	siABH4#1 5nM up vs siC 5nM
SCX	1.52677	siABH4#1 5nM up vs siC 5nM
SNORD114-7	1.52697	siABH4#1 5nM up vs siC 5nM
LOC729732	1.52705	siABH4#1 5nM up vs siC 5nM
ZNF30	1.52736	siABH4#1 5nM up vs siC 5nM
CLN8	1.5278	siABH4#1 5nM up vs siC 5nM
SNRPN	1.52788	siABH4#1 5nM up vs siC 5nM
PIAS3	1.52811	siABH4#1 5nM up vs siC 5nM
LOC105378975	1.52811	siABH4#1 5nM up vs siC 5nM
ACOX1	1.52814	siABH4#1 5nM up vs siC 5nM
FLJ20712	1.52827	siABH4#1 5nM up vs siC 5nM
PLCG1	1.52869	siABH4#1 5nM up vs siC 5nM
LMCD1	1.52877	siABH4#1 5nM up vs siC 5nM
SUOX	1.52891	siABH4#1 5nM up vs siC 5nM
DRICH1	1.52894	siABH4#1 5nM up vs siC 5nM
PWAR1	1.53004	siABH4#1 5nM up vs siC 5nM
IGHV4-31	1.53013	siABH4#1 5nM up vs siC 5nM
LOC105373647	1.53013	siABH4#1 5nM up vs siC 5nM

LOC105379789	1.53013	siABH4#1 5nM up vs siC 5nM
MAP3K2	1.5303	siABH4#1 5nM up vs siC 5nM
LOC105374366	1.53034	siABH4#1 5nM up vs siC 5nM
GNPTG	1.53055	siABH4#1 5nM up vs siC 5nM
OTUD1	1.53055	siABH4#1 5nM up vs siC 5nM
SCHLAP1	1.53091	siABH4#1 5nM up vs siC 5nM
LOC100134868	1.5311	siABH4#1 5nM up vs siC 5nM
BTG4	1.53137	siABH4#1 5nM up vs siC 5nM
HSF4	1.53139	siABH4#1 5nM up vs siC 5nM
ACVR1	1.53154	siABH4#1 5nM up vs siC 5nM
TEAD2	1.53155	siABH4#1 5nM up vs siC 5nM
DDX58	1.53185	siABH4#1 5nM up vs siC 5nM
DUSP16	1.5321	siABH4#1 5nM up vs siC 5nM
ZNF251	1.53222	siABH4#1 5nM up vs siC 5nM
ANKRD29	1.53304	siABH4#1 5nM up vs siC 5nM
MIR637	1.53334	siABH4#1 5nM up vs siC 5nM
ADGRA2	1.53344	siABH4#1 5nM up vs siC 5nM
LOC105374873	1.53347	siABH4#1 5nM up vs siC 5nM
LOC105376243	1.53366	siABH4#1 5nM up vs siC 5nM
SHC1	1.53387	siABH4#1 5nM up vs siC 5nM
IGKV1OR2-108	1.5339	siABH4#1 5nM up vs siC 5nM
FAM133CP	1.53411	siABH4#1 5nM up vs siC 5nM
SCN3B	1.53441	siABH4#1 5nM up vs siC 5nM
SDHC	1.53493	siABH4#1 5nM up vs siC 5nM
UBE2L6	1.53514	siABH4#1 5nM up vs siC 5nM
PLCD3	1.53518	siABH4#1 5nM up vs siC 5nM
AAMDC	1.53522	siABH4#1 5nM up vs siC 5nM
MIR4452	1.53544	siABH4#1 5nM up vs siC 5nM
TRAJ34	1.5355	siABH4#1 5nM up vs siC 5nM
DNM1	1.53613	siABH4#1 5nM up vs siC 5nM
LOC105369543	1.53625	siABH4#1 5nM up vs siC 5nM
HOMEZ	1.53634	siABH4#1 5nM up vs siC 5nM

AP2A2	1.53634	siABH4#1 5nM up vs siC 5nM
SNORA21	1.53678	siABH4#1 5nM up vs siC 5nM
GS1-124K5.11	1.53691	siABH4#1 5nM up vs siC 5nM
SERTM1	1.5372	siABH4#1 5nM up vs siC 5nM
MIR656	1.53743	siABH4#1 5nM up vs siC 5nM
ILK	1.53755	siABH4#1 5nM up vs siC 5nM
STRAP	1.5378	siABH4#1 5nM up vs siC 5nM
ZNF337-AS1	1.5381	siABH4#1 5nM up vs siC 5nM
OPTN	1.53839	siABH4#1 5nM up vs siC 5nM
METTL15	1.53886	siABH4#1 5nM up vs siC 5nM
LOC105370772	1.53936	siABH4#1 5nM up vs siC 5nM
LOC105372589	1.54025	siABH4#1 5nM up vs siC 5nM
CPNE2	1.54028	siABH4#1 5nM up vs siC 5nM
TMX3	1.54038	siABH4#1 5nM up vs siC 5nM
LOC101928401	1.54043	siABH4#1 5nM up vs siC 5nM
GOPC	1.54069	siABH4#1 5nM up vs siC 5nM
PLD3	1.54125	siABH4#1 5nM up vs siC 5nM
RSPH3	1.5417	siABH4#1 5nM up vs siC 5nM
LPXN	1.5418	siABH4#1 5nM up vs siC 5nM
ZZZ3	1.54227	siABH4#1 5nM up vs siC 5nM
CCDC92	1.54267	siABH4#1 5nM up vs siC 5nM
DNAJB5	1.54381	siABH4#1 5nM up vs siC 5nM
SNRPN	1.54435	siABH4#1 5nM up vs siC 5nM
TES	1.5444	siABH4#1 5nM up vs siC 5nM
ZFP90	1.54441	siABH4#1 5nM up vs siC 5nM
MOXD1	1.54454	siABH4#1 5nM up vs siC 5nM
MIR3911	1.54467	siABH4#1 5nM up vs siC 5nM
LIMS1	1.54518	siABH4#1 5nM up vs siC 5nM
SNORA70B	1.5452	siABH4#1 5nM up vs siC 5nM
CYB5R1	1.54531	siABH4#1 5nM up vs siC 5nM
ASPHD1	1.54561	siABH4#1 5nM up vs siC 5nM
SNAR-H	1.5457	siABH4#1 5nM up vs siC 5nM

FAM198B	1.54603	siABH4#1 5nM up vs siC 5nM
CD82	1.54627	siABH4#1 5nM up vs siC 5nM
FLJ36777	1.54663	siABH4#1 5nM up vs siC 5nM
LOC105370676	1.54689	siABH4#1 5nM up vs siC 5nM
PRRG1	1.54724	siABH4#1 5nM up vs siC 5nM
PPP1R18	1.54736	siABH4#1 5nM up vs siC 5nM
TXK	1.54749	siABH4#1 5nM up vs siC 5nM
STPG2	1.54784	siABH4#1 5nM up vs siC 5nM
KRT7	1.54876	siABH4#1 5nM up vs siC 5nM
SERINC5	1.54887	siABH4#1 5nM up vs siC 5nM
LOC105371864	1.54918	siABH4#1 5nM up vs siC 5nM
LINC00893	1.54923	siABH4#1 5nM up vs siC 5nM
ZFPL1	1.54936	siABH4#1 5nM up vs siC 5nM
ELOVL4	1.54937	siABH4#1 5nM up vs siC 5nM
FAM160A1	1.54939	siABH4#1 5nM up vs siC 5nM
OR52E6	1.54977	siABH4#1 5nM up vs siC 5nM
ZNF319	1.54987	siABH4#1 5nM up vs siC 5nM
INPP5D	1.54997	siABH4#1 5nM up vs siC 5nM
GGT1	1.55015	siABH4#1 5nM up vs siC 5nM
LENG8	1.55016	siABH4#1 5nM up vs siC 5nM
MICAL1	1.55049	siABH4#1 5nM up vs siC 5nM
LINC00475	1.55069	siABH4#1 5nM up vs siC 5nM
APPL2	1.55105	siABH4#1 5nM up vs siC 5nM
LOC105374161	1.5514	siABH4#1 5nM up vs siC 5nM
FOXN3	1.55144	siABH4#1 5nM up vs siC 5nM
COL18A1	1.55211	siABH4#1 5nM up vs siC 5nM
MIR3925	1.55214	siABH4#1 5nM up vs siC 5nM
KIRREL3-AS2	1.55239	siABH4#1 5nM up vs siC 5nM
TEP1	1.55267	siABH4#1 5nM up vs siC 5nM
TKFC	1.55273	siABH4#1 5nM up vs siC 5nM
SMAD7	1.55336	siABH4#1 5nM up vs siC 5nM
MATN2	1.55377	siABH4#1 5nM up vs siC 5nM

LGALSL	1.55396	siABH4#1 5nM up vs siC 5nM
CHPF	1.55415	siABH4#1 5nM up vs siC 5nM
SNORD114-20	1.55466	siABH4#1 5nM up vs siC 5nM
CLEC7A	1.55479	siABH4#1 5nM up vs siC 5nM
LOC105379177	1.5548	siABH4#1 5nM up vs siC 5nM
LOC105374954	1.55487	siABH4#1 5nM up vs siC 5nM
C8orf4	1.55498	siABH4#1 5nM up vs siC 5nM
LOC100128979	1.5562	siABH4#1 5nM up vs siC 5nM
LOC105373954	1.55704	siABH4#1 5nM up vs siC 5nM
MIR199B	1.55727	siABH4#1 5nM up vs siC 5nM
LOC105377329	1.5576	siABH4#1 5nM up vs siC 5nM
NACC2	1.55762	siABH4#1 5nM up vs siC 5nM
NUDT14	1.558	siABH4#1 5nM up vs siC 5nM
PRKCZ	1.55803	siABH4#1 5nM up vs siC 5nM
DBN1	1.55814	siABH4#1 5nM up vs siC 5nM
IGFBP7	1.55849	siABH4#1 5nM up vs siC 5nM
DUSP8	1.55851	siABH4#1 5nM up vs siC 5nM
RUNDC3A-AS1	1.55868	siABH4#1 5nM up vs siC 5nM
HHAT	1.55895	siABH4#1 5nM up vs siC 5nM
NUDT9P1	1.55921	siABH4#1 5nM up vs siC 5nM
ATP10D	1.55954	siABH4#1 5nM up vs siC 5nM
HSPA12A	1.55961	siABH4#1 5nM up vs siC 5nM
NBR1	1.55963	siABH4#1 5nM up vs siC 5nM
FOXP1-IT1	1.55997	siABH4#1 5nM up vs siC 5nM
FOXD4L3	1.56033	siABH4#1 5nM up vs siC 5nM
TLDC1	1.56138	siABH4#1 5nM up vs siC 5nM
SNORA65	1.56226	siABH4#1 5nM up vs siC 5nM
FLJ31356	1.56238	siABH4#1 5nM up vs siC 5nM
XYLT1	1.56245	siABH4#1 5nM up vs siC 5nM
SNRPN	1.56293	siABH4#1 5nM up vs siC 5nM
MIR3137	1.56306	siABH4#1 5nM up vs siC 5nM
MIR329-1	1.56311	siABH4#1 5nM up vs siC 5nM

PARVA	1.56333	siABH4#1 5nM up vs siC 5nM
LOC101928387	1.56366	siABH4#1 5nM up vs siC 5nM
CDH2	1.56378	siABH4#1 5nM up vs siC 5nM
KLHL5	1.56381	siABH4#1 5nM up vs siC 5nM
LOC105373621	1.56386	siABH4#1 5nM up vs siC 5nM
IQCF3	1.56411	siABH4#1 5nM up vs siC 5nM
TRAJ44	1.56412	siABH4#1 5nM up vs siC 5nM
ACKR3	1.56413	siABH4#1 5nM up vs siC 5nM
CADM4	1.56422	siABH4#1 5nM up vs siC 5nM
NEK7	1.56439	siABH4#1 5nM up vs siC 5nM
SNORA9	1.56504	siABH4#1 5nM up vs siC 5nM
IGHV1-18	1.56566	siABH4#1 5nM up vs siC 5nM
AIG1	1.56586	siABH4#1 5nM up vs siC 5nM
OBFC1	1.56627	siABH4#1 5nM up vs siC 5nM
TCP11L2	1.56658	siABH4#1 5nM up vs siC 5nM
ZSCAN30	1.56661	siABH4#1 5nM up vs siC 5nM
HOXD8	1.56676	siABH4#1 5nM up vs siC 5nM
JAKMIP2-AS1	1.56681	siABH4#1 5nM up vs siC 5nM
PSMF1	1.56687	siABH4#1 5nM up vs siC 5nM
KIAA1147	1.56715	siABH4#1 5nM up vs siC 5nM
PML	1.56747	siABH4#1 5nM up vs siC 5nM
ERV3-1	1.56768	siABH4#1 5nM up vs siC 5nM
GADD45B	1.56785	siABH4#1 5nM up vs siC 5nM
SNRPN	1.56822	siABH4#1 5nM up vs siC 5nM
PNPLA6	1.56828	siABH4#1 5nM up vs siC 5nM
LINC00623	1.56834	siABH4#1 5nM up vs siC 5nM
LPIN3	1.56844	siABH4#1 5nM up vs siC 5nM
PFKFB4	1.56844	siABH4#1 5nM up vs siC 5nM
TSSK1B	1.56881	siABH4#1 5nM up vs siC 5nM
LYRM4-AS1	1.56886	siABH4#1 5nM up vs siC 5nM
PVT1	1.56903	siABH4#1 5nM up vs siC 5nM
TAS2R40	1.56904	siABH4#1 5nM up vs siC 5nM

MXD4	1.56905	siABH4#1 5nM up vs siC 5nM
MIR548AK	1.5691	siABH4#1 5nM up vs siC 5nM
EEA1	1.56983	siABH4#1 5nM up vs siC 5nM
KLHL36	1.57004	siABH4#1 5nM up vs siC 5nM
LOC105372929	1.57004	siABH4#1 5nM up vs siC 5nM
SGCD	1.57021	siABH4#1 5nM up vs siC 5nM
CALCOCO1	1.57054	siABH4#1 5nM up vs siC 5nM
LOC105373105	1.57059	siABH4#1 5nM up vs siC 5nM
CPQ	1.57076	siABH4#1 5nM up vs siC 5nM
FBXW4	1.57138	siABH4#1 5nM up vs siC 5nM
LOC105376859	1.5717	siABH4#1 5nM up vs siC 5nM
RAB5B	1.57215	siABH4#1 5nM up vs siC 5nM
KPNA7	1.57283	siABH4#1 5nM up vs siC 5nM
GPR108	1.57331	siABH4#1 5nM up vs siC 5nM
GRHL3	1.57403	siABH4#1 5nM up vs siC 5nM
LOC101928696	1.57413	siABH4#1 5nM up vs siC 5nM
PRELID3B	1.57418	siABH4#1 5nM up vs siC 5nM
TMEM92-AS1	1.57457	siABH4#1 5nM up vs siC 5nM
CRADD	1.57458	siABH4#1 5nM up vs siC 5nM
PCDHB5	1.57463	siABH4#1 5nM up vs siC 5nM
SDC3	1.57469	siABH4#1 5nM up vs siC 5nM
LOC101929709	1.57539	siABH4#1 5nM up vs siC 5nM
MKL1	1.57696	siABH4#1 5nM up vs siC 5nM
ARHGEF17	1.57723	siABH4#1 5nM up vs siC 5nM
TEX9	1.57729	siABH4#1 5nM up vs siC 5nM
MIR4253	1.57738	siABH4#1 5nM up vs siC 5nM
TNRC6C	1.57813	siABH4#1 5nM up vs siC 5nM
CEACAM19	1.57813	siABH4#1 5nM up vs siC 5nM
MS4A4A	1.57839	siABH4#1 5nM up vs siC 5nM
RFX3-AS1	1.57839	siABH4#1 5nM up vs siC 5nM
KRTAP2-3	1.57946	siABH4#1 5nM up vs siC 5nM
TCIRG1	1.57964	siABH4#1 5nM up vs siC 5nM

RABL2B	1.57973	siABH4#1 5nM up vs siC 5nM
RASA4B	1.57992	siABH4#1 5nM up vs siC 5nM
CAPG	1.58046	siABH4#1 5nM up vs siC 5nM
HLA-H	1.58216	siABH4#1 5nM up vs siC 5nM
ABCA10	1.58253	siABH4#1 5nM up vs siC 5nM
MIR2355	1.58287	siABH4#1 5nM up vs siC 5nM
GGT3P	1.58316	siABH4#1 5nM up vs siC 5nM
LOC105370287	1.58386	siABH4#1 5nM up vs siC 5nM
LINC01545	1.58399	siABH4#1 5nM up vs siC 5nM
MIR4445	1.58497	siABH4#1 5nM up vs siC 5nM
PVT1	1.58501	siABH4#1 5nM up vs siC 5nM
SNORD113-4	1.58533	siABH4#1 5nM up vs siC 5nM
PDHX	1.58534	siABH4#1 5nM up vs siC 5nM
SPATA31D4	1.58579	siABH4#1 5nM up vs siC 5nM
GCSH	1.58582	siABH4#1 5nM up vs siC 5nM
PPP1R2	1.58586	siABH4#1 5nM up vs siC 5nM
PRKXP1	1.58603	siABH4#1 5nM up vs siC 5nM
MIR519A2	1.58603	siABH4#1 5nM up vs siC 5nM
TRIM78P	1.58727	siABH4#1 5nM up vs siC 5nM
POU4F1	1.5873	siABH4#1 5nM up vs siC 5nM
TMEM91	1.58823	siABH4#1 5nM up vs siC 5nM
SLC12A7	1.58906	siABH4#1 5nM up vs siC 5nM
CNOT6	1.5892	siABH4#1 5nM up vs siC 5nM
GPRC5C	1.58927	siABH4#1 5nM up vs siC 5nM
PERP	1.58955	siABH4#1 5nM up vs siC 5nM
GRINA	1.58981	siABH4#1 5nM up vs siC 5nM
HMGCL	1.59009	siABH4#1 5nM up vs siC 5nM
NOP14-AS1	1.59046	siABH4#1 5nM up vs siC 5nM
GCC2-AS1	1.59047	siABH4#1 5nM up vs siC 5nM
LOC101930105	1.59064	siABH4#1 5nM up vs siC 5nM
MOB3C	1.59086	siABH4#1 5nM up vs siC 5nM
LOC101927542	1.5909	siABH4#1 5nM up vs siC 5nM

LOC105370424	1.5909	siABH4#1 5nM up vs siC 5nM
FOXD4	1.591	siABH4#1 5nM up vs siC 5nM
LOC105376244	1.59116	siABH4#1 5nM up vs siC 5nM
BAZ2B	1.5912	siABH4#1 5nM up vs siC 5nM
CRYGB	1.59131	siABH4#1 5nM up vs siC 5nM
C4orf33	1.59152	siABH4#1 5nM up vs siC 5nM
PIGZ	1.59153	siABH4#1 5nM up vs siC 5nM
LOC101927121	1.59166	siABH4#1 5nM up vs siC 5nM
TPTE2P6	1.59166	siABH4#1 5nM up vs siC 5nM
MSRB2	1.59202	siABH4#1 5nM up vs siC 5nM
FAM86B2	1.59213	siABH4#1 5nM up vs siC 5nM
LMF1	1.59219	siABH4#1 5nM up vs siC 5nM
FBXL2	1.59248	siABH4#1 5nM up vs siC 5nM
HID1	1.59277	siABH4#1 5nM up vs siC 5nM
PPM1D	1.59279	siABH4#1 5nM up vs siC 5nM
SNTN	1.59299	siABH4#1 5nM up vs siC 5nM
CFB	1.5932	siABH4#1 5nM up vs siC 5nM
CFAP53	1.59321	siABH4#1 5nM up vs siC 5nM
LOC102724851	1.59374	siABH4#1 5nM up vs siC 5nM
ASCL2	1.59374	siABH4#1 5nM up vs siC 5nM
IGHG3	1.59382	siABH4#1 5nM up vs siC 5nM
YES1	1.59388	siABH4#1 5nM up vs siC 5nM
PYGO1	1.59469	siABH4#1 5nM up vs siC 5nM
MIR34A	1.59473	siABH4#1 5nM up vs siC 5nM
CTBS	1.59522	siABH4#1 5nM up vs siC 5nM
MFAP3	1.59526	siABH4#1 5nM up vs siC 5nM
MIR181A1	1.59557	siABH4#1 5nM up vs siC 5nM
PLGLB2	1.59562	siABH4#1 5nM up vs siC 5nM
CERS4	1.59598	siABH4#1 5nM up vs siC 5nM
LOC105378114	1.59644	siABH4#1 5nM up vs siC 5nM
PRKXP1	1.59711	siABH4#1 5nM up vs siC 5nM
FER1L4	1.59735	siABH4#1 5nM up vs siC 5nM

XKRX	1.59771	siABH4#1 5nM up vs siC 5nM
DDX60L	1.59814	siABH4#1 5nM up vs siC 5nM
UBE2H	1.59873	siABH4#1 5nM up vs siC 5nM
SMAD2	1.59889	siABH4#1 5nM up vs siC 5nM
CREBRF	1.5992	siABH4#1 5nM up vs siC 5nM
PDCD4	1.59925	siABH4#1 5nM up vs siC 5nM
LOC105379272	1.59926	siABH4#1 5nM up vs siC 5nM
C19orf54	1.59931	siABH4#1 5nM up vs siC 5nM
IGKV3-11	1.59945	siABH4#1 5nM up vs siC 5nM
JMY	1.60042	siABH4#1 5nM up vs siC 5nM
PRR4	1.60056	siABH4#1 5nM up vs siC 5nM
LOC105376287	1.60072	siABH4#1 5nM up vs siC 5nM
LRP12	1.60139	siABH4#1 5nM up vs siC 5nM
C5	1.60173	siABH4#1 5nM up vs siC 5nM
SMIM14	1.60177	siABH4#1 5nM up vs siC 5nM
DNAH1	1.60228	siABH4#1 5nM up vs siC 5nM
PARP8	1.60247	siABH4#1 5nM up vs siC 5nM
LOC105377989	1.60262	siABH4#1 5nM up vs siC 5nM
MIF4GD	1.60328	siABH4#1 5nM up vs siC 5nM
MIR27B	1.60355	siABH4#1 5nM up vs siC 5nM
ZNF544	1.60364	siABH4#1 5nM up vs siC 5nM
ITPR2	1.60375	siABH4#1 5nM up vs siC 5nM
LOC101930100	1.60392	siABH4#1 5nM up vs siC 5nM
FBXO22	1.60418	siABH4#1 5nM up vs siC 5nM
GYG2	1.60635	siABH4#1 5nM up vs siC 5nM
ANKRD46	1.60651	siABH4#1 5nM up vs siC 5nM
CFAP126	1.60682	siABH4#1 5nM up vs siC 5nM
ARAP2	1.60684	siABH4#1 5nM up vs siC 5nM
FAM66B	1.60692	siABH4#1 5nM up vs siC 5nM
ALOX12P2	1.6071	siABH4#1 5nM up vs siC 5nM
ADRB2	1.60727	siABH4#1 5nM up vs siC 5nM
MITF	1.6073	siABH4#1 5nM up vs siC 5nM

ZYX	1.60771	siABH4#1 5nM up vs siC 5nM
LOC105369575	1.60792	siABH4#1 5nM up vs siC 5nM
PKP2	1.608	siABH4#1 5nM up vs siC 5nM
NDUFAF6	1.608	siABH4#1 5nM up vs siC 5nM
TGOLN2	1.60904	siABH4#1 5nM up vs siC 5nM
FOXN3-AS1	1.60909	siABH4#1 5nM up vs siC 5nM
NUAK1	1.60937	siABH4#1 5nM up vs siC 5nM
STX4	1.61008	siABH4#1 5nM up vs siC 5nM
SDC4	1.61049	siABH4#1 5nM up vs siC 5nM
MIR520H	1.61128	siABH4#1 5nM up vs siC 5nM
SAP30L-AS1	1.61176	siABH4#1 5nM up vs siC 5nM
YOD1	1.61225	siABH4#1 5nM up vs siC 5nM
STAG3L1	1.61228	siABH4#1 5nM up vs siC 5nM
MFI2	1.61247	siABH4#1 5nM up vs siC 5nM
ZNF280D	1.61289	siABH4#1 5nM up vs siC 5nM
LINC01376	1.61302	siABH4#1 5nM up vs siC 5nM
MIRLET7E	1.61337	siABH4#1 5nM up vs siC 5nM
SLC26A2	1.61347	siABH4#1 5nM up vs siC 5nM
VASN	1.61373	siABH4#1 5nM up vs siC 5nM
GAREM1	1.61531	siABH4#1 5nM up vs siC 5nM
LAMC2	1.61626	siABH4#1 5nM up vs siC 5nM
TMEM181	1.61645	siABH4#1 5nM up vs siC 5nM
CCDC162P	1.61663	siABH4#1 5nM up vs siC 5nM
MIR376B	1.61673	siABH4#1 5nM up vs siC 5nM
LOC105377949	1.61676	siABH4#1 5nM up vs siC 5nM
ZKSCAN1	1.61726	siABH4#1 5nM up vs siC 5nM
FBLIM1	1.61778	siABH4#1 5nM up vs siC 5nM
MOCS2	1.61797	siABH4#1 5nM up vs siC 5nM
CLDN16	1.61799	siABH4#1 5nM up vs siC 5nM
CDYL2	1.61851	siABH4#1 5nM up vs siC 5nM
SLC26A7	1.61876	siABH4#1 5nM up vs siC 5nM
AGRN	1.61891	siABH4#1 5nM up vs siC 5nM

ATHL1	1.61977	siABH4#1 5nM up vs siC 5nM
MIR3189	1.62001	siABH4#1 5nM up vs siC 5nM
AKAP2	1.62005	siABH4#1 5nM up vs siC 5nM
RASA4B	1.62073	siABH4#1 5nM up vs siC 5nM
C10orf55	1.62079	siABH4#1 5nM up vs siC 5nM
PLD5	1.62079	siABH4#1 5nM up vs siC 5nM
MIR181B1	1.6213	siABH4#1 5nM up vs siC 5nM
IGKV2-24	1.62192	siABH4#1 5nM up vs siC 5nM
GANC	1.62193	siABH4#1 5nM up vs siC 5nM
GRIN2B	1.62261	siABH4#1 5nM up vs siC 5nM
NRBF2	1.6227	siABH4#1 5nM up vs siC 5nM
ARL1	1.6231	siABH4#1 5nM up vs siC 5nM
VTRNA1-1	1.62354	siABH4#1 5nM up vs siC 5nM
FAM229B	1.62355	siABH4#1 5nM up vs siC 5nM
MIR4500	1.62362	siABH4#1 5nM up vs siC 5nM
MIR3674	1.62387	siABH4#1 5nM up vs siC 5nM
MIR370	1.62501	siABH4#1 5nM up vs siC 5nM
GUCY1B2	1.62504	siABH4#1 5nM up vs siC 5nM
TRAV12-3	1.62514	siABH4#1 5nM up vs siC 5nM
LINC00963	1.62563	siABH4#1 5nM up vs siC 5nM
BMPR2	1.62572	siABH4#1 5nM up vs siC 5nM
ADAMTSL5	1.62576	siABH4#1 5nM up vs siC 5nM
AP1G2	1.62619	siABH4#1 5nM up vs siC 5nM
ABHD2	1.6272	siABH4#1 5nM up vs siC 5nM
MIR487A	1.6273	siABH4#1 5nM up vs siC 5nM
CTD-2194D22.4	1.62744	siABH4#1 5nM up vs siC 5nM
PRKACA	1.62775	siABH4#1 5nM up vs siC 5nM
PYROXD2	1.6282	siABH4#1 5nM up vs siC 5nM
LOC105378881	1.62905	siABH4#1 5nM up vs siC 5nM
IRF2BPL	1.62914	siABH4#1 5nM up vs siC 5nM
LOC441666	1.62939	siABH4#1 5nM up vs siC 5nM
TPK1	1.62984	siABH4#1 5nM up vs siC 5nM

LOC105370741	1.63002	siABH4#1 5nM up vs siC 5nM
TNFRSF10C	1.63027	siABH4#1 5nM up vs siC 5nM
SP140L	1.6312	siABH4#1 5nM up vs siC 5nM
LOC105372626	1.63174	siABH4#1 5nM up vs siC 5nM
PNRC1	1.63188	siABH4#1 5nM up vs siC 5nM
TMEM117	1.63198	siABH4#1 5nM up vs siC 5nM
SPRED1	1.63215	siABH4#1 5nM up vs siC 5nM
MAN2C1	1.63285	siABH4#1 5nM up vs siC 5nM
COL5A1	1.63289	siABH4#1 5nM up vs siC 5nM
FSCN2	1.633	siABH4#1 5nM up vs siC 5nM
RABL2A	1.6344	siABH4#1 5nM up vs siC 5nM
LYRM4	1.6348	siABH4#1 5nM up vs siC 5nM
PDLIM1	1.63491	siABH4#1 5nM up vs siC 5nM
ANKRD20A12P	1.63522	siABH4#1 5nM up vs siC 5nM
AKR1C8P	1.63522	siABH4#1 5nM up vs siC 5nM
HLA-B	1.63522	siABH4#1 5nM up vs siC 5nM
SMC5-AS1	1.63534	siABH4#1 5nM up vs siC 5nM
SNORD105	1.63601	siABH4#1 5nM up vs siC 5nM
AMPD3	1.63625	siABH4#1 5nM up vs siC 5nM
LOC100270804	1.6368	siABH4#1 5nM up vs siC 5nM
MIR4718	1.6368	siABH4#1 5nM up vs siC 5nM
CMTM3	1.63685	siABH4#1 5nM up vs siC 5nM
PHLDB3	1.6372	siABH4#1 5nM up vs siC 5nM
COBL	1.63743	siABH4#1 5nM up vs siC 5nM
KDM3A	1.63759	siABH4#1 5nM up vs siC 5nM
PARK2	1.638	siABH4#1 5nM up vs siC 5nM
GLRB	1.63831	siABH4#1 5nM up vs siC 5nM
SLCO1B7	1.63896	siABH4#1 5nM up vs siC 5nM
NKX2-5	1.63914	siABH4#1 5nM up vs siC 5nM
OGFRL1	1.63935	siABH4#1 5nM up vs siC 5nM
SLC44A3	1.63952	siABH4#1 5nM up vs siC 5nM
ACY3	1.64008	siABH4#1 5nM up vs siC 5nM

43892	1.64013	siABH4#1 5nM up vs siC 5nM
PGA5	1.64103	siABH4#1 5nM up vs siC 5nM
TROVE2	1.64113	siABH4#1 5nM up vs siC 5nM
CHAF1B	1.64147	siABH4#1 5nM up vs siC 5nM
CCDC126	1.64147	siABH4#1 5nM up vs siC 5nM
DBET	1.64148	siABH4#1 5nM up vs siC 5nM
CD38	1.64156	siABH4#1 5nM up vs siC 5nM
PPARA	1.64182	siABH4#1 5nM up vs siC 5nM
ACOT8	1.64203	siABH4#1 5nM up vs siC 5nM
ZP3	1.64214	siABH4#1 5nM up vs siC 5nM
GKAP1	1.64234	siABH4#1 5nM up vs siC 5nM
IMPACT	1.64266	siABH4#1 5nM up vs siC 5nM
HSD17B1	1.64283	siABH4#1 5nM up vs siC 5nM
LOC641746	1.64294	siABH4#1 5nM up vs siC 5nM
TMEM88	1.64332	siABH4#1 5nM up vs siC 5nM
ZNF252P	1.64337	siABH4#1 5nM up vs siC 5nM
NPIPB6	1.64423	siABH4#1 5nM up vs siC 5nM
MIR23A	1.64445	siABH4#1 5nM up vs siC 5nM
IGIP	1.64445	siABH4#1 5nM up vs siC 5nM
TP53I11	1.64449	siABH4#1 5nM up vs siC 5nM
ACP6	1.64522	siABH4#1 5nM up vs siC 5nM
PPP3CA	1.64527	siABH4#1 5nM up vs siC 5nM
LOC101928478	1.64637	siABH4#1 5nM up vs siC 5nM
FAR2P1	1.64696	siABH4#1 5nM up vs siC 5nM
GSN	1.64714	siABH4#1 5nM up vs siC 5nM
BHLHE40	1.64715	siABH4#1 5nM up vs siC 5nM
WBSCR27	1.64759	siABH4#1 5nM up vs siC 5nM
COL12A1	1.64812	siABH4#1 5nM up vs siC 5nM
CUBN	1.64829	siABH4#1 5nM up vs siC 5nM
GLTP	1.64844	siABH4#1 5nM up vs siC 5nM
OR7C1	1.6485	siABH4#1 5nM up vs siC 5nM
RAB42	1.64877	siABH4#1 5nM up vs siC 5nM

RIN2	1.64892	siABH4#1 5nM up vs siC 5nM
PCMTD1	1.649	siABH4#1 5nM up vs siC 5nM
ARMC9	1.6494	siABH4#1 5nM up vs siC 5nM
LDLRAD4	1.64953	siABH4#1 5nM up vs siC 5nM
DCP1B	1.64989	siABH4#1 5nM up vs siC 5nM
SMTN	1.65065	siABH4#1 5nM up vs siC 5nM
OR4F17	1.65073	siABH4#1 5nM up vs siC 5nM
SGMS1-AS1	1.65095	siABH4#1 5nM up vs siC 5nM
SLC16A2	1.65099	siABH4#1 5nM up vs siC 5nM
MACROD2-IT1	1.65126	siABH4#1 5nM up vs siC 5nM
CA8	1.65214	siABH4#1 5nM up vs siC 5nM
UBAP1	1.65246	siABH4#1 5nM up vs siC 5nM
ZNF641	1.65257	siABH4#1 5nM up vs siC 5nM
MAN2A1	1.653	siABH4#1 5nM up vs siC 5nM
ATP2A1	1.6531	siABH4#1 5nM up vs siC 5nM
HYI	1.65418	siABH4#1 5nM up vs siC 5nM
FLJ26850	1.65607	siABH4#1 5nM up vs siC 5nM
GNB5	1.65637	siABH4#1 5nM up vs siC 5nM
CDH6	1.65753	siABH4#1 5nM up vs siC 5nM
GARNL3	1.65768	siABH4#1 5nM up vs siC 5nM
CCDC18-AS1	1.65847	siABH4#1 5nM up vs siC 5nM
GAB2	1.65888	siABH4#1 5nM up vs siC 5nM
PPP1R13L	1.65902	siABH4#1 5nM up vs siC 5nM
S100A4	1.66023	siABH4#1 5nM up vs siC 5nM
GS1-259H13.2	1.66044	siABH4#1 5nM up vs siC 5nM
PTPRG	1.66124	siABH4#1 5nM up vs siC 5nM
DENND6A	1.66194	siABH4#1 5nM up vs siC 5nM
PID1	1.66198	siABH4#1 5nM up vs siC 5nM
CTSK	1.66208	siABH4#1 5nM up vs siC 5nM
CDC42EP4	1.66229	siABH4#1 5nM up vs siC 5nM
RGAG4	1.66241	siABH4#1 5nM up vs siC 5nM
LOXL4	1.66277	siABH4#1 5nM up vs siC 5nM

LINC01191	1.66302	siABH4#1 5nM up vs siC 5nM
ITGAV	1.66312	siABH4#1 5nM up vs siC 5nM
LOC202181	1.66384	siABH4#1 5nM up vs siC 5nM
IFNGR1	1.6639	siABH4#1 5nM up vs siC 5nM
PCDHB4	1.66406	siABH4#1 5nM up vs siC 5nM
HNRNPKP3	1.6644	siABH4#1 5nM up vs siC 5nM
LOC105370173	1.6644	siABH4#1 5nM up vs siC 5nM
PTBP3	1.66538	siABH4#1 5nM up vs siC 5nM
UBQLNL	1.66568	siABH4#1 5nM up vs siC 5nM
OTUD6B-AS1	1.66572	siABH4#1 5nM up vs siC 5nM
LOC101929577	1.66581	siABH4#1 5nM up vs siC 5nM
UBALD1	1.66629	siABH4#1 5nM up vs siC 5nM
SNORD3D	1.66634	siABH4#1 5nM up vs siC 5nM
NALCN	1.66651	siABH4#1 5nM up vs siC 5nM
SLC9A6	1.66682	siABH4#1 5nM up vs siC 5nM
EGOT	1.66706	siABH4#1 5nM up vs siC 5nM
SNORA58	1.6671	siABH4#1 5nM up vs siC 5nM
SLC2A6	1.66724	siABH4#1 5nM up vs siC 5nM
RNF217	1.66739	siABH4#1 5nM up vs siC 5nM
FETUB	1.66843	siABH4#1 5nM up vs siC 5nM
SNORA48	1.6686	siABH4#1 5nM up vs siC 5nM
SOX12	1.66895	siABH4#1 5nM up vs siC 5nM
HNRNPA1P33	1.66897	siABH4#1 5nM up vs siC 5nM
MIR598	1.6695	siABH4#1 5nM up vs siC 5nM
SLC44A5	1.66962	siABH4#1 5nM up vs siC 5nM
B3GAT3	1.67054	siABH4#1 5nM up vs siC 5nM
MIR4473	1.67078	siABH4#1 5nM up vs siC 5nM
LOC100128281	1.67174	siABH4#1 5nM up vs siC 5nM
PLA2G10	1.67232	siABH4#1 5nM up vs siC 5nM
HCG4B	1.67232	siABH4#1 5nM up vs siC 5nM
ADRA2C	1.67244	siABH4#1 5nM up vs siC 5nM
SERTAD2	1.67265	siABH4#1 5nM up vs siC 5nM

MROH1	1.67327	siABH4#1 5nM up vs siC 5nM
PAPPA	1.67377	siABH4#1 5nM up vs siC 5nM
ZNF767P	1.67394	siABH4#1 5nM up vs siC 5nM
LINC01198	1.67415	siABH4#1 5nM up vs siC 5nM
PCMTD1	1.67415	siABH4#1 5nM up vs siC 5nM
DNASE1L1	1.67441	siABH4#1 5nM up vs siC 5nM
RNF217-AS1	1.67442	siABH4#1 5nM up vs siC 5nM
AASS	1.67444	siABH4#1 5nM up vs siC 5nM
LINC00322	1.67506	siABH4#1 5nM up vs siC 5nM
WIPF1	1.67508	siABH4#1 5nM up vs siC 5nM
LOC105374160	1.67547	siABH4#1 5nM up vs siC 5nM
LOC100419773	1.67607	siABH4#1 5nM up vs siC 5nM
RIN1	1.67623	siABH4#1 5nM up vs siC 5nM
EPHX1	1.679	siABH4#1 5nM up vs siC 5nM
WBP1	1.67974	siABH4#1 5nM up vs siC 5nM
LOC645553	1.6804	siABH4#1 5nM up vs siC 5nM
DUX4L25	1.68073	siABH4#1 5nM up vs siC 5nM
DUX4L25	1.68073	siABH4#1 5nM up vs siC 5nM
TRAPPC6A	1.68186	siABH4#1 5nM up vs siC 5nM
FAM86FP	1.68261	siABH4#1 5nM up vs siC 5nM
MAFIP	1.68345	siABH4#1 5nM up vs siC 5nM
SNORD113-2	1.68346	siABH4#1 5nM up vs siC 5nM
XIAP	1.6839	siABH4#1 5nM up vs siC 5nM
TLE4	1.68467	siABH4#1 5nM up vs siC 5nM
TGFBI	1.68527	siABH4#1 5nM up vs siC 5nM
BAX	1.68621	siABH4#1 5nM up vs siC 5nM
MIR137	1.68632	siABH4#1 5nM up vs siC 5nM
LOC283435	1.68666	siABH4#1 5nM up vs siC 5nM
TRAJ35	1.68699	siABH4#1 5nM up vs siC 5nM
MIR378A	1.68699	siABH4#1 5nM up vs siC 5nM
LAMB3	1.6871	siABH4#1 5nM up vs siC 5nM
ZNF205	1.68731	siABH4#1 5nM up vs siC 5nM

LOC105377774	1.68761	siABH4#1 5nM up vs siC 5nM
LOC102723354	1.68768	siABH4#1 5nM up vs siC 5nM
MAN2B1	1.68801	siABH4#1 5nM up vs siC 5nM
MXI1	1.68804	siABH4#1 5nM up vs siC 5nM
SMIM6	1.68829	siABH4#1 5nM up vs siC 5nM
FBN1	1.68958	siABH4#1 5nM up vs siC 5nM
MIR320B1	1.68968	siABH4#1 5nM up vs siC 5nM
MIR620	1.68968	siABH4#1 5nM up vs siC 5nM
OR10AB1P	1.69027	siABH4#1 5nM up vs siC 5nM
GLIPR2	1.69175	siABH4#1 5nM up vs siC 5nM
LOC102723533	1.69213	siABH4#1 5nM up vs siC 5nM
MIR103A2	1.69223	siABH4#1 5nM up vs siC 5nM
LOC105376150	1.69254	siABH4#1 5nM up vs siC 5nM
PNRC2	1.6929	siABH4#1 5nM up vs siC 5nM
SNORD116-15	1.69306	siABH4#1 5nM up vs siC 5nM
PIKFYVE	1.69334	siABH4#1 5nM up vs siC 5nM
SNORD114-27	1.69408	siABH4#1 5nM up vs siC 5nM
TSPYL1	1.69443	siABH4#1 5nM up vs siC 5nM
HEXIM2	1.69459	siABH4#1 5nM up vs siC 5nM
LOC105372516	1.69504	siABH4#1 5nM up vs siC 5nM
SNORD116-14	1.69506	siABH4#1 5nM up vs siC 5nM
COL7A1	1.69601	siABH4#1 5nM up vs siC 5nM
ABLIM1	1.69665	siABH4#1 5nM up vs siC 5nM
ASCC3	1.69687	siABH4#1 5nM up vs siC 5nM
MIR544A	1.69709	siABH4#1 5nM up vs siC 5nM
SNORD114-16	1.69746	siABH4#1 5nM up vs siC 5nM
NIPAL4	1.69751	siABH4#1 5nM up vs siC 5nM
APOBEC3D	1.69903	siABH4#1 5nM up vs siC 5nM
LOC105372013	1.6991	siABH4#1 5nM up vs siC 5nM
STAT2	1.69925	siABH4#1 5nM up vs siC 5nM
CYP2C19	1.69926	siABH4#1 5nM up vs siC 5nM
LOC105369812	1.69926	siABH4#1 5nM up vs siC 5nM

DIRC2	1.69928	siABH4#1 5nM up vs siC 5nM
PKI55	1.70024	siABH4#1 5nM up vs siC 5nM
RBMS2	1.70101	siABH4#1 5nM up vs siC 5nM
CASK	1.70106	siABH4#1 5nM up vs siC 5nM
SNORD114-18	1.70206	siABH4#1 5nM up vs siC 5nM
DYSF	1.7021	siABH4#1 5nM up vs siC 5nM
LOC105374417	1.70271	siABH4#1 5nM up vs siC 5nM
MIR32	1.70277	siABH4#1 5nM up vs siC 5nM
CCAT1	1.70297	siABH4#1 5nM up vs siC 5nM
MIR4290	1.70301	siABH4#1 5nM up vs siC 5nM
PDE10A	1.70322	siABH4#1 5nM up vs siC 5nM
TRIM66	1.70325	siABH4#1 5nM up vs siC 5nM
ADAM19	1.70355	siABH4#1 5nM up vs siC 5nM
PAX6	1.70416	siABH4#1 5nM up vs siC 5nM
KIRREL3	1.70473	siABH4#1 5nM up vs siC 5nM
LOC105374715	1.70546	siABH4#1 5nM up vs siC 5nM
MSRB3	1.7058	siABH4#1 5nM up vs siC 5nM
SYT17	1.70719	siABH4#1 5nM up vs siC 5nM
MIR517A	1.70788	siABH4#1 5nM up vs siC 5nM
PVRL2	1.70826	siABH4#1 5nM up vs siC 5nM
LOC105377023	1.7084	siABH4#1 5nM up vs siC 5nM
TMEM253	1.70852	siABH4#1 5nM up vs siC 5nM
ZNF658B	1.70875	siABH4#1 5nM up vs siC 5nM
TGFB2	1.70887	siABH4#1 5nM up vs siC 5nM
POTEI	1.709	siABH4#1 5nM up vs siC 5nM
ANKRD20A5P	1.71009	siABH4#1 5nM up vs siC 5nM
MIR4499	1.71081	siABH4#1 5nM up vs siC 5nM
MIR485	1.71103	siABH4#1 5nM up vs siC 5nM
GPR87	1.71186	siABH4#1 5nM up vs siC 5nM
ADAM20P1	1.71262	siABH4#1 5nM up vs siC 5nM
ROBO1	1.71267	siABH4#1 5nM up vs siC 5nM
UBE2CP5	1.71277	siABH4#1 5nM up vs siC 5nM

PWARSN	1.71315	siABH4#1 5nM up vs siC 5nM
LINC00998	1.71403	siABH4#1 5nM up vs siC 5nM
LINGO2	1.71403	siABH4#1 5nM up vs siC 5nM
TEX41	1.71415	siABH4#1 5nM up vs siC 5nM
PCED1A	1.7142	siABH4#1 5nM up vs siC 5nM
SNRPN	1.71618	siABH4#1 5nM up vs siC 5nM
LOC105370728	1.71657	siABH4#1 5nM up vs siC 5nM
ZNF264	1.71693	siABH4#1 5nM up vs siC 5nM
SLC25A23	1.71697	siABH4#1 5nM up vs siC 5nM
FAM189A1	1.718	siABH4#1 5nM up vs siC 5nM
CCL5	1.71814	siABH4#1 5nM up vs siC 5nM
TVP23C	1.71879	siABH4#1 5nM up vs siC 5nM
SNORA71A	1.71962	siABH4#1 5nM up vs siC 5nM
CATSPER2P1	1.7197	siABH4#1 5nM up vs siC 5nM
TCP11L1	1.71994	siABH4#1 5nM up vs siC 5nM
FBXL13	1.71999	siABH4#1 5nM up vs siC 5nM
SLC6A16	1.72015	siABH4#1 5nM up vs siC 5nM
CBLN3	1.72022	siABH4#1 5nM up vs siC 5nM
RIC1	1.72115	siABH4#1 5nM up vs siC 5nM
DUX4L25	1.72118	siABH4#1 5nM up vs siC 5nM
LOC100132057	1.72199	siABH4#1 5nM up vs siC 5nM
TANC2	1.72211	siABH4#1 5nM up vs siC 5nM
TMEM198B	1.72224	siABH4#1 5nM up vs siC 5nM
ORAI3	1.72244	siABH4#1 5nM up vs siC 5nM
HIST2H2BE	1.72246	siABH4#1 5nM up vs siC 5nM
SLC12A4	1.72355	siABH4#1 5nM up vs siC 5nM
SUSD6	1.72418	siABH4#1 5nM up vs siC 5nM
ANXA13	1.72481	siABH4#1 5nM up vs siC 5nM
KIFC2	1.72495	siABH4#1 5nM up vs siC 5nM
HSP90AB2P	1.72503	siABH4#1 5nM up vs siC 5nM
COL4A2	1.72533	siABH4#1 5nM up vs siC 5nM
NRSN2-AS1	1.72551	siABH4#1 5nM up vs siC 5nM

DDB2	1.72564	siABH4#1 5nM up vs siC 5nM
MIR18B	1.72599	siABH4#1 5nM up vs siC 5nM
UBL3	1.7264	siABH4#1 5nM up vs siC 5nM
GOLGA1	1.7267	siABH4#1 5nM up vs siC 5nM
CSMD3	1.72779	siABH4#1 5nM up vs siC 5nM
LOC105376774	1.7279	siABH4#1 5nM up vs siC 5nM
RARRES3	1.72816	siABH4#1 5nM up vs siC 5nM
KCNMA1	1.7282	siABH4#1 5nM up vs siC 5nM
BBC3	1.72853	siABH4#1 5nM up vs siC 5nM
RELB	1.72922	siABH4#1 5nM up vs siC 5nM
NPTXR	1.72926	siABH4#1 5nM up vs siC 5nM
NABP1	1.72933	siABH4#1 5nM up vs siC 5nM
OR1J4	1.72962	siABH4#1 5nM up vs siC 5nM
HOXB9	1.72999	siABH4#1 5nM up vs siC 5nM
MMP2	1.73004	siABH4#1 5nM up vs siC 5nM
SORT1	1.73035	siABH4#1 5nM up vs siC 5nM
ADAMTS10	1.73168	siABH4#1 5nM up vs siC 5nM
IL4R	1.73196	siABH4#1 5nM up vs siC 5nM
SCRN2	1.73325	siABH4#1 5nM up vs siC 5nM
PDGFC	1.73377	siABH4#1 5nM up vs siC 5nM
BPIFA4P	1.73381	siABH4#1 5nM up vs siC 5nM
GATSL3	1.73392	siABH4#1 5nM up vs siC 5nM
VTRNA1-2	1.73434	siABH4#1 5nM up vs siC 5nM
ZNF117	1.73465	siABH4#1 5nM up vs siC 5nM
CHRNA7	1.73539	siABH4#1 5nM up vs siC 5nM
LINC00294	1.73622	siABH4#1 5nM up vs siC 5nM
ZNRD1-AS1	1.73634	siABH4#1 5nM up vs siC 5nM
LOC105371702	1.73634	siABH4#1 5nM up vs siC 5nM
NEXN	1.73664	siABH4#1 5nM up vs siC 5nM
SULT1C2	1.73694	siABH4#1 5nM up vs siC 5nM
FAM117A	1.73701	siABH4#1 5nM up vs siC 5nM
CCL3L1	1.7372	siABH4#1 5nM up vs siC 5nM

NFKBIA	1.73741	siABH4#1 5nM up vs siC 5nM
MIR619	1.73812	siABH4#1 5nM up vs siC 5nM
PNPLA4	1.73841	siABH4#1 5nM up vs siC 5nM
PLSCR3	1.73867	siABH4#1 5nM up vs siC 5nM
SNRPN	1.73918	siABH4#1 5nM up vs siC 5nM
LIPH	1.73957	siABH4#1 5nM up vs siC 5nM
SGSM2	1.74025	siABH4#1 5nM up vs siC 5nM
LOC644794	1.74093	siABH4#1 5nM up vs siC 5nM
CEACAM1	1.7412	siABH4#1 5nM up vs siC 5nM
APLF	1.74277	siABH4#1 5nM up vs siC 5nM
ENO3	1.74317	siABH4#1 5nM up vs siC 5nM
FOS	1.74379	siABH4#1 5nM up vs siC 5nM
TM7SF3	1.74479	siABH4#1 5nM up vs siC 5nM
USP46	1.74479	siABH4#1 5nM up vs siC 5nM
SELM	1.74486	siABH4#1 5nM up vs siC 5nM
LOC101060445	1.74521	siABH4#1 5nM up vs siC 5nM
SSH3	1.74555	siABH4#1 5nM up vs siC 5nM
ANKRD26	1.74618	siABH4#1 5nM up vs siC 5nM
OSBPL7	1.74721	siABH4#1 5nM up vs siC 5nM
PGGT1B	1.74815	siABH4#1 5nM up vs siC 5nM
RASSF3	1.7485	siABH4#1 5nM up vs siC 5nM
FAM86DP	1.74881	siABH4#1 5nM up vs siC 5nM
LINC01284	1.74885	siABH4#1 5nM up vs siC 5nM
LOC105378367	1.74958	siABH4#1 5nM up vs siC 5nM
GOLT1A	1.75148	siABH4#1 5nM up vs siC 5nM
SGSH	1.75193	siABH4#1 5nM up vs siC 5nM
EVI5	1.75203	siABH4#1 5nM up vs siC 5nM
PGAP1	1.75321	siABH4#1 5nM up vs siC 5nM
LOC105375464	1.75342	siABH4#1 5nM up vs siC 5nM
NLK	1.75551	siABH4#1 5nM up vs siC 5nM
CADM1	1.75667	siABH4#1 5nM up vs siC 5nM
ACYP2	1.75793	siABH4#1 5nM up vs siC 5nM

GPR135	1.75815	siABH4#1 5nM up vs siC 5nM
MMP24-AS1	1.75877	siABH4#1 5nM up vs siC 5nM
NBEAL1	1.75908	siABH4#1 5nM up vs siC 5nM
SHC3	1.75923	siABH4#1 5nM up vs siC 5nM
C16orf46	1.75953	siABH4#1 5nM up vs siC 5nM
SNORA12	1.75999	siABH4#1 5nM up vs siC 5nM
PEX12	1.76139	siABH4#1 5nM up vs siC 5nM
HNRNPA3P1	1.76215	siABH4#1 5nM up vs siC 5nM
CKB	1.76232	siABH4#1 5nM up vs siC 5nM
COL4A5	1.76285	siABH4#1 5nM up vs siC 5nM
GALNT1	1.76299	siABH4#1 5nM up vs siC 5nM
ZC3H6	1.76299	siABH4#1 5nM up vs siC 5nM
FBXO44	1.76373	siABH4#1 5nM up vs siC 5nM
BAGE4	1.76376	siABH4#1 5nM up vs siC 5nM
LOC101927843	1.76489	siABH4#1 5nM up vs siC 5nM
C15orf52	1.76568	siABH4#1 5nM up vs siC 5nM
HOXC13	1.76633	siABH4#1 5nM up vs siC 5nM
KMO	1.76835	siABH4#1 5nM up vs siC 5nM
MDGA1	1.76874	siABH4#1 5nM up vs siC 5nM
DNER	1.7699	siABH4#1 5nM up vs siC 5nM
ANGEL1	1.77159	siABH4#1 5nM up vs siC 5nM
BCAS3	1.77183	siABH4#1 5nM up vs siC 5nM
MIR3974	1.77248	siABH4#1 5nM up vs siC 5nM
GSTM4	1.77295	siABH4#1 5nM up vs siC 5nM
IQSEC2	1.77387	siABH4#1 5nM up vs siC 5nM
PAPLN	1.77415	siABH4#1 5nM up vs siC 5nM
ZNF250	1.77419	siABH4#1 5nM up vs siC 5nM
CCDC69	1.77441	siABH4#1 5nM up vs siC 5nM
PCDHB2	1.7748	siABH4#1 5nM up vs siC 5nM
TRPM4	1.77507	siABH4#1 5nM up vs siC 5nM
SPOCK1	1.77557	siABH4#1 5nM up vs siC 5nM
MIR554	1.77585	siABH4#1 5nM up vs siC 5nM

TPM1	1.77665	siABH4#1 5nM up vs siC 5nM
RASA4B	1.77758	siABH4#1 5nM up vs siC 5nM
FHOD1	1.77878	siABH4#1 5nM up vs siC 5nM
KRCC1	1.7794	siABH4#1 5nM up vs siC 5nM
PLGLA	1.77977	siABH4#1 5nM up vs siC 5nM
TAS2R3	1.77988	siABH4#1 5nM up vs siC 5nM
LOC105373850	1.78011	siABH4#1 5nM up vs siC 5nM
NTPCR	1.78028	siABH4#1 5nM up vs siC 5nM
ZNF280D	1.78049	siABH4#1 5nM up vs siC 5nM
OR52N1	1.7809	siABH4#1 5nM up vs siC 5nM
HCCS	1.78092	siABH4#1 5nM up vs siC 5nM
MIR31	1.78121	siABH4#1 5nM up vs siC 5nM
SLCO5A1	1.78177	siABH4#1 5nM up vs siC 5nM
LINC00515	1.78318	siABH4#1 5nM up vs siC 5nM
LINC01473	1.78336	siABH4#1 5nM up vs siC 5nM
LINC01358	1.78372	siABH4#1 5nM up vs siC 5nM
C1orf228	1.78412	siABH4#1 5nM up vs siC 5nM
EPHA2	1.78464	siABH4#1 5nM up vs siC 5nM
SNORD82	1.78488	siABH4#1 5nM up vs siC 5nM
GRN	1.78612	siABH4#1 5nM up vs siC 5nM
SNORD116-12	1.78647	siABH4#1 5nM up vs siC 5nM
CCNG2	1.78803	siABH4#1 5nM up vs siC 5nM
LOC102723633	1.78828	siABH4#1 5nM up vs siC 5nM
PLEK2	1.78843	siABH4#1 5nM up vs siC 5nM
ARHGAP27	1.7885	siABH4#1 5nM up vs siC 5nM
CSRNP1	1.78859	siABH4#1 5nM up vs siC 5nM
KRTAP4-11	1.78935	siABH4#1 5nM up vs siC 5nM
IGKV1-6	1.78969	siABH4#1 5nM up vs siC 5nM
HLX	1.79159	siABH4#1 5nM up vs siC 5nM
KLHDC7A	1.79166	siABH4#1 5nM up vs siC 5nM
MCC	1.79167	siABH4#1 5nM up vs siC 5nM
INPP4B	1.79196	siABH4#1 5nM up vs siC 5nM

MFGE8	1.79317	siABH4#1 5nM up vs siC 5nM
TRBV6-4	1.79395	siABH4#1 5nM up vs siC 5nM
CPNE7	1.79456	siABH4#1 5nM up vs siC 5nM
DYRK1B	1.79553	siABH4#1 5nM up vs siC 5nM
CDK19	1.79608	siABH4#1 5nM up vs siC 5nM
POLH	1.7966	siABH4#1 5nM up vs siC 5nM
TMOD2	1.79705	siABH4#1 5nM up vs siC 5nM
LINC00969	1.79794	siABH4#1 5nM up vs siC 5nM
LACC1	1.7991	siABH4#1 5nM up vs siC 5nM
AQP7P3	1.80068	siABH4#1 5nM up vs siC 5nM
MXRA7	1.80095	siABH4#1 5nM up vs siC 5nM
MEX3C	1.80129	siABH4#1 5nM up vs siC 5nM
SMPD1	1.80189	siABH4#1 5nM up vs siC 5nM
GRIP1	1.8021	siABH4#1 5nM up vs siC 5nM
TRAJ17	1.80225	siABH4#1 5nM up vs siC 5nM
SNORD116-29	1.80352	siABH4#1 5nM up vs siC 5nM
NINJ1	1.80783	siABH4#1 5nM up vs siC 5nM
GREB1	1.80814	siABH4#1 5nM up vs siC 5nM
PON1	1.80985	siABH4#1 5nM up vs siC 5nM
PEAR1	1.81101	siABH4#1 5nM up vs siC 5nM
SLC7A2	1.81158	siABH4#1 5nM up vs siC 5nM
TBC1D7	1.81186	siABH4#1 5nM up vs siC 5nM
RETSAT	1.81224	siABH4#1 5nM up vs siC 5nM
PTPN21	1.81288	siABH4#1 5nM up vs siC 5nM
PTPRE	1.81306	siABH4#1 5nM up vs siC 5nM
OR5I1	1.81435	siABH4#1 5nM up vs siC 5nM
TNIP1	1.81462	siABH4#1 5nM up vs siC 5nM
TLL1	1.81472	siABH4#1 5nM up vs siC 5nM
IFI16	1.81502	siABH4#1 5nM up vs siC 5nM
LOC145474	1.81594	siABH4#1 5nM up vs siC 5nM
GADD45A	1.81615	siABH4#1 5nM up vs siC 5nM
LOC105370503	1.81626	siABH4#1 5nM up vs siC 5nM

SERTAD1	1.81638	siABH4#1 5nM up vs siC 5nM
TRBV10-2	1.81639	siABH4#1 5nM up vs siC 5nM
CHMP4C	1.81769	siABH4#1 5nM up vs siC 5nM
KCNK6	1.81811	siABH4#1 5nM up vs siC 5nM
LOC101927605	1.81827	siABH4#1 5nM up vs siC 5nM
METAP1D	1.81874	siABH4#1 5nM up vs siC 5nM
CCDC80	1.81911	siABH4#1 5nM up vs siC 5nM
SLC2A4RG	1.8207	siABH4#1 5nM up vs siC 5nM
RBMS3-AS3	1.82127	siABH4#1 5nM up vs siC 5nM
CD2AP	1.82171	siABH4#1 5nM up vs siC 5nM
SEMA4G	1.82181	siABH4#1 5nM up vs siC 5nM
LINC01060	1.82207	siABH4#1 5nM up vs siC 5nM
PPBP	1.82257	siABH4#1 5nM up vs siC 5nM
CACNA1D	1.82267	siABH4#1 5nM up vs siC 5nM
CFHR3	1.82529	siABH4#1 5nM up vs siC 5nM
ASIC3	1.82574	siABH4#1 5nM up vs siC 5nM
HSBP1L1	1.82621	siABH4#1 5nM up vs siC 5nM
GRIN2C	1.82688	siABH4#1 5nM up vs siC 5nM
PYGL	1.82735	siABH4#1 5nM up vs siC 5nM
INPPL1	1.82748	siABH4#1 5nM up vs siC 5nM
IGDCC4	1.82825	siABH4#1 5nM up vs siC 5nM
PI4K2A	1.82892	siABH4#1 5nM up vs siC 5nM
FGF2	1.82998	siABH4#1 5nM up vs siC 5nM
CCDC113	1.83027	siABH4#1 5nM up vs siC 5nM
FGD6	1.83103	siABH4#1 5nM up vs siC 5nM
UCN2	1.83213	siABH4#1 5nM up vs siC 5nM
SPHK1	1.83214	siABH4#1 5nM up vs siC 5nM
CLTCL1	1.8326	siABH4#1 5nM up vs siC 5nM
PSORS1C1	1.83336	siABH4#1 5nM up vs siC 5nM
CITED4	1.83394	siABH4#1 5nM up vs siC 5nM
FOSB	1.83517	siABH4#1 5nM up vs siC 5nM
DNAH10OS	1.83543	siABH4#1 5nM up vs siC 5nM

CLCN5	1.83554	siABH4#1 5nM up vs siC 5nM
CTGF	1.83708	siABH4#1 5nM up vs siC 5nM
LOC102724434	1.83774	siABH4#1 5nM up vs siC 5nM
POLD4	1.83831	siABH4#1 5nM up vs siC 5nM
PROS1	1.83837	siABH4#1 5nM up vs siC 5nM
ALOXE3	1.83898	siABH4#1 5nM up vs siC 5nM
CDRT1	1.8396	siABH4#1 5nM up vs siC 5nM
GAN	1.83996	siABH4#1 5nM up vs siC 5nM
TRAF4	1.84044	siABH4#1 5nM up vs siC 5nM
GOLGA6L4	1.84056	siABH4#1 5nM up vs siC 5nM
LGSN	1.84086	siABH4#1 5nM up vs siC 5nM
FRMD6	1.84227	siABH4#1 5nM up vs siC 5nM
SIAE	1.84382	siABH4#1 5nM up vs siC 5nM
GPR180	1.84485	siABH4#1 5nM up vs siC 5nM
LOC100507291	1.8455	siABH4#1 5nM up vs siC 5nM
SNAR-C4	1.84571	siABH4#1 5nM up vs siC 5nM
SNAR-C3	1.84571	siABH4#1 5nM up vs siC 5nM
ZCCHC24	1.84615	siABH4#1 5nM up vs siC 5nM
LOC100506730	1.84651	siABH4#1 5nM up vs siC 5nM
DFNB31	1.84748	siABH4#1 5nM up vs siC 5nM
FAM91A1	1.8475	siABH4#1 5nM up vs siC 5nM
HPX	1.84929	siABH4#1 5nM up vs siC 5nM
P4HA3	1.84975	siABH4#1 5nM up vs siC 5nM
LRRC37A3	1.85007	siABH4#1 5nM up vs siC 5nM
SNAR-C2	1.85196	siABH4#1 5nM up vs siC 5nM
SNAR-C2	1.85196	siABH4#1 5nM up vs siC 5nM
SNAR-C2	1.85196	siABH4#1 5nM up vs siC 5nM
SYT13	1.85198	siABH4#1 5nM up vs siC 5nM
ITGAX	1.85237	siABH4#1 5nM up vs siC 5nM
USP50	1.8534	siABH4#1 5nM up vs siC 5nM
TGFBR1	1.85392	siABH4#1 5nM up vs siC 5nM
FGF12	1.85509	siABH4#1 5nM up vs siC 5nM

KCNQ3	1.85523	siABH4#1 5nM up vs siC 5nM
AK7	1.85533	siABH4#1 5nM up vs siC 5nM
LOC105370765	1.85533	siABH4#1 5nM up vs siC 5nM
GSTM2	1.85596	siABH4#1 5nM up vs siC 5nM
MIR377	1.85633	siABH4#1 5nM up vs siC 5nM
SNORD32B	1.85642	siABH4#1 5nM up vs siC 5nM
PLEKHM1P	1.85665	siABH4#1 5nM up vs siC 5nM
LINC00842	1.85729	siABH4#1 5nM up vs siC 5nM
ITGB3	1.8578	siABH4#1 5nM up vs siC 5nM
WIPI1	1.85888	siABH4#1 5nM up vs siC 5nM
CASP3	1.85996	siABH4#1 5nM up vs siC 5nM
CLHC1	1.86079	siABH4#1 5nM up vs siC 5nM
PMEPA1	1.86115	siABH4#1 5nM up vs siC 5nM
FAM86HP	1.86257	siABH4#1 5nM up vs siC 5nM
TOB2P1	1.86294	siABH4#1 5nM up vs siC 5nM
SLC4A5	1.86333	siABH4#1 5nM up vs siC 5nM
OR7E14P	1.86339	siABH4#1 5nM up vs siC 5nM
SHC3	1.86371	siABH4#1 5nM up vs siC 5nM
FADS3	1.86423	siABH4#1 5nM up vs siC 5nM
KDM4B	1.86444	siABH4#1 5nM up vs siC 5nM
OLFML3	1.86528	siABH4#1 5nM up vs siC 5nM
DNAJC28	1.86544	siABH4#1 5nM up vs siC 5nM
DOCK2	1.86588	siABH4#1 5nM up vs siC 5nM
UGT2B11	1.86692	siABH4#1 5nM up vs siC 5nM
LOC101929713	1.86727	siABH4#1 5nM up vs siC 5nM
LOC105378179	1.86877	siABH4#1 5nM up vs siC 5nM
CCL2	1.86993	siABH4#1 5nM up vs siC 5nM
CACNA1H	1.87047	siABH4#1 5nM up vs siC 5nM
FAM214A	1.87054	siABH4#1 5nM up vs siC 5nM
EFR3B	1.87067	siABH4#1 5nM up vs siC 5nM
ZNF337	1.87112	siABH4#1 5nM up vs siC 5nM
LOC100507507	1.87113	siABH4#1 5nM up vs siC 5nM

SCARNA4	1.8718	siABH4#1 5nM up vs siC 5nM
KLC1	1.8721	siABH4#1 5nM up vs siC 5nM
MIR23B	1.87216	siABH4#1 5nM up vs siC 5nM
PLSCR4	1.87383	siABH4#1 5nM up vs siC 5nM
BAAT	1.87548	siABH4#1 5nM up vs siC 5nM
PAK3	1.87739	siABH4#1 5nM up vs siC 5nM
NLGN3	1.87748	siABH4#1 5nM up vs siC 5nM
PGM5P2	1.87826	siABH4#1 5nM up vs siC 5nM
TAS2R9	1.87833	siABH4#1 5nM up vs siC 5nM
ANXA4	1.87972	siABH4#1 5nM up vs siC 5nM
RASGRF2	1.87987	siABH4#1 5nM up vs siC 5nM
FAM84B	1.87995	siABH4#1 5nM up vs siC 5nM
TPM2	1.88016	siABH4#1 5nM up vs siC 5nM
CEP295NL	1.88043	siABH4#1 5nM up vs siC 5nM
SNAR-B2	1.88161	siABH4#1 5nM up vs siC 5nM
SNAR-B2	1.88161	siABH4#1 5nM up vs siC 5nM
TBC1D2B	1.882	siABH4#1 5nM up vs siC 5nM
KDM6B	1.88355	siABH4#1 5nM up vs siC 5nM
TMEM47	1.88518	siABH4#1 5nM up vs siC 5nM
ZC3H12A	1.88547	siABH4#1 5nM up vs siC 5nM
CTSF	1.88669	siABH4#1 5nM up vs siC 5nM
TRIAP1	1.88669	siABH4#1 5nM up vs siC 5nM
VCAN	1.8873	siABH4#1 5nM up vs siC 5nM
MEGF8	1.88883	siABH4#1 5nM up vs siC 5nM
ZNF219	1.88953	siABH4#1 5nM up vs siC 5nM
NCF2	1.89066	siABH4#1 5nM up vs siC 5nM
MUC3A	1.89095	siABH4#1 5nM up vs siC 5nM
SNORA64	1.89345	siABH4#1 5nM up vs siC 5nM
STX5	1.8935	siABH4#1 5nM up vs siC 5nM
ARRDC1	1.89419	siABH4#1 5nM up vs siC 5nM
PGAP3	1.89501	siABH4#1 5nM up vs siC 5nM
C7orf60	1.89559	siABH4#1 5nM up vs siC 5nM

MIR331	1.8956	siABH4#1 5nM up vs siC 5nM
PGM5-AS1	1.897	siABH4#1 5nM up vs siC 5nM
RNF121	1.89717	siABH4#1 5nM up vs siC 5nM
PYCARD	1.8991	siABH4#1 5nM up vs siC 5nM
MSC	1.89924	siABH4#1 5nM up vs siC 5nM
THBD	1.89926	siABH4#1 5nM up vs siC 5nM
MIR4645	1.89982	siABH4#1 5nM up vs siC 5nM
IGKV1-9	1.90215	siABH4#1 5nM up vs siC 5nM
CGB	1.90227	siABH4#1 5nM up vs siC 5nM
MIR581	1.9051	siABH4#1 5nM up vs siC 5nM
SNORA84	1.90629	siABH4#1 5nM up vs siC 5nM
ANKRD20A11P	1.90723	siABH4#1 5nM up vs siC 5nM
SLFN5	1.90727	siABH4#1 5nM up vs siC 5nM
ZNF493	1.91043	siABH4#1 5nM up vs siC 5nM
MIR4752	1.91125	siABH4#1 5nM up vs siC 5nM
CCDC30	1.91152	siABH4#1 5nM up vs siC 5nM
SNORD113-3	1.9126	siABH4#1 5nM up vs siC 5nM
RRM2B	1.91388	siABH4#1 5nM up vs siC 5nM
SNORA49	1.91542	siABH4#1 5nM up vs siC 5nM
GRIP1	1.91571	siABH4#1 5nM up vs siC 5nM
LOC105377283	1.91574	siABH4#1 5nM up vs siC 5nM
ZNF79	1.91594	siABH4#1 5nM up vs siC 5nM
KLF9	1.91603	siABH4#1 5nM up vs siC 5nM
FUCA1	1.91642	siABH4#1 5nM up vs siC 5nM
RTN4RL1	1.91873	siABH4#1 5nM up vs siC 5nM
SNORD116-27	1.92056	siABH4#1 5nM up vs siC 5nM
SNORD6	1.92102	siABH4#1 5nM up vs siC 5nM
ITGA11	1.92225	siABH4#1 5nM up vs siC 5nM
SLFNL1-AS1	1.92474	siABH4#1 5nM up vs siC 5nM
APOBEC3C	1.92534	siABH4#1 5nM up vs siC 5nM
CYP4V2	1.92756	siABH4#1 5nM up vs siC 5nM
SNAR-E	1.92767	siABH4#1 5nM up vs siC 5nM

CCDC191	1.92797	siABH4#1 5nM up vs siC 5nM
PKD1L2	1.92798	siABH4#1 5nM up vs siC 5nM
LOC105376235	1.93045	siABH4#1 5nM up vs siC 5nM
OR4N2	1.93229	siABH4#1 5nM up vs siC 5nM
NMRK1	1.93249	siABH4#1 5nM up vs siC 5nM
FHIT	1.9333	siABH4#1 5nM up vs siC 5nM
C1S	1.93606	siABH4#1 5nM up vs siC 5nM
LOC105372391	1.93621	siABH4#1 5nM up vs siC 5nM
KRT33A	1.93735	siABH4#1 5nM up vs siC 5nM
GRAMD1A	1.93788	siABH4#1 5nM up vs siC 5nM
DHRS1	1.93834	siABH4#1 5nM up vs siC 5nM
VDR	1.93916	siABH4#1 5nM up vs siC 5nM
GABRG3	1.93948	siABH4#1 5nM up vs siC 5nM
CFL1P1	1.94144	siABH4#1 5nM up vs siC 5nM
TIGAR	1.94177	siABH4#1 5nM up vs siC 5nM
IL18BP	1.94213	siABH4#1 5nM up vs siC 5nM
LOC101927552	1.9423	siABH4#1 5nM up vs siC 5nM
LOC102723846	1.94415	siABH4#1 5nM up vs siC 5nM
LOC105374674	1.94449	siABH4#1 5nM up vs siC 5nM
LINC01137	1.94463	siABH4#1 5nM up vs siC 5nM
UGT2B7	1.9463	siABH4#1 5nM up vs siC 5nM
ZDBF2	1.94644	siABH4#1 5nM up vs siC 5nM
ID1	1.9469	siABH4#1 5nM up vs siC 5nM
ITGB4	1.94812	siABH4#1 5nM up vs siC 5nM
PLA2R1	1.95012	siABH4#1 5nM up vs siC 5nM
C10orf11	1.95217	siABH4#1 5nM up vs siC 5nM
TMC7	1.95276	siABH4#1 5nM up vs siC 5nM
LOC154761	1.95411	siABH4#1 5nM up vs siC 5nM
NEO1	1.95541	siABH4#1 5nM up vs siC 5nM
BEAN1	1.9565	siABH4#1 5nM up vs siC 5nM
SNORD13P3	1.95692	siABH4#1 5nM up vs siC 5nM
CORO2B	1.9594	siABH4#1 5nM up vs siC 5nM

COL4A1	1.95944	siABH4#1 5nM up vs siC 5nM
LINC00685	1.96047	siABH4#1 5nM up vs siC 5nM
LINC00685	1.96047	siABH4#1 5nM up vs siC 5nM
GPC6	1.96101	siABH4#1 5nM up vs siC 5nM
PARD6G	1.96188	siABH4#1 5nM up vs siC 5nM
MRC2	1.96392	siABH4#1 5nM up vs siC 5nM
GCH1	1.96416	siABH4#1 5nM up vs siC 5nM
MIR4653	1.96565	siABH4#1 5nM up vs siC 5nM
ZDHHC20	1.96608	siABH4#1 5nM up vs siC 5nM
CYB5D2	1.96656	siABH4#1 5nM up vs siC 5nM
SNAR-D	1.96672	siABH4#1 5nM up vs siC 5nM
MIR379	1.96743	siABH4#1 5nM up vs siC 5nM
SNORD113-1	1.96769	siABH4#1 5nM up vs siC 5nM
SNORA22	1.96846	siABH4#1 5nM up vs siC 5nM
TMEM132A	1.96849	siABH4#1 5nM up vs siC 5nM
GAMT	1.97136	siABH4#1 5nM up vs siC 5nM
TTC9	1.97424	siABH4#1 5nM up vs siC 5nM
RHOD	1.9747	siABH4#1 5nM up vs siC 5nM
CES2	1.97571	siABH4#1 5nM up vs siC 5nM
RDH10	1.97575	siABH4#1 5nM up vs siC 5nM
ANKRA2	1.9766	siABH4#1 5nM up vs siC 5nM
XPC	1.97724	siABH4#1 5nM up vs siC 5nM
TMEM19	1.97758	siABH4#1 5nM up vs siC 5nM
MIR4500HG	1.97944	siABH4#1 5nM up vs siC 5nM
TOP1P1	1.97959	siABH4#1 5nM up vs siC 5nM
HMGN2P46	1.98246	siABH4#1 5nM up vs siC 5nM
SMOC1	1.98293	siABH4#1 5nM up vs siC 5nM
LLGL1	1.98332	siABH4#1 5nM up vs siC 5nM
SOD2	1.98404	siABH4#1 5nM up vs siC 5nM
KIAA1644	1.98507	siABH4#1 5nM up vs siC 5nM
ZNF600	1.98695	siABH4#1 5nM up vs siC 5nM
LOC102724687	1.98895	siABH4#1 5nM up vs siC 5nM

RASA4	1.99037	siABH4#1 5nM up vs siC 5nM
MIA3	1.99074	siABH4#1 5nM up vs siC 5nM
ERO1B	1.99102	siABH4#1 5nM up vs siC 5nM
CGB2	1.99289	siABH4#1 5nM up vs siC 5nM
CHIC1	1.99392	siABH4#1 5nM up vs siC 5nM
MIR4481	1.99404	siABH4#1 5nM up vs siC 5nM
ATP7B	1.99545	siABH4#1 5nM up vs siC 5nM
B4GALT6	1.99752	siABH4#1 5nM up vs siC 5nM
C1orf101	2.00005	siABH4#1 5nM up vs siC 5nM
OR5D14	2.00005	siABH4#1 5nM up vs siC 5nM
ACTBL2	2.00228	siABH4#1 5nM up vs siC 5nM
MBNL2	2.00326	siABH4#1 5nM up vs siC 5nM
PLXNA3	2.00376	siABH4#1 5nM up vs siC 5nM
PS1TP4	2.0039	siABH4#1 5nM up vs siC 5nM
COL4A6	2.00417	siABH4#1 5nM up vs siC 5nM
SEZ6L2	2.00503	siABH4#1 5nM up vs siC 5nM
DNAH14	2.00576	siABH4#1 5nM up vs siC 5nM
BIN1	2.01134	siABH4#1 5nM up vs siC 5nM
TOM1	2.01182	siABH4#1 5nM up vs siC 5nM
TSSC2	2.01372	siABH4#1 5nM up vs siC 5nM
HGSNAT	2.0146	siABH4#1 5nM up vs siC 5nM
DHRS3	2.01646	siABH4#1 5nM up vs siC 5nM
MIR421	2.01682	siABH4#1 5nM up vs siC 5nM
ROGDI	2.0169	siABH4#1 5nM up vs siC 5nM
ARHGEF40	2.01803	siABH4#1 5nM up vs siC 5nM
C9orf9	2.0181	siABH4#1 5nM up vs siC 5nM
OR8H2	2.0184	siABH4#1 5nM up vs siC 5nM
LYN	2.01896	siABH4#1 5nM up vs siC 5nM
ZNF474	2.01982	siABH4#1 5nM up vs siC 5nM
CNFN	2.02013	siABH4#1 5nM up vs siC 5nM
CSAD	2.02095	siABH4#1 5nM up vs siC 5nM
MIR325	2.02107	siABH4#1 5nM up vs siC 5nM

HIST1H3G	2.02406	siABH4#1 5nM up vs siC 5nM
LOC441455	2.02551	siABH4#1 5nM up vs siC 5nM
MIR493	2.02708	siABH4#1 5nM up vs siC 5nM
BRD7P3	2.02708	siABH4#1 5nM up vs siC 5nM
HKDC1	2.02719	siABH4#1 5nM up vs siC 5nM
LOC101927120	2.03011	siABH4#1 5nM up vs siC 5nM
LOC389906	2.03115	siABH4#1 5nM up vs siC 5nM
NEFL	2.03148	siABH4#1 5nM up vs siC 5nM
SLC22A3	2.03261	siABH4#1 5nM up vs siC 5nM
NADSYN1	2.03286	siABH4#1 5nM up vs siC 5nM
NFKB2	2.03297	siABH4#1 5nM up vs siC 5nM
STX12	2.03867	siABH4#1 5nM up vs siC 5nM
HSD17B14	2.03958	siABH4#1 5nM up vs siC 5nM
LTBP2	2.03977	siABH4#1 5nM up vs siC 5nM
CYP7B1	2.04058	siABH4#1 5nM up vs siC 5nM
LOC105376895	2.0411	siABH4#1 5nM up vs siC 5nM
TMEM87B	2.04505	siABH4#1 5nM up vs siC 5nM
C4orf26	2.04505	siABH4#1 5nM up vs siC 5nM
PCDHB3	2.04515	siABH4#1 5nM up vs siC 5nM
LOC440173	2.04675	siABH4#1 5nM up vs siC 5nM
MIR889	2.04788	siABH4#1 5nM up vs siC 5nM
DDAH1	2.05269	siABH4#1 5nM up vs siC 5nM
FZD6	2.05288	siABH4#1 5nM up vs siC 5nM
GAA	2.0559	siABH4#1 5nM up vs siC 5nM
LCAT	2.05664	siABH4#1 5nM up vs siC 5nM
LOC105377265	2.05703	siABH4#1 5nM up vs siC 5nM
TSC22D2	2.05784	siABH4#1 5nM up vs siC 5nM
MIR628	2.05818	siABH4#1 5nM up vs siC 5nM
PWAR6	2.05877	siABH4#1 5nM up vs siC 5nM
ZNF763	2.05942	siABH4#1 5nM up vs siC 5nM
PM20D2	2.06299	siABH4#1 5nM up vs siC 5nM
FRAS1	2.06495	siABH4#1 5nM up vs siC 5nM

LTBP3	2.06976	siABH4#1 5nM up vs siC 5nM
MIR1305	2.06996	siABH4#1 5nM up vs siC 5nM
LOC105369301	2.07132	siABH4#1 5nM up vs siC 5nM
KRT19	2.0763	siABH4#1 5nM up vs siC 5nM
SLC9A7	2.0772	siABH4#1 5nM up vs siC 5nM
RAB12	2.07764	siABH4#1 5nM up vs siC 5nM
SDIM1	2.07841	siABH4#1 5nM up vs siC 5nM
AADAC	2.07864	siABH4#1 5nM up vs siC 5nM
GLS	2.08102	siABH4#1 5nM up vs siC 5nM
LINC00271	2.08468	siABH4#1 5nM up vs siC 5nM
HIST1H1T	2.08504	siABH4#1 5nM up vs siC 5nM
MIR4684	2.08566	siABH4#1 5nM up vs siC 5nM
BVES	2.08886	siABH4#1 5nM up vs siC 5nM
LRP4	2.09194	siABH4#1 5nM up vs siC 5nM
MYO1D	2.09391	siABH4#1 5nM up vs siC 5nM
ALDH3A1	2.0944	siABH4#1 5nM up vs siC 5nM
SNORD116-20	2.09554	siABH4#1 5nM up vs siC 5nM
PSG4	2.09679	siABH4#1 5nM up vs siC 5nM
ID3	2.09686	siABH4#1 5nM up vs siC 5nM
SLC22A5	2.09709	siABH4#1 5nM up vs siC 5nM
ABCC3	2.09727	siABH4#1 5nM up vs siC 5nM
TP53TG1	2.09896	siABH4#1 5nM up vs siC 5nM
ACSF2	2.10194	siABH4#1 5nM up vs siC 5nM
RPS6KA6	2.10291	siABH4#1 5nM up vs siC 5nM
LOC105373737	2.10382	siABH4#1 5nM up vs siC 5nM
SNORD114-17	2.11132	siABH4#1 5nM up vs siC 5nM
PLK3	2.11235	siABH4#1 5nM up vs siC 5nM
CCDC162P	2.11412	siABH4#1 5nM up vs siC 5nM
MBD5	2.1145	siABH4#1 5nM up vs siC 5nM
LOC102723769	2.12233	siABH4#1 5nM up vs siC 5nM
LOC105371267	2.12456	siABH4#1 5nM up vs siC 5nM
PWAR5	2.12518	siABH4#1 5nM up vs siC 5nM

TMEM68	2.12761	siABH4#1 5nM up vs siC 5nM
TMEM9B-AS1	2.12937	siABH4#1 5nM up vs siC 5nM
LOC105372415	2.12996	siABH4#1 5nM up vs siC 5nM
ATP13A2	2.13037	siABH4#1 5nM up vs siC 5nM
MIR491	2.13067	siABH4#1 5nM up vs siC 5nM
SERPINE2	2.13082	siABH4#1 5nM up vs siC 5nM
CMTM4	2.13151	siABH4#1 5nM up vs siC 5nM
SNORD114-21	2.13477	siABH4#1 5nM up vs siC 5nM
NFKBIZ	2.13592	siABH4#1 5nM up vs siC 5nM
BTBD19	2.13601	siABH4#1 5nM up vs siC 5nM
PCDHB6	2.13901	siABH4#1 5nM up vs siC 5nM
FAM223A	2.13986	siABH4#1 5nM up vs siC 5nM
AKR1B15	2.14025	siABH4#1 5nM up vs siC 5nM
FBXL17	2.14144	siABH4#1 5nM up vs siC 5nM
MIR487B	2.1423	siABH4#1 5nM up vs siC 5nM
LOC105370555	2.14557	siABH4#1 5nM up vs siC 5nM
PLXNB3	2.14638	siABH4#1 5nM up vs siC 5nM
KDM7A	2.14758	siABH4#1 5nM up vs siC 5nM
PTPRM	2.14773	siABH4#1 5nM up vs siC 5nM
LOC553103	2.15267	siABH4#1 5nM up vs siC 5nM
MMP25-AS1	2.16456	siABH4#1 5nM up vs siC 5nM
SNORA7B	2.1661	siABH4#1 5nM up vs siC 5nM
PRKAB2	2.16659	siABH4#1 5nM up vs siC 5nM
LINC00571	2.16952	siABH4#1 5nM up vs siC 5nM
FSTL3	2.17017	siABH4#1 5nM up vs siC 5nM
TNFRSF10B	2.17139	siABH4#1 5nM up vs siC 5nM
GBP2	2.17407	siABH4#1 5nM up vs siC 5nM
ZNF285	2.17577	siABH4#1 5nM up vs siC 5nM
PLGLB1	2.17597	siABH4#1 5nM up vs siC 5nM
STARD4	2.17864	siABH4#1 5nM up vs siC 5nM
TGFB1I1	2.17984	siABH4#1 5nM up vs siC 5nM
OR14A16	2.18047	siABH4#1 5nM up vs siC 5nM

PLXNB1	2.18439	siABH4#1 5nM up vs siC 5nM
TAS2R10	2.18573	siABH4#1 5nM up vs siC 5nM
BMP1	2.18639	siABH4#1 5nM up vs siC 5nM
SETD7	2.18726	siABH4#1 5nM up vs siC 5nM
ARSG	2.19093	siABH4#1 5nM up vs siC 5nM
CRYL1	2.1931	siABH4#1 5nM up vs siC 5nM
SNORA47	2.19585	siABH4#1 5nM up vs siC 5nM
TPD52L1	2.19672	siABH4#1 5nM up vs siC 5nM
DUX4L4	2.19805	siABH4#1 5nM up vs siC 5nM
PGM2L1	2.20195	siABH4#1 5nM up vs siC 5nM
TSPYL2	2.20218	siABH4#1 5nM up vs siC 5nM
SLC6A17	2.20248	siABH4#1 5nM up vs siC 5nM
NBEAP1	2.2092	siABH4#1 5nM up vs siC 5nM
LAMB2	2.21344	siABH4#1 5nM up vs siC 5nM
LINC01594	2.21349	siABH4#1 5nM up vs siC 5nM
BEND7	2.21443	siABH4#1 5nM up vs siC 5nM
PIK3IP1	2.21767	siABH4#1 5nM up vs siC 5nM
CDA	2.21841	siABH4#1 5nM up vs siC 5nM
PLXNB2	2.2265	siABH4#1 5nM up vs siC 5nM
STX17-AS1	2.22788	siABH4#1 5nM up vs siC 5nM
C1QTNF1	2.23178	siABH4#1 5nM up vs siC 5nM
NEAT1	2.23883	siABH4#1 5nM up vs siC 5nM
DRAM1	2.23971	siABH4#1 5nM up vs siC 5nM
PTPRU	2.24562	siABH4#1 5nM up vs siC 5nM
IRAK2	2.2476	siABH4#1 5nM up vs siC 5nM
RRS1-AS1	2.25128	siABH4#1 5nM up vs siC 5nM
SNORD60	2.25138	siABH4#1 5nM up vs siC 5nM
MIR181A2HG	2.25501	siABH4#1 5nM up vs siC 5nM
LYRM1	2.25971	siABH4#1 5nM up vs siC 5nM
NLRP1	2.26862	siABH4#1 5nM up vs siC 5nM
VSIG1	2.26877	siABH4#1 5nM up vs siC 5nM
TBC1D2	2.26888	siABH4#1 5nM up vs siC 5nM

SORCS2	2.26897	siABH4#1 5nM up vs siC 5nM
CXCL1	2.27334	siABH4#1 5nM up vs siC 5nM
TTC29	2.27343	siABH4#1 5nM up vs siC 5nM
C14orf28	2.27593	siABH4#1 5nM up vs siC 5nM
PNPO	2.27739	siABH4#1 5nM up vs siC 5nM
LOC101927400	2.27834	siABH4#1 5nM up vs siC 5nM
GBP1	2.28563	siABH4#1 5nM up vs siC 5nM
TIMD4	2.28736	siABH4#1 5nM up vs siC 5nM
LOC100128494	2.2879	siABH4#1 5nM up vs siC 5nM
C7orf31	2.29407	siABH4#1 5nM up vs siC 5nM
EPHB2	2.2946	siABH4#1 5nM up vs siC 5nM
SLC30A1	2.29497	siABH4#1 5nM up vs siC 5nM
SNORD116-28	2.29686	siABH4#1 5nM up vs siC 5nM
SLC16A4	2.3027	siABH4#1 5nM up vs siC 5nM
HGD	2.30448	siABH4#1 5nM up vs siC 5nM
PDGFA	2.30505	siABH4#1 5nM up vs siC 5nM
LOC102723989	2.30531	siABH4#1 5nM up vs siC 5nM
FLRT2	2.30722	siABH4#1 5nM up vs siC 5nM
MXD1	2.30808	siABH4#1 5nM up vs siC 5nM
HIST1H2BC	2.31077	siABH4#1 5nM up vs siC 5nM
KLHL24	2.31429	siABH4#1 5nM up vs siC 5nM
CXCL8	2.31599	siABH4#1 5nM up vs siC 5nM
TRIM8	2.31832	siABH4#1 5nM up vs siC 5nM
LOC101928767	2.32018	siABH4#1 5nM up vs siC 5nM
LAMA5	2.32067	siABH4#1 5nM up vs siC 5nM
MIR181B2	2.32138	siABH4#1 5nM up vs siC 5nM
CCDC162P	2.32371	siABH4#1 5nM up vs siC 5nM
LOC388282	2.32866	siABH4#1 5nM up vs siC 5nM
SLC43A2	2.32956	siABH4#1 5nM up vs siC 5nM
SNORD92	2.33229	siABH4#1 5nM up vs siC 5nM
C5orf49	2.33609	siABH4#1 5nM up vs siC 5nM
KCND1	2.33665	siABH4#1 5nM up vs siC 5nM

LOC100268168	2.33857	siABH4#1 5nM up vs siC 5nM
PARP3	2.33944	siABH4#1 5nM up vs siC 5nM
SNORD114-6	2.34056	siABH4#1 5nM up vs siC 5nM
RAP2C-AS1	2.3411	siABH4#1 5nM up vs siC 5nM
ELK3	2.3432	siABH4#1 5nM up vs siC 5nM
LINC01239	2.34406	siABH4#1 5nM up vs siC 5nM
GPR155	2.34922	siABH4#1 5nM up vs siC 5nM
LOC105371638	2.35147	siABH4#1 5nM up vs siC 5nM
TMEM27	2.35153	siABH4#1 5nM up vs siC 5nM
PITPNM1	2.35475	siABH4#1 5nM up vs siC 5nM
PTPRO	2.36048	siABH4#1 5nM up vs siC 5nM
SERPINE1	2.36481	siABH4#1 5nM up vs siC 5nM
HHLA3	2.37198	siABH4#1 5nM up vs siC 5nM
TRAJ14	2.37652	siABH4#1 5nM up vs siC 5nM
SNORD71	2.37717	siABH4#1 5nM up vs siC 5nM
NEDD4	2.37747	siABH4#1 5nM up vs siC 5nM
SLCO1B1	2.37772	siABH4#1 5nM up vs siC 5nM
SNORA11	2.38036	siABH4#1 5nM up vs siC 5nM
RASA4B	2.38357	siABH4#1 5nM up vs siC 5nM
TRANK1	2.38635	siABH4#1 5nM up vs siC 5nM
HTRA1	2.3957	siABH4#1 5nM up vs siC 5nM
STAT4	2.39918	siABH4#1 5nM up vs siC 5nM
THSD1	2.4079	siABH4#1 5nM up vs siC 5nM
IDUA	2.40983	siABH4#1 5nM up vs siC 5nM
TLR3	2.412	siABH4#1 5nM up vs siC 5nM
ICA1	2.41537	siABH4#1 5nM up vs siC 5nM
LOC105375320	2.41594	siABH4#1 5nM up vs siC 5nM
YPEL5	2.41787	siABH4#1 5nM up vs siC 5nM
ANXA10	2.42285	siABH4#1 5nM up vs siC 5nM
ALPK2	2.42475	siABH4#1 5nM up vs siC 5nM
GDPD1	2.42942	siABH4#1 5nM up vs siC 5nM
ZBED5-AS1	2.43732	siABH4#1 5nM up vs siC 5nM

TMOD1	2.43755	siABH4#1 5nM up vs siC 5nM
MIR3936	2.44807	siABH4#1 5nM up vs siC 5nM
SNORD109B	2.45056	siABH4#1 5nM up vs siC 5nM
SNORD109B	2.45056	siABH4#1 5nM up vs siC 5nM
FAM228B	2.45574	siABH4#1 5nM up vs siC 5nM
GCNT4	2.46367	siABH4#1 5nM up vs siC 5nM
ERN1	2.46858	siABH4#1 5nM up vs siC 5nM
JUNB	2.47029	siABH4#1 5nM up vs siC 5nM
SNORD116-2	2.4753	siABH4#1 5nM up vs siC 5nM
SNORD116-6	2.4753	siABH4#1 5nM up vs siC 5nM
EBI3	2.47583	siABH4#1 5nM up vs siC 5nM
SNORD114-15	2.48063	siABH4#1 5nM up vs siC 5nM
REEP6	2.48191	siABH4#1 5nM up vs siC 5nM
LOC105379581	2.48264	siABH4#1 5nM up vs siC 5nM
ACYP2	2.48413	siABH4#1 5nM up vs siC 5nM
OLFM2	2.48678	siABH4#1 5nM up vs siC 5nM
RASA4B	2.49758	siABH4#1 5nM up vs siC 5nM
PDCD1LG2	2.49954	siABH4#1 5nM up vs siC 5nM
IL32	2.50382	siABH4#1 5nM up vs siC 5nM
LINC00488	2.50747	siABH4#1 5nM up vs siC 5nM
KLLN	2.50874	siABH4#1 5nM up vs siC 5nM
EPS8L2	2.51118	siABH4#1 5nM up vs siC 5nM
FNDC3A	2.51517	siABH4#1 5nM up vs siC 5nM
TMEM63B	2.52348	siABH4#1 5nM up vs siC 5nM
LINC00412	2.52844	siABH4#1 5nM up vs siC 5nM
LOC105370672	2.52859	siABH4#1 5nM up vs siC 5nM
SNORA14A	2.53262	siABH4#1 5nM up vs siC 5nM
MIR31HG	2.54569	siABH4#1 5nM up vs siC 5nM
RASGRP1	2.55562	siABH4#1 5nM up vs siC 5nM
SPARC	2.55942	siABH4#1 5nM up vs siC 5nM
VLDLR	2.56438	siABH4#1 5nM up vs siC 5nM
ARSD	2.56572	siABH4#1 5nM up vs siC 5nM

DPYSL4	2.56613	siABH4#1 5nM up vs siC 5nM
RHBDF2	2.56713	siABH4#1 5nM up vs siC 5nM
TRPV1	2.56908	siABH4#1 5nM up vs siC 5nM
TNFAIP3	2.57276	siABH4#1 5nM up vs siC 5nM
RBP4	2.58316	siABH4#1 5nM up vs siC 5nM
RNU5B-1	2.58395	siABH4#1 5nM up vs siC 5nM
RAB3B	2.58501	siABH4#1 5nM up vs siC 5nM
GAS6-AS1	2.5879	siABH4#1 5nM up vs siC 5nM
LMBRD1	2.58914	siABH4#1 5nM up vs siC 5nM
FBXO32	2.59031	siABH4#1 5nM up vs siC 5nM
TBC1D7	2.60606	siABH4#1 5nM up vs siC 5nM
SEC31B	2.60651	siABH4#1 5nM up vs siC 5nM
EDEM2	2.6109	siABH4#1 5nM up vs siC 5nM
ABCA1	2.6172	siABH4#1 5nM up vs siC 5nM
FHL2	2.6176	siABH4#1 5nM up vs siC 5nM
CYGB	2.61827	siABH4#1 5nM up vs siC 5nM
PODXL	2.62514	siABH4#1 5nM up vs siC 5nM
ADAMTS7	2.6256	siABH4#1 5nM up vs siC 5nM
MYO6	2.62662	siABH4#1 5nM up vs siC 5nM
CDKN1A	2.62948	siABH4#1 5nM up vs siC 5nM
MEG3	2.62977	siABH4#1 5nM up vs siC 5nM
CYP3A5	2.63316	siABH4#1 5nM up vs siC 5nM
RBMS3	2.63878	siABH4#1 5nM up vs siC 5nM
AGMO	2.64282	siABH4#1 5nM up vs siC 5nM
LOC101927372	2.64306	siABH4#1 5nM up vs siC 5nM
SNORD116-30	2.65231	siABH4#1 5nM up vs siC 5nM
TSPAN2	2.6672	siABH4#1 5nM up vs siC 5nM
GBP2	2.66742	siABH4#1 5nM up vs siC 5nM
BCRP3	2.67978	siABH4#1 5nM up vs siC 5nM
LINC01583	2.68512	siABH4#1 5nM up vs siC 5nM
TNFRSF10D	2.68882	siABH4#1 5nM up vs siC 5nM
CAPN3	2.69043	siABH4#1 5nM up vs siC 5nM

SLC9A7	2.72135	siABH4#1 5nM up vs siC 5nM
LINC01021	2.73381	siABH4#1 5nM up vs siC 5nM
ZBTB41	2.73726	siABH4#1 5nM up vs siC 5nM
LOC105370924	2.75481	siABH4#1 5nM up vs siC 5nM
FAS	2.75799	siABH4#1 5nM up vs siC 5nM
MIR1185-1	2.76462	siABH4#1 5nM up vs siC 5nM
FBN2	2.77076	siABH4#1 5nM up vs siC 5nM
KCCAT198	2.77419	siABH4#1 5nM up vs siC 5nM
LOC105369340	2.77472	siABH4#1 5nM up vs siC 5nM
SNORD116-25	2.78114	siABH4#1 5nM up vs siC 5nM
ST8SIA6	2.79341	siABH4#1 5nM up vs siC 5nM
MIR154	2.79868	siABH4#1 5nM up vs siC 5nM
SNORD114-2	2.80233	siABH4#1 5nM up vs siC 5nM
SNORD116-3	2.80687	siABH4#1 5nM up vs siC 5nM
SNORD116-8	2.80687	siABH4#1 5nM up vs siC 5nM
SNORD116-3	2.80687	siABH4#1 5nM up vs siC 5nM
SGPP1	2.80792	siABH4#1 5nM up vs siC 5nM
LRP1	2.80917	siABH4#1 5nM up vs siC 5nM
MMP25-AS1	2.81718	siABH4#1 5nM up vs siC 5nM
PLEKHG1	2.82241	siABH4#1 5nM up vs siC 5nM
TP53I3	2.83142	siABH4#1 5nM up vs siC 5nM
PDZK1IP1	2.8396	siABH4#1 5nM up vs siC 5nM
THBS3	2.84072	siABH4#1 5nM up vs siC 5nM
CCDC146	2.85218	siABH4#1 5nM up vs siC 5nM
MIR181A2	2.85255	siABH4#1 5nM up vs siC 5nM
SNORD116-23	2.85486	siABH4#1 5nM up vs siC 5nM
SNORD105B	2.856	siABH4#1 5nM up vs siC 5nM
ENPP4	2.85628	siABH4#1 5nM up vs siC 5nM
PHLDA3	2.8678	siABH4#1 5nM up vs siC 5nM
CMBL	2.88293	siABH4#1 5nM up vs siC 5nM
SPATA17	2.89132	siABH4#1 5nM up vs siC 5nM
SNORD113-7	2.89339	siABH4#1 5nM up vs siC 5nM

PDLIM7	2.92013	siABH4#1 5nM up vs siC 5nM
SNORD41	2.92132	siABH4#1 5nM up vs siC 5nM
PAPPA-AS2	2.9336	siABH4#1 5nM up vs siC 5nM
PCDHB14	2.94725	siABH4#1 5nM up vs siC 5nM
YPEL2	2.95041	siABH4#1 5nM up vs siC 5nM
CASC19	2.95537	siABH4#1 5nM up vs siC 5nM
SNORD46	2.96724	siABH4#1 5nM up vs siC 5nM
RND1	2.96891	siABH4#1 5nM up vs siC 5nM
SESN2	2.99228	siABH4#1 5nM up vs siC 5nM
SNORD116-5	3.03109	siABH4#1 5nM up vs siC 5nM
SNORD116-5	3.03109	siABH4#1 5nM up vs siC 5nM
TMEM150C	3.03183	siABH4#1 5nM up vs siC 5nM
LOC105378088	3.04317	siABH4#1 5nM up vs siC 5nM
SNORD113-8	3.0575	siABH4#1 5nM up vs siC 5nM
IL1A	3.06036	siABH4#1 5nM up vs siC 5nM
SNORD116-24	3.0777	siABH4#1 5nM up vs siC 5nM
CYFIP2	3.08591	siABH4#1 5nM up vs siC 5nM
MR1	3.10603	siABH4#1 5nM up vs siC 5nM
LOC105369301	3.11414	siABH4#1 5nM up vs siC 5nM
PCED1B	3.1165	siABH4#1 5nM up vs siC 5nM
SNORD114-28	3.11747	siABH4#1 5nM up vs siC 5nM
CASC9	3.11813	siABH4#1 5nM up vs siC 5nM
CLYBL	3.12002	siABH4#1 5nM up vs siC 5nM
IFNA1	3.13071	siABH4#1 5nM up vs siC 5nM
LOC105376382	3.16776	siABH4#1 5nM up vs siC 5nM
SLC39A10	3.18426	siABH4#1 5nM up vs siC 5nM
DSC3	3.18689	siABH4#1 5nM up vs siC 5nM
OR5M1	3.18871	siABH4#1 5nM up vs siC 5nM
SNORD116-11	3.22023	siABH4#1 5nM up vs siC 5nM
EDA2R	3.26547	siABH4#1 5nM up vs siC 5nM
MIR323A	3.26615	siABH4#1 5nM up vs siC 5nM
PLA2G4C	3.30901	siABH4#1 5nM up vs siC 5nM

SNORD116-26	3.32121	siABH4#1 5nM up vs siC 5nM
ASS1	3.362	siABH4#1 5nM up vs siC 5nM
SNORD114-12	3.39013	siABH4#1 5nM up vs siC 5nM
ADGRF4	3.39686	siABH4#1 5nM up vs siC 5nM
LOC105371809	3.40562	siABH4#1 5nM up vs siC 5nM
LOC101927501	3.42892	siABH4#1 5nM up vs siC 5nM
MDM2	3.4292	siABH4#1 5nM up vs siC 5nM
DCBLD1	3.43733	siABH4#1 5nM up vs siC 5nM
CGB8	3.4531	siABH4#1 5nM up vs siC 5nM
PBLD	3.47836	siABH4#1 5nM up vs siC 5nM
FSTL1	3.48767	siABH4#1 5nM up vs siC 5nM
RASA4B	3.50696	siABH4#1 5nM up vs siC 5nM
SNORD114-22	3.53205	siABH4#1 5nM up vs siC 5nM
DSC2	3.54766	siABH4#1 5nM up vs siC 5nM
ZNF385A	3.55085	siABH4#1 5nM up vs siC 5nM
ANGPTL4	3.56149	siABH4#1 5nM up vs siC 5nM
HSPG2	3.59484	siABH4#1 5nM up vs siC 5nM
ANXA8L1	3.61235	siABH4#1 5nM up vs siC 5nM
TRAM1	3.61418	siABH4#1 5nM up vs siC 5nM
SLC46A3	3.63481	siABH4#1 5nM up vs siC 5nM
WDR63	3.65215	siABH4#1 5nM up vs siC 5nM
LINC01594	3.68889	siABH4#1 5nM up vs siC 5nM
FDXR	3.7061	siABH4#1 5nM up vs siC 5nM
SNORD114-23	3.81367	siABH4#1 5nM up vs siC 5nM
ITGA2	3.81821	siABH4#1 5nM up vs siC 5nM
MYL9	3.82258	siABH4#1 5nM up vs siC 5nM
SNORD114-1	3.82508	siABH4#1 5nM up vs siC 5nM
SNORD116-13	3.84809	siABH4#1 5nM up vs siC 5nM
LOC105372424	3.92118	siABH4#1 5nM up vs siC 5nM
PHLDA3	3.93941	siABH4#1 5nM up vs siC 5nM
C3	4.0194	siABH4#1 5nM up vs siC 5nM
NAP1L3	4.06258	siABH4#1 5nM up vs siC 5nM

DGKA	4.23702	siABH4#1 5nM up vs siC 5nM
SNORD114-9	4.26023	siABH4#1 5nM up vs siC 5nM
EFNA1	4.33335	siABH4#1 5nM up vs siC 5nM
SPATA18	4.37433	siABH4#1 5nM up vs siC 5nM
GBP3	4.43395	siABH4#1 5nM up vs siC 5nM
IFNE	4.47749	siABH4#1 5nM up vs siC 5nM
RRAD	4.48161	siABH4#1 5nM up vs siC 5nM
TAGLN	4.50082	siABH4#1 5nM up vs siC 5nM
SESN1	4.51461	siABH4#1 5nM up vs siC 5nM
CTSO	4.52223	siABH4#1 5nM up vs siC 5nM
ZMAT3	4.52898	siABH4#1 5nM up vs siC 5nM
SNORD114-13	4.60973	siABH4#1 5nM up vs siC 5nM
ACTA2	4.62643	siABH4#1 5nM up vs siC 5nM
CDK14	4.65928	siABH4#1 5nM up vs siC 5nM
SULF2	4.72898	siABH4#1 5nM up vs siC 5nM
SNORD114-11	4.73489	siABH4#1 5nM up vs siC 5nM
ICAM1	4.74651	siABH4#1 5nM up vs siC 5nM
SNORD116-1	4.86127	siABH4#1 5nM up vs siC 5nM
ITGB6	4.88919	siABH4#1 5nM up vs siC 5nM
ANXA8	4.90204	siABH4#1 5nM up vs siC 5nM
BTG2	4.91741	siABH4#1 5nM up vs siC 5nM
ANXA8	4.96383	siABH4#1 5nM up vs siC 5nM
SNORD114-3	4.98013	siABH4#1 5nM up vs siC 5nM
LOC105371809	5.13711	siABH4#1 5nM up vs siC 5nM
LOC100129550	5.19455	siABH4#1 5nM up vs siC 5nM
SNORD114-26	5.54161	siABH4#1 5nM up vs siC 5nM
ABCA12	5.64304	siABH4#1 5nM up vs siC 5nM
SNORD114-25	5.67866	siABH4#1 5nM up vs siC 5nM
LOC105375451	5.85868	siABH4#1 5nM up vs siC 5nM
SNORD113-9	6.27427	siABH4#1 5nM up vs siC 5nM
ACER2	6.46743	siABH4#1 5nM up vs siC 5nM
LOC105374342	8.12821	siABH4#1 5nM up vs siC 5nM

TP53INP1	8.38736	siABH4#1 5nM up vs siC 5nM

Supplementary table 3. Gene ontology enrichment analysis of 2011 genes downregulated by ALKBH4 knockdown

**BioPlanet_2019**	
**Term**	**Adjusted P-value**
Cell cycle	4.E-78
DNA replication	1.E-53
M phase pathway	5.E-36
Chromosome maintenance	4.E-30
G2/M checkpoints	1.E-22
Activation of the pre-replicative complex	3.E-20
Mitotic G1-G1/S phases	1.E-19
DNA strand elongation	1.E-19
S phase	1.E-17
Telomere maintenance	2.E-17
Meiosis	8.E-17
Cell cycle checkpoints	1.E-16
Deposition of new CENP-A-containing nucleosomes at the centromere	1.E-16
Interactions of HIV Vpr protein with host cellular proteins	6.E-16
Meiotic recombination	7.E-16
Aurora B signaling	3.E-14
Mitotic G2-G2/M phases	2.E-14
G1 to S cell cycle control	3.E-14
Mitotic prometaphase	6.E-13
Capped intron-containing pre-mRNA processing	7.E-13
Extension of telomeres	8.E-13
DNA repair	9.E-13
DNA replication pre-Initiation	1.E-12
Transport of mature mRNAs derived from intronless transcripts	2.E-12
Messenger RNA processing	3.E-12

Non-coding RNA metabolism	5.E-12
Rev-mediated nuclear export of HIV-1 RNA	5.E-12
E2F-mediated regulation of DNA replication	5.E-12
Transport of mature transcript to cytoplasm	9.E-12
E2F transcription factor network	2.E-10
G1/S-specific transcription	2.E-10
Polo-like kinase 1 (PLK1) pathway	3.E-10
ATR activation in response to replication stress	3.E-10
Unwinding of DNA	4.E-10
CDK regulation of DNA replication	8.E-10
Systemic lupus erythematosus	9.E-10
FOXM1 transcription factor network	1.E-09
Spliceosome	3.E-09
Gene expression	3.E-09
HIV life cycle	4.E-09
BARD1 signaling events	5.E-09
Transcription	2.E-08
G0 and early G1 pathway	4.E-08
Fanconi anemia pathway	4.E-08
Mismatch repair	1.E-07
Meiotic synapsis	1.E-07
Ran role in mitotic spindle regulation	3.E-07
Cyclin A/B1-associated events during G2/M transition	3.E-07
Lagging strand processive biosynthesis	3.E-07
Homologous recombination	5.E-07
BRCA1, BRCA2 and ATR roles in cancer susceptibility	5.E-07
Gap-filling DNA repair biosynthesis and ligation in global genomic nucleotide excision repair	7.E-07
MicroRNA regulation of DNA damage response	8.E-07
Kinesins	1.E-06
Leading strand biosynthesis	2.E-06
HIV infection	3.E-06

Hexose transport	5.E-06
Licensing factor removal from origins	5.E-06
Double-strand break repair	6.E-06
RNA polymerase I promoter opening	1.E-05
Antiviral mechanism by interferon-stimulated genes	1.E-05
RNA polymerase I transcription	2.E-05
HIV factor interactions with host	2.E-05
BRCA1-dependent ubiquitin ligase activity	2.E-05
Packaging of telomere ends	2.E-05
Influenza infection	2.E-05
DNA replication initiation	2.E-05
Mechanism of protein import into the nucleus	3.E-05
Sonic Hedgehog (SHH) receptor PTCH1 regulation of cell cycle	3.E-05
Oocyte meiosis	3.E-05
ATM pathway	4.E-05
RNA degradation	5.E-05
Integrated cancer pathway	6.E-05
Pyrimidine metabolism	7.E-05
RNA polymerase II transcription	8.E-05
Messenger RNA splicing: major pathway	1.E-04
Homologous DNA pairing and strand exchange	1.E-04
Global genomic nucleotide excision repair (GG-NER)	1.E-04
E2F-enabled inhibition of pre-replication complex formation	2.E-04
Cyclin B2-mediated events	2.E-04
Flap intermediate removal from the telomere lagging strand (C-strand)	2.E-04
APC/C activator regulation between G1/S and early anaphase	2.E-04
EGFR1 pathway	2.E-04
RNA polymerase I, RNA polymerase III, and mitochondrial transcription	2.E-04
Base excision repair	3.E-04
CDC6 association with the ORC-origin complex	4.E-04
Mitotic metaphase/anaphase transition	4.E-04

Progesterone-mediated oocyte maturation	4.E-04
Cyclins and cell cycle regulation	5.E-04
Integrated breast cancer pathway	5.E-04
APC/C-mediated degradation of cell cycle proteins	6.E-04
Cleavage of growing transcript in the termination region	7.E-04
Phosphorylation of Emi1	9.E-04
Rb tumor suppressor/checkpoint signaling in response to DNA damage	9.E-04
Telomerase regulation	1.E-03
APC/C- and Cdc20-mediated degradation of Nek2A	1.E-03
Amyloids	1.E-03
Nucleotide excision repair	2.E-03
Activation of Src by protein tyrosine phosphatase alpha	2.E-03
Cdc25 and Chk1 regulatory pathway in response to DNA damage	2.E-03
Cell cycle: G2/M checkpoint	2.E-03
Myc active pathway	3.E-03
Fluoropyrimidine activity	3.E-03
Disease	4.E-03
Association of licensing factors with the pre-replicative complex	4.E-03
Spliceosomal assembly	4.E-03
Nucleotide metabolism	5.E-03
Recruitment of repair and signaling proteins to double-strand breaks	6.E-03
ATM-mediated phosphorylation of repair proteins	6.E-03
Chk1/Chk2(Cds1)-mediated inactivation of cyclin B-Cdk1 complex	6.E-03
Cycling of Ran in nucleocytoplasmic transport	6.E-03
Factors involved in megakaryocyte development and platelet production	7.E-03
Messenger RNA splicing: minor pathway	8.E-03
p73 transcription factor network	8.E-03
SUMOylation by RanBP2 regulates transcriptional repression	1.E-02
G2/M DNA damage checkpoint	1.E-02
Activation of DNA fragmentation factor	1.E-02
Basic mechanisms of SUMOylation	2.E-02
Cyclin A-Cdk2-associated events at S phase entry	2.E-02

Cell cycle progression regulation by PLK3	2.E-02
T cell receptor regulation of apoptosis	3.E-02
Messenger RNA decay by 5' to 3' exoribonuclease	3.E-02
Deadenylation-dependent mRNA decay	3.E-02
FoxO family signaling	3.E-02
Purine ribonucleoside monophosphate biosynthesis	3.E-02
Inactivation of APC/C via direct inhibition of the APC/C complex	4.E-02
Inhibition of replication initiation of damaged DNA by RB1/E2F1	5.E-02
Mitochondrial protein import	5.E-02
**KEGG_2019_Human**	
**Term**	**Adjusted P-value**
Cell cycle	2.E-17
DNA replication	2.E-15
RNA transport	3.E-13
Fanconi anemia pathway	8.E-13
Systemic lupus erythematosus	3.E-10
Homologous recombination	1.E-08
Spliceosome	2.E-08
Mismatch repair	1.E-07
Alcoholism	1.E-07
Base excision repair	2.E-04
Ribosome biogenesis in eukaryotes	4.E-04
Progesterone-mediated oocyte maturation	7.E-04
Oocyte meiosis	7.E-04
Cellular senescence	8.E-04
Nucleotide excision repair	3.E-03
RNA degradation	1.E-02

**Reactome_2016**	
**Term**	**Adjusted P-value**
Cell Cycle Homo sapiens R-HSA-1640170	2.E-108
Cell Cycle, Mitotic Homo sapiens R-HSA-69278	4.E-92
M Phase Homo sapiens R-HSA-68886	1.E-45
Mitotic Prometaphase Homo sapiens R-HSA-68877	1.E-41
Resolution of Sister Chromatid Cohesion Homo sapiens R-HSA-2500257	1.E-38
Chromosome Maintenance Homo sapiens R-HSA-73886	5.E-33
DNA Repair Homo sapiens R-HSA-73894	4.E-30
Mitotic Metaphase and Anaphase Homo sapiens R-HSA-2555396	9.E-30
Mitotic Anaphase Homo sapiens R-HSA-68882	3.E-29
Separation of Sister Chromatids Homo sapiens R-HSA-2467813	6.E-29
Cell Cycle Checkpoints Homo sapiens R-HSA-69620	1.E-26
DNA Double-Strand Break Repair Homo sapiens R-HSA-5693532	2.E-26
RHO GTPases Activate Formins Homo sapiens R-HSA-5663220	2.E-26
Homology Directed Repair Homo sapiens R-HSA-5693538	2.E-25
Gene Expression Homo sapiens R-HSA-74160	4.E-25
G2/M Checkpoints Homo sapiens R-HSA-69481	8.E-24
S Phase Homo sapiens R-HSA-69242	4.E-23
HDR through Homologous Recombination (HR) or Single Strand Annealing (SSA) Homo sapiens R-HSA-5693567	3.E-22
RHO GTPase Effectors Homo sapiens R-HSA-195258	3.E-22
DNA strand elongation Homo sapiens R-HSA-69190	7.E-21
Activation of ATR in response to replication stress Homo sapiens R-HSA-176187	9.E-21
Activation of the pre-replicative complex Homo sapiens R-HSA-68962	9.E-21
Deposition of new CENPA-containing nucleosomes at the centromere Homo sapiens R-HSA-606279	3.E-20
Nucleosome assembly Homo sapiens R-HSA-774815	3.E-20
Mitotic G1-G1/S phases Homo sapiens R-HSA-453279	5.E-20

Homologous DNA Pairing and Strand Exchange Homo sapiens R-HSA-5693579	1.E-19
DNA Replication Homo sapiens R-HSA-69306	2.E-19
Processing of DNA double-strand break ends Homo sapiens R-HSA-5693607	1.E-18
Presynaptic phase of homologous DNA pairing and strand exchange Homo sapiens R-HSA-5693616	2.E-18
HDR through Homologous Recombination (HRR) Homo sapiens R-HSA-5685942	2.E-18
Mitotic Prophase Homo sapiens R-HSA-68875	3.E-18
G1/S Transition Homo sapiens R-HSA-69206	4.E-18
Processing of Capped Intron-Containing Pre-mRNA Homo sapiens R-HSA-72203	7.E-18
Telomere Maintenance Homo sapiens R-HSA-157579	7.E-18
Signaling by Rho GTPases Homo sapiens R-HSA-194315	1.E-17
SUMOylation of DNA replication proteins Homo sapiens R-HSA-4615885	1.E-17
Mitotic G2-G2/M phases Homo sapiens R-HSA-453274	6.E-17
SUMOylation Homo sapiens R-HSA-2990846	6.E-17
Meiosis Homo sapiens R-HSA-1500620	7.E-17
G2/M DNA damage checkpoint Homo sapiens R-HSA-69473	1.E-16
G2/M Transition Homo sapiens R-HSA-69275	1.E-16
Interactions of Rev with host cellular proteins Homo sapiens R-HSA-177243	2.E-16
Transcriptional regulation by small RNAs Homo sapiens R-HSA-5578749	3.E-16
Meiotic recombination Homo sapiens R-HSA-912446	2.E-16
Synthesis of DNA Homo sapiens R-HSA-69239	4.E-16
Transport of Mature Transcript to Cytoplasm Homo sapiens R-HSA-72202	7.E-16
SUMO E3 ligases SUMOylate target proteins Homo sapiens R-HSA-3108232	2.E-15
Rev-mediated nuclear export of HIV RNA Homo sapiens R-HSA-165054	8.E-15

Transport of Mature mRNA derived from an Intron-Containing Transcript Homo sapiens R-HSA-159236	2.E-14
AURKA Activation by TPX2 Homo sapiens R-HSA-8854518	2.E-14
HDR through Single Strand Annealing (SSA) Homo sapiens R-HSA-5685938	2.E-14
Cellular responses to stress Homo sapiens R-HSA-2262752	2.E-14
Nuclear import of Rev protein Homo sapiens R-HSA-180746	5.E-14
Regulation of TP53 Activity through Phosphorylation Homo sapiens R-HSA-6804756	1.E-13
Extension of Telomeres Homo sapiens R-HSA-180786	3.E-13
Export of Viral Ribonucleoproteins from Nucleus Homo sapiens R-HSA-168274	4.E-13
Gene Silencing by RNA Homo sapiens R-HSA-211000	4.E-13
Centrosome maturation Homo sapiens R-HSA-380287	5.E-13
Recruitment of mitotic centrosome proteins and complexes Homo sapiens R-HSA-380270	5.E-13
Loss of Nlp from mitotic centrosomes Homo sapiens R-HSA-380259	2.E-12
Loss of proteins required for interphase microtubule organization?from the centrosome Homo sapiens R-HSA-380284	1.E-12
DNA Replication Pre-Initiation Homo sapiens R-HSA-69002	2.E-12
M/G1 Transition Homo sapiens R-HSA-68874	2.E-12
Metabolism of non-coding RNA Homo sapiens R-HSA-194441	2.E-12
snRNP Assembly Homo sapiens R-HSA-191859	2.E-12
Regulation of PLK1 Activity at G2/M Transition Homo sapiens R-HSA-2565942	2.E-12
E2F mediated regulation of DNA replication Homo sapiens R-HSA-113510	2.E-12
NEP/NS2 Interacts with the Cellular Export Machinery Homo sapiens R-HSA-168333	2.E-12
Telomere C-strand (Lagging Strand) Synthesis Homo sapiens R-HSA-174417	3.E-12
Nuclear Envelope Breakdown Homo sapiens R-HSA-2980766	3.E-12
DNA Damage/Telomere Stress Induced Senescence Homo sapiens R-HSA-2559586	4.E-12

Nuclear Pore Complex (NPC) Disassembly Homo sapiens R-HSA-3301854	4.E-12
Condensation of Prophase Chromosomes Homo sapiens R-HSA-2299718	5.E-12
Transport of Mature mRNAs Derived from Intronless Transcripts Homo sapiens R-HSA-159234	7.E-12
Interactions of Vpr with host cellular proteins Homo sapiens R-HSA-176033	9.E-12
Vpr-mediated nuclear import of PICs Homo sapiens R-HSA-180910	1.E-11
Resolution of D-loop Structures through Holliday Junction Intermediates Homo sapiens R-HSA-5693568	1.E-11
Resolution of D-loop Structures through Synthesis-Dependent Strand Annealing (SDSA) Homo sapiens R-HSA-5693554	1.E-11
Anchoring of the basal body to the plasma membrane Homo sapiens R-HSA-5620912	1.E-11
Unwinding of DNA Homo sapiens R-HSA-176974	2.E-11
SUMOylation of DNA damage response and repair proteins Homo sapiens R-HSA-3108214	2.E-11
Resolution of D-Loop Structures Homo sapiens R-HSA-5693537	2.E-11
Transport of Ribonucleoproteins into the Host Nucleus Homo sapiens R-HSA-168271	3.E-11
Transport of Mature mRNA Derived from an Intronless Transcript Homo sapiens R-HSA-159231	4.E-11
Transport of the SLBP Dependant Mature mRNA Homo sapiens R-HSA-159230	5.E-11
G1/S-Specific Transcription Homo sapiens R-HSA-69205	8.E-11
Lagging Strand Synthesis Homo sapiens R-HSA-69186	1.E-10
NS1 Mediated Effects on Host Pathways Homo sapiens R-HSA-168276	1.E-10
Epigenetic regulation of gene expression Homo sapiens R-HSA-212165	2.E-10
PRC2 methylates histones and DNA Homo sapiens R-HSA-212300	3.E-10
Transport of the SLBP independent Mature mRNA Homo sapiens R-HSA-159227	3.E-10
Regulation of Glucokinase by Glucokinase Regulatory Protein Homo sapiens R-HSA-170822	4.E-10

Transcriptional Regulation by TP53 Homo sapiens R-HSA-3700989	5.E-10
Host Interactions with Influenza Factors Homo sapiens R-HSA-168253	5.E-10
tRNA processing in the nucleus Homo sapiens R-HSA-6784531	5.E-10
SUMOylation of RNA binding proteins Homo sapiens R-HSA-4570464	5.E-10
Regulation of HSF1-mediated heat shock response Homo sapiens R-HSA-3371453	6.E-10
Cellular response to heat stress Homo sapiens R-HSA-3371556	1.E-09
Cellular Senescence Homo sapiens R-HSA-2559583	1.E-09
Regulation of TP53 Activity Homo sapiens R-HSA-5633007	2.E-09
Viral Messenger RNA Synthesis Homo sapiens R-HSA-168325	7.E-09
HIV Life Cycle Homo sapiens R-HSA-162587	8.E-09
SIRT1 negatively regulates rRNA Expression Homo sapiens R-HSA-427359	1.E-08
Base Excision Repair Homo sapiens R-HSA-73884	1.E-08
Resolution of Abasic Sites (AP sites) Homo sapiens R-HSA-73933	1.E-08
Fanconi Anemia Pathway Homo sapiens R-HSA-6783310	1.E-08
G0 and Early G1 Homo sapiens R-HSA-1538133	2.E-08
mRNA Splicing Homo sapiens R-HSA-72172	2.E-08
Polo-like kinase mediated events Homo sapiens R-HSA-156711	2.E-08
Resolution of AP sites via the multiple-nucleotide patch replacement pathway Homo sapiens R-HSA-110373	2.E-08
Late Phase of HIV Life Cycle Homo sapiens R-HSA-162599	2.E-08
mRNA Splicing - Major Pathway Homo sapiens R-HSA-72163	2.E-08
Removal of the Flap Intermediate Homo sapiens R-HSA-69166	3.E-08
Positive epigenetic regulation of rRNA expression Homo sapiens R-HSA-5250913	4.E-08
ERCC6 (CSB) and EHMT2 (G9a) positively regulate rRNA expression Homo sapiens R-HSA-427389	8.E-08
Processive synthesis on the lagging strand Homo sapiens R-HSA-69183	1.E-07
Meiotic synapsis Homo sapiens R-HSA-1221632	1.E-07
tRNA processing Homo sapiens R-HSA-72306	1.E-07
DNA methylation Homo sapiens R-HSA-5334118	1.E-07

Cyclin A/B1 associated events during G2/M transition Homo sapiens R-HSA-69273	2.E-07
PCNA-Dependent Long Patch Base Excision Repair Homo sapiens R-HSA-5651801	3.E-07
RNA Polymerase II Transcription Homo sapiens R-HSA-73857	3.E-07
Glucose transport Homo sapiens R-HSA-70153	3.E-07
Negative epigenetic regulation of rRNA expression Homo sapiens R-HSA-5250941	5.E-07
Assembly of the primary cilium Homo sapiens R-HSA-5617833	5.E-07
RNA Polymerase I Chain Elongation Homo sapiens R-HSA-73777	6.E-07
Polymerase switching on the C-strand of the telomere Homo sapiens R-HSA-174411	8.E-07
Polymerase switching Homo sapiens R-HSA-69091	8.E-07
Leading Strand Synthesis Homo sapiens R-HSA-69109	7.E-07
Mismatch repair (MMR) directed by MSH2:MSH6 (MutSalpha) Homo sapiens R-HSA-5358565	7.E-07
B-WICH complex positively regulates rRNA expression Homo sapiens R-HSA-5250924	8.E-07
RNA Polymerase I Transcription Homo sapiens R-HSA-73864	1.E-06
Recognition of DNA damage by PCNA-containing replication complex Homo sapiens R-HSA-110314	1.E-06
Regulation of DNA replication Homo sapiens R-HSA-69304	1.E-06
Cleavage of Growing Transcript in the Termination Region Homo sapiens R-HSA-109688	2.E-06
RNA Polymerase II Transcription Termination Homo sapiens R-HSA-73856	2.E-06
Post-Elongation Processing of the Transcript Homo sapiens R-HSA-76044	2.E-06
RNA Polymerase I Promoter Clearance Homo sapiens R-HSA-73854	2.E-06
HIV Infection Homo sapiens R-HSA-162906	2.E-06
Mismatch Repair Homo sapiens R-HSA-5358508	2.E-06
ISG15 antiviral mechanism Homo sapiens R-HSA-1169408	3.E-06
Antiviral mechanism by IFN-stimulated genes Homo sapiens R-HSA-1169410	3.E-06

Influenza Infection Homo sapiens R-HSA-168254	3.E-06
Host Interactions of HIV factors Homo sapiens R-HSA-162909	3.E-06
RNA Polymerase I Promoter Opening Homo sapiens R-HSA-73728	3.E-06
Activation of anterior HOX genes in hindbrain development during early embryogenesis Homo sapiens R-HSA-5617472	3.E-06
Activation of HOX genes during differentiation Homo sapiens R-HSA-5619507	3.E-06
Influenza Life Cycle Homo sapiens R-HSA-168255	3.E-06
NoRC negatively regulates rRNA expression Homo sapiens R-HSA-427413	3.E-06
Hexose transport Homo sapiens R-HSA-189200	3.E-06
Establishment of Sister Chromatid Cohesion Homo sapiens R-HSA-2468052	4.E-06
HDR through MMEJ (alt-NHEJ) Homo sapiens R-HSA-5685939	4.E-06
Gap-filling DNA repair synthesis and ligation in GG-NER Homo sapiens R-HSA-5696397	4.E-06
Packaging Of Telomere Ends Homo sapiens R-HSA-171306	4.E-06
Mitotic Telophase/Cytokinesis Homo sapiens R-HSA-68884	4.E-06
RMTs methylate histone arginines Homo sapiens R-HSA-3214858	6.E-06
HDACs deacetylate histones Homo sapiens R-HSA-3214815	6.E-06
Senescence-Associated Secretory Phenotype (SASP) Homo sapiens R-HSA-2559582	9.E-06
DNA replication initiation Homo sapiens R-HSA-68952	9.E-06
Telomere C-strand synthesis initiation Homo sapiens R-HSA-174430	9.E-06
Removal of licensing factors from origins Homo sapiens R-HSA-69300	1.E-05
Mismatch repair (MMR) directed by MSH2:MSH3 (MutSbeta) Homo sapiens R-HSA-5358606	1.E-05
Condensation of Prometaphase Chromosomes Homo sapiens R-HSA-2514853	1.E-05
Activated PKN1 stimulates transcription of AR (androgen receptor) regulated genes KLK2 and KLK3 Homo sapiens R-HSA-5625886	2.E-05
Infectious disease Homo sapiens R-HSA-5663205	2.E-05
Termination of translesion DNA synthesis Homo sapiens R-HSA-5656169	2.E-05

RNA Polymerase I, RNA Polymerase III, and Mitochondrial Transcription Homo sapiens R-HSA-504046	2.E-05
DNA Double Strand Break Response Homo sapiens R-HSA-5693606	2.E-05
Kinesins Homo sapiens R-HSA-983189	2.E-05
Global Genome Nucleotide Excision Repair (GG-NER) Homo sapiens R-HSA-5696399	3.E-05
mRNA 3'-end processing Homo sapiens R-HSA-72187	4.E-05
Post-Elongation Processing of Intron-Containing pre-mRNA Homo sapiens R-HSA-112296	4.E-05
DNA Damage Bypass Homo sapiens R-HSA-73893	4.E-05
Assembly of the pre-replicative complex Homo sapiens R-HSA-68867	4.E-05
rRNA processing Homo sapiens R-HSA-72312	5.E-05
Oxidative Stress Induced Senescence Homo sapiens R-HSA-2559580	5.E-05
Influenza Viral RNA Transcription and Replication Homo sapiens R-HSA-168273	5.E-05
rRNA modification in the nucleus Homo sapiens R-HSA-6790901	6.E-05
Formation of the beta-catenin:TCF transactivating complex Homo sapiens R-HSA-201722	6.E-05
Recruitment and ATM-mediated phosphorylation of repair and signaling proteins at DNA double strand breaks Homo sapiens R-HSA-5693565	6.E-05
TP53 Regulates Transcription of Cell Cycle Genes Homo sapiens R-HSA-6791312	7.E-05
Removal of the Flap Intermediate from the C-strand Homo sapiens R-HSA-174437	7.E-05
E2F-enabled inhibition of pre-replication complex formation Homo sapiens R-HSA-113507	7.E-05
Cyclin B2 mediated events Homo sapiens R-HSA-157881	8.E-05
Switching of origins to a post-replicative state Homo sapiens R-HSA-69052	8.E-05
Orc1 removal from chromatin Homo sapiens R-HSA-68949	8.E-05
Transcription-Coupled Nucleotide Excision Repair (TC-NER) Homo sapiens R-HSA-6781827	9.E-05
Dual Incision in GG-NER Homo sapiens R-HSA-5696400	1.E-04

Major pathway of rRNA processing in the nucleolus Homo sapiens R-HSA-6791226	1.E-04
Nucleotide Excision Repair Homo sapiens R-HSA-5696398	1.E-04
Organelle biogenesis and maintenance Homo sapiens R-HSA-1852241	1.E-04
APC/C-mediated degradation of cell cycle proteins Homo sapiens R-HSA-174143	2.E-04
Regulation of mitotic cell cycle Homo sapiens R-HSA-453276	2.E-04
Processive synthesis on the C-strand of the telomere Homo sapiens R-HSA-174414	2.E-04
CDC6 association with the ORC:origin complex Homo sapiens R-HSA-68689	2.E-04
Chromatin organization Homo sapiens R-HSA-4839726	2.E-04
Chromatin modifying enzymes Homo sapiens R-HSA-3247509	2.E-04
Nonhomologous End-Joining (NHEJ) Homo sapiens R-HSA-5693571	2.E-04
Translesion synthesis by Y family DNA polymerases bypasses lesions on DNA template Homo sapiens R-HSA-110313	2.E-04
HATs acetylate histones Homo sapiens R-HSA-3214847	3.E-04
RHO GTPases activate PKNs Homo sapiens R-HSA-5625740	4.E-04
Phosphorylation of Emi1 Homo sapiens R-HSA-176417	4.E-04
Translesion synthesis by REV1 Homo sapiens R-HSA-110312	4.E-04
Cohesin Loading onto Chromatin Homo sapiens R-HSA-2470946	5.E-04
TP53 Regulates Transcription of DNA Repair Genes Homo sapiens R-HSA-6796648	5.E-04
Gap-filling DNA repair synthesis and ligation in TC-NER Homo sapiens R-HSA-6782210	6.E-04
Translesion synthesis by POLK Homo sapiens R-HSA-5655862	7.E-04
Translesion synthesis by POLI Homo sapiens R-HSA-5656121	7.E-04
Translesion Synthesis by POLH Homo sapiens R-HSA-110320	2.E-03
Metabolism of nucleotides Homo sapiens R-HSA-15869	2.E-03
Association of licensing factors with the pre-replicative complex Homo sapiens R-HSA-69298	2.E-03
Generic Transcription Pathway Homo sapiens R-HSA-212436	2.E-03
HSF1 activation Homo sapiens R-HSA-3371511	2.E-03

Activation of APC/C and APC/C:Cdc20 mediated degradation of mitotic proteins Homo sapiens R-HSA-176814	2.E-03
Dual incision in TC-NER Homo sapiens R-HSA-6782135	2.E-03
PKMTs methylate histone lysines Homo sapiens R-HSA-3214841	2.E-03
mRNA Splicing - Minor Pathway Homo sapiens R-HSA-72165	3.E-03
G2/M DNA replication checkpoint Homo sapiens R-HSA-69478	3.E-03
Regulation of APC/C activators between G1/S and early anaphase Homo sapiens R-HSA-176408	4.E-03
Metabolism of proteins Homo sapiens R-HSA-392499	4.E-03
Factors involved in megakaryocyte development and platelet production Homo sapiens R-HSA-983231	4.E-03
Formation of Senescence-Associated Heterochromatin Foci (SAHF) Homo sapiens R-HSA-2559584	5.E-03
APC/C:Cdc20 mediated degradation of mitotic proteins Homo sapiens R-HSA-176409	5.E-03
Amyloid fiber formation Homo sapiens R-HSA-977225	6.E-03
Processing and activation of SUMO Homo sapiens R-HSA-3215018	6.E-03
TP53 Regulates Transcription of Genes Involved in G1 Cell Cycle Arrest Homo sapiens R-HSA-6804116	6.E-03
Activation of DNA fragmentation factor Homo sapiens R-HSA-211227	6.E-03
Apoptosis induced DNA fragmentation Homo sapiens R-HSA-140342	6.E-03
TP53 Regulates Transcription of Genes Involved in G2 Cell Cycle Arrest Homo sapiens R-HSA-6804114	7.E-03
SUMO is transferred from E1 to E2 (UBE2I, UBC9) Homo sapiens R-HSA-3065678	8.E-03
Phosphorylation of the APC/C Homo sapiens R-HSA-176412	1.E-02
APC-Cdc20 mediated degradation of Nek2A Homo sapiens R-HSA-179409	1.E-02
mRNA decay by 5' to 3' exoribonuclease Homo sapiens R-HSA-430039	1.E-02
COPI-dependent Golgi-to-ER retrograde traffic Homo sapiens R-HSA-6811434	1.E-02
Inhibition of the proteolytic activity of APC/C required for the onset of anaphase by mitotic spindle checkpoint components Homo sapiens R-HSA-141405	1.E-02

Inactivation of APC/C via direct inhibition of the APC/C complex Homo sapiens R-HSA-141430	1.E-02
Cyclin E associated events during G1/S transition Homo sapiens R-HSA-69202	2.E-02
Activation of NIMA Kinases NEK9, NEK6, NEK7 Homo sapiens R-HSA-2980767	2.E-02
Cyclin A:Cdk2-associated events at S phase entry Homo sapiens R-HSA-69656	2.E-02
Deadenylation-dependent mRNA decay Homo sapiens R-HSA-429914	2.E-02
Mitotic Spindle Checkpoint Homo sapiens R-HSA-69618	2.E-02
Association of TriC/CCT with target proteins during biosynthesis Homo sapiens R-HSA-390471	2.E-02
Chk1/Chk2(Cds1) mediated inactivation of Cyclin B:Cdk1 complex Homo sapiens R-HSA-75035	3.E-02
Inhibition of replication initiation of damaged DNA by RB1/E2F1 Homo sapiens R-HSA-113501	3.E-02
Purine ribonucleoside monophosphate biosynthesis Homo sapiens R-HSA-73817	3.E-02
APC:Cdc20 mediated degradation of cell cycle proteins prior to satisfation of the cell cycle checkpoint Homo sapiens R-HSA-179419	3.E-02
APC/C:Cdc20 mediated degradation of Cyclin B Homo sapiens R-HSA-174048	3.E-02
Mitochondrial protein import Homo sapiens R-HSA-1268020	4.E-02
Post-Elongation Processing of Intronless pre-mRNA Homo sapiens R-HSA-112297	4.E-02
Processing of Capped Intronless Pre-mRNA Homo sapiens R-HSA-75067	4.E-02
Pyrimidine metabolism Homo sapiens R-HSA-73848	4.E-02
Cytosolic tRNA aminoacylation Homo sapiens R-HSA-379716	4.E-02

Supplementary table 4. Gene ontology enrichment analysis of 1561 genes upregulated by ALKBH4 knockdown

**BioPlanet_2019**	
**Term**	**Adjusted P-value**

p53 signaling pathway	2.E-07
TAp63 pathway	1.E-03
ECM-receptor interaction	1.E-03
Beta-1 integrin cell surface interactions	2.E-03
Integrin cell surface interactions	1.E-02
p53 activity regulation	1.E-02
Platelet endothelial cell adhesion molecule 1 (PECAM1) interactions	2.E-02
Beta-3 integrin cell surface interactions	4.E-02
BDNF signaling pathway	4.E-02
**KEGG_2019_Human**	
**Term**	**Adjusted P-value**
p53 signaling pathway	7.E-05
AGE-RAGE signaling pathway in diabetic complications	4.E-04
Small cell lung cancer	4.E-04
ECM-receptor interaction	6.E-04
**Reactome_2016**	
**Term**	**Adjusted P-value**
Extracellular matrix organization Homo sapiens R-HSA-1474244	1.E-04
Non-integrin membrane-ECM interactions Homo sapiens R-HSA-3000171	1.E-04
Laminin interactions Homo sapiens R-HSA-3000157	9.E-03
Anchoring fibril formation Homo sapiens R-HSA-2214320	1.E-02
Syndecan interactions Homo sapiens R-HSA-3000170	1.E-02
PECAM1 interactions Homo sapiens R-HSA-210990	2.E-02
Integrin cell surface interactions Homo sapiens R-HSA-216083	2.E-02

Assembly of collagen fibrils and other multimeric structures Homo sapiens R-HSA-2022090	2.E-02
ECM proteoglycans Homo sapiens R-HSA-3000178	2.E-02
TP53 Regulates Transcription of Cell Death Genes Homo sapiens R-HSA-5633008	3.E-02

Supplementary table 5. Enrichment analysis using ChEA database

Gene Symbol	Fold-Change (ALKBH4 KD vs. Control)
ACD	-2.50193
ACTL6A	-1.77064
ANP32B	-1.79466
ATAD2	-9.53211
AURKB	-10.754
BRCA2	-9.36104
BUB3	-1.63836
C16orf59	-1.6253
C1orf131	-1.64172
C1orf174	-1.74185
C3orf14	-2.48678
CACYBP	-1.54571
CALM3	-1.73824
CASC5	-8.21945
CASP2	-1.76126
CBX5	-1.85256
CCNE1	-2.91155
CCT4	-1.9579
CDC23	-1.50366
CDC25A	-5.20816
CDC5L	-1.53657
CDC6	-7.17895

CDC7	-1.82304
CDCA5	-3.63728
CDCA7	-2.41964
CDCA7L	-2.53119
CDKN1B	-1.57594
CDT1	-2.00057
CENPA	-3.24485
CENPH	-7.33772
CENPM	-3.117
CHEK1	-2.28322
CHRAC1	-1.61056
CLSPN	-4.99926
COPS3	-1.53682
COX17	-1.6222
CYCS	-1.62691
DCK	-1.66166
DDX46	-1.78366
DEK	-3.01202
DHX40	-1.59591
DHX9	-1.56527
DIAPH1	-1.50993
DNAJC9	-2.3883
DNMT1	-2.92157
DONSON	-2.11479
DTYMK	-2.4384
DUT	-1.72181
E2F2	-1.9599
E2F8	-6.83216
EED	-1.69207
EIF5A2	-1.54427
EMP2	-3.06387
ERH	-1.69808

EXO1	-9.92716
EXOSC9	-2.43455
EZH2	-3.39208
FAM111B	-13.9955
FANCA	-4.26537
FANCC	-1.56328
FANCD2	-7.40383
FANCG	-2.51414
FBL	-1.89431
FBXO5	-4.33098
FKBP5	-1.71484
FUS	-1.62278
GARS	-1.52488
GDI2	-1.56455
GINS1	-3.17338
GINS2	-13.7191
GINS3	-3.60597
GMNN	-3.2306
GMPS	-1.83129
GPD2	-2.61095
H2AFV	-2.10284
H2AFZ	-2.7353
HELLS	-7.56267
HIST1H1D	-3.14904
HIST1H1E	-2.17113
HIST1H2AB	-6.81241
HIST1H2AC	-1.94685
HIST1H2AK	-5.08953
HIST1H2AM	-6.31514
HIST1H2BL	-5.05341
HIST1H3F	-1.84152
HIST1H4A	-3.75718

HIST1H4B	-4.39541
HIST1H4C	-2.78565
HMGA1	-1.61283
HMGB2	-3.42835
ILF3	-1.50856
ING3	-1.60667
INTS7	-1.70965
IPO11	-1.90756
KCTD1	-1.83582
KEAP1	-1.51956
KIAA0101	-8.3741
KIAA1429	-1.55439
KPNA2	-1.7797
LHX2	-1.54421
LIN9	-2.38411
LMNB1	-5.3732
LMNB2	-1.94684
LRRCC1	-1.85013
LSM6	-1.98489
MATR3	-1.5307
MBD4	-1.6354
MCM2	-4.1169
MCM3	-3.8761
MCM5	-4.64867
MCM6	-5.87355
MCM7	-5.4882
MND1	-10.6562
MNS1	-6.10306
MSH2	-2.95554
MSH6	-2.9654
MTF2	-1.68157
MTHFD1	-1.51007

MYC	-1.54758
NASP	-3.12178
NBN	-1.52197
NCAPD3	-3.58621
NCAPG2	-5.74406
NCAPH2	-1.50056
NCL	-1.56473
NOLC1	-1.79403
NUCB1	-3.31294
NUCKS1	-2.8088
NUP153	-1.76494
NUP155	-2.32816
NUP160	-1.89072
OAS3	-1.98557
OIP5	-6.90794
OSBPL10	-1.50652
PAQR4	-2.46477
PBK	-14.922
PBX3	-1.60164
PCNA	-1.51214
PER2	-1.52157
PHB	-1.94756
PHF5A	-2.02937
PKMYT1	-2.63339
PLAA	-1.50617
PNN	-1.57406
POLA2	-4.14265
POLD1	-1.8732
POLE	-3.46654
POLE2	-5.93548
POLR1E	-2.37765
PPARG	-1.5889

PPIG	-1.69988
PPM1G	-2.84341
PSMC3IP	-4.01797
RBL1	-5.32118
RDX	-1.55924
RFC3	-8.31795
RFWD3	-3.28861
RIPK1	-1.53898
RNASEH2A	-2.55655
RPA2	-2.4916
RPA3	-1.92365
RPL14	-1.88147
RPP30	-1.86945
RPS6KA5	-1.61996
RRM1	-3.54669
RRM2	-9.02179
SART3	-1.65059
SCLT1	-1.55121
SEH1L	-1.73893
SHMT2	-1.5965
SIX4	-1.54332
SLC16A6	-3.86598
SLC20A1	-1.60206
SLC25A19	-1.70309
SLC31A1	-3.21496
SMC1A	-1.61706
SMC3	-3.30398
SNRPB	-1.57096
SNRPD1	-2.20164
SSRP1	-1.66567
STIL	-6.18652
STT3B	-1.74834

SUGT1	-1.78735
SUV39H1	-4.50538
SUV39H2	-3.0486
SYNE2	-1.93363
TAF6	-2.37763
TATDN2	-1.53959
TBC1D15	-1.59502
TCP1	-1.87973
THAP10	-2.87321
THOC2	-1.57013
TIMELESS	-2.69009
TIPIN	-2.89066
TMEM109	-3.21988
TMEM116	-1.78465
TMEM126B	-1.5057
TMEM97	-1.52618
TMPO	-3.59943
TNPO3	-1.68624
TOP3A	-1.51141
TOPBP1	-3.34041
TPP2	-1.73727
TTLL4	-1.53482
TTLL7	-1.56992
UNG	-3.75083
USP37	-1.64209
VPS29	-1.748
VRK1	-4.10896
XRCC2	-6.0895
YEATS4	-2.33593
ZCCHC8	-1.57617
ZMYND19	-1.78624
ZNF367	-2.42225

ZNF718	-1.83011

Supplementary Figures

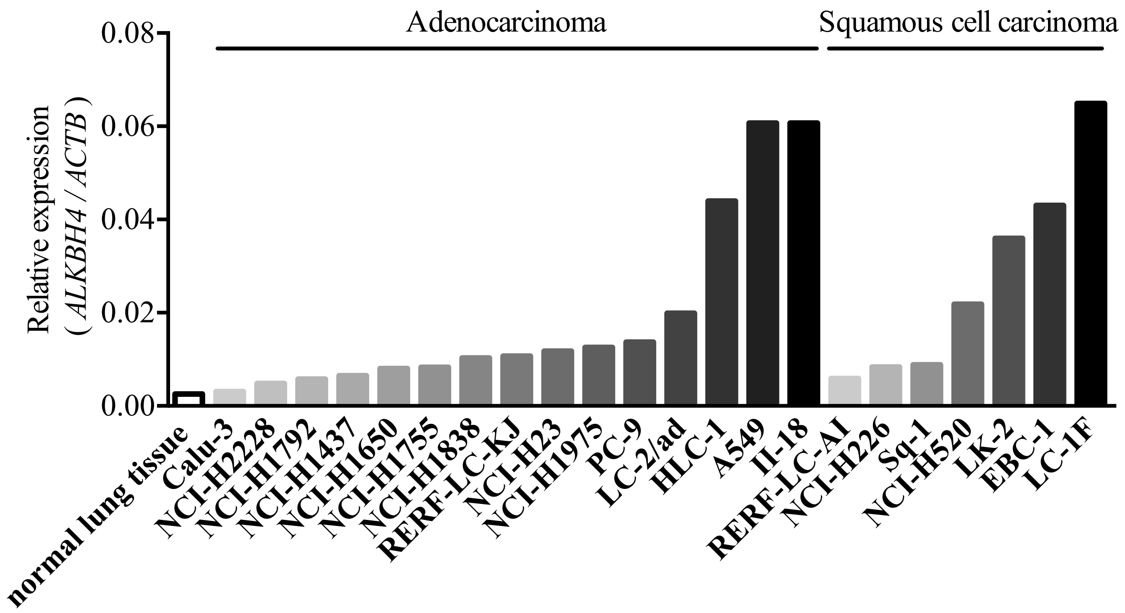

Supplementary Figure 1. Expression of *ALKBH4* in NSCLC cell lines.

Twenty-two NSCLC cell lines (15 adenocarcinoma cell lines and 7 squamous cell carcinoma cell lines) and an RNA mixture of 3 normal lung tissues which were randomly selected from postoperative tissues of NSCLC patients were subjected to qPCR analysis of *ALKBH4* and *ACTB*. Values are represented as mean ± S.D. in duplicates.

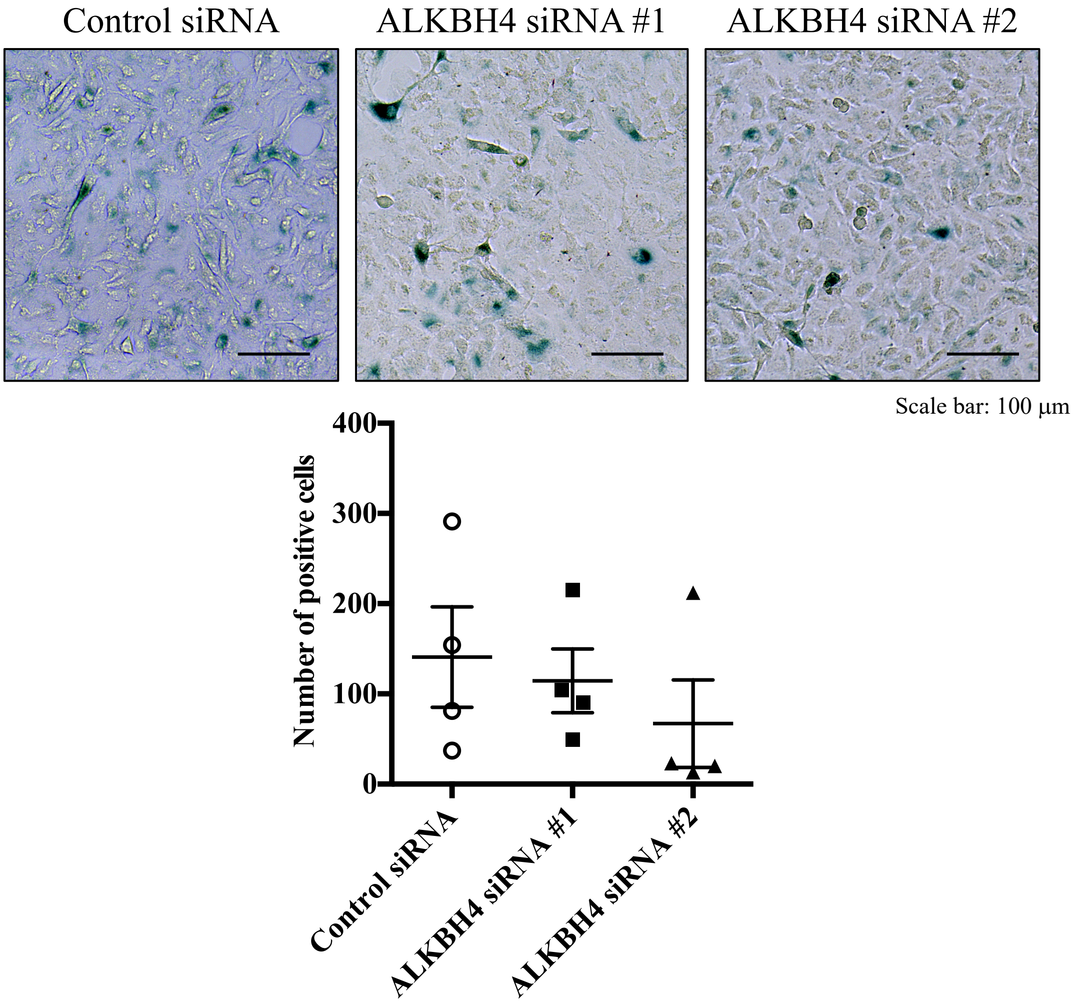

Supplementary Figure 2. G_0-marker analysis.

A549 cells transfected with ALKBH4 siRNAs were cultured for 72 h, and G_0-marker assays were conducted using senescence-associated β-galactosidase. Representative results of three independent experiments are shown in the upper panel. The numbers of β-galactosidase-positive cells are represented as the mean ± S.D. of three independent experiments.

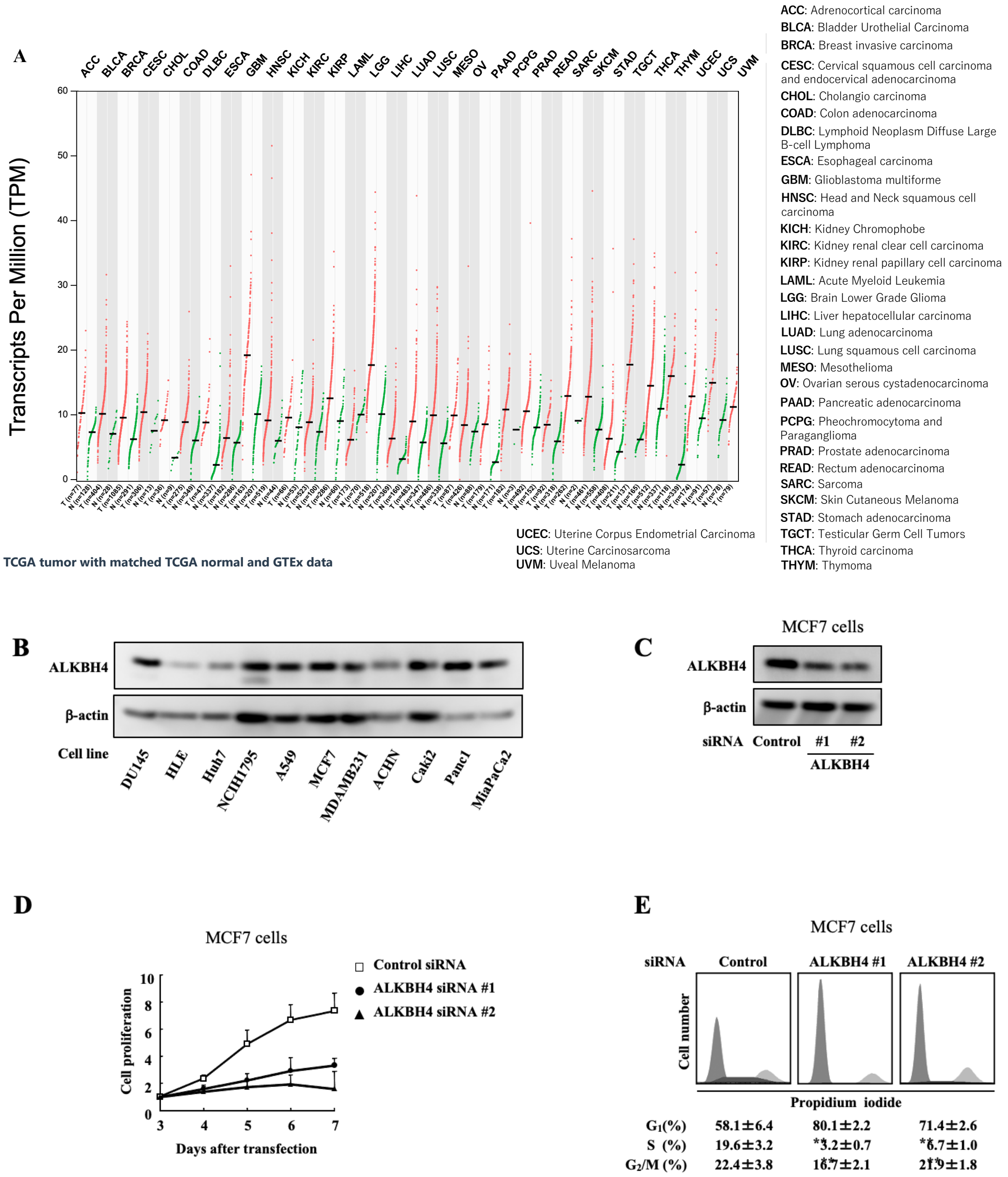

Supplementary Figure 3. ALKBH4 knockdown reduced cell proliferation in MCF-7 cells.

A, ALKBH4 gene expression profile across various tumour tissues and the paired normal tissues were analysed using GEPIA web server. Normal tissue samples and

tumour tissue samples are indicated by green and red dots, respectively. **B**, Expression of ALKBH4 and β-actin were examined in prostate (DU145), liver (HLE and Huh7), lungs (NCIH1795 and A549), breast (MCF7 and MDAMB231), renal (ACHN and Caki2) and pancreatic (Panc1 and MiaPaCa2) cancer cell lines. Uncropped Western blot data are shown in Supplementary Fig. 9. **C**, MCF-7 cells transfected with ALKBH4 siRNAs or control siRNA were incubated for 48 h and were subjected to Western blot analysis with anti-ALKBH4 and anti-β-actin antibodies. Uncropped Western blot data are shown in Supplementary Fig. 9. Representative results of three independent experiments are shown. **D**, MCF-7 cells transfected with ALKBH4 siRNA or control were reseeded on a 96-well plate and cell proliferation was examined using WST-1 assay. The data are shown as mean ± S.D. of three independent experiments. **E**, MCF-7 cells were transfected with ALKBH4 siRNAs or control siRNA for 48 h. The cells were stained with (a PI solution and the cell cycle was analysed using a flow cytometer. Percentages of each cell cycle population are represented as means ± S.D. of three independent experiments. *$p < 0.05$ vs. control siRNA.

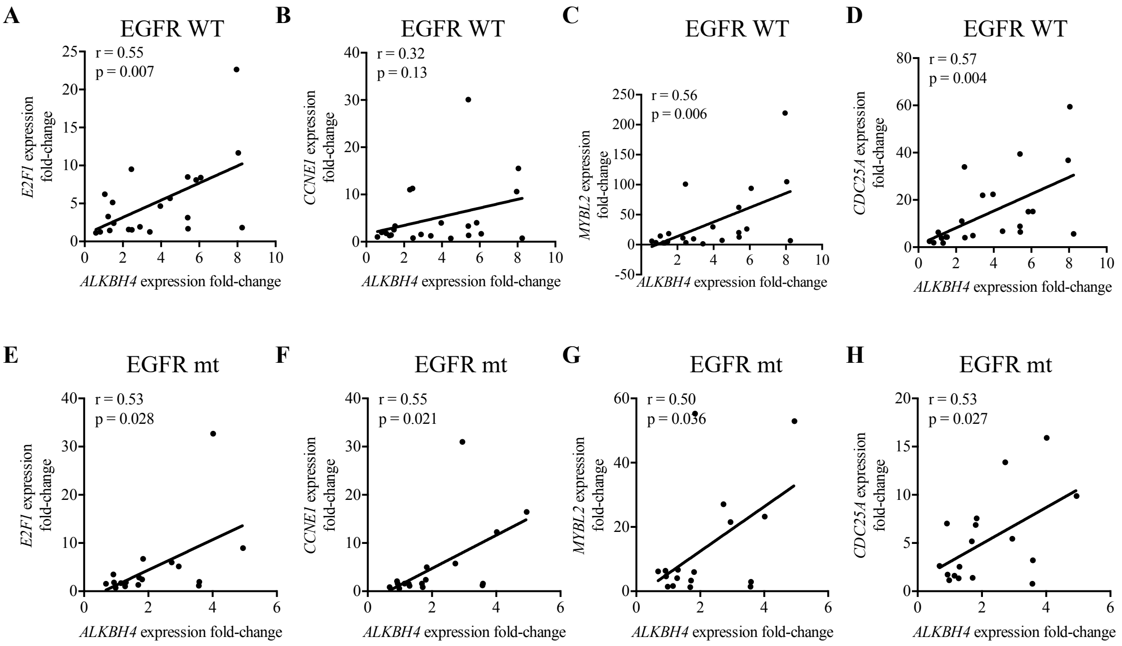

Supplementary Figure 4. The expression of *ALKBH4* and *E2F1* or E2F1-target genes has a positive correlation in NSCLC, regardless of *EGFR* gene mutation.

Correlation analysis was performed between the relative expression of *ALKBH4* and *E2F1* (**A** and **E**), *CCNE1* (**B** and **F**), *MYBL2* (**C** and **G**), and *CDC25A* (**D** and **H**) in NSCLC specimens with wild-type (WT) and mutant (mt) EGFR. Pearson correlation analysis was conducted.

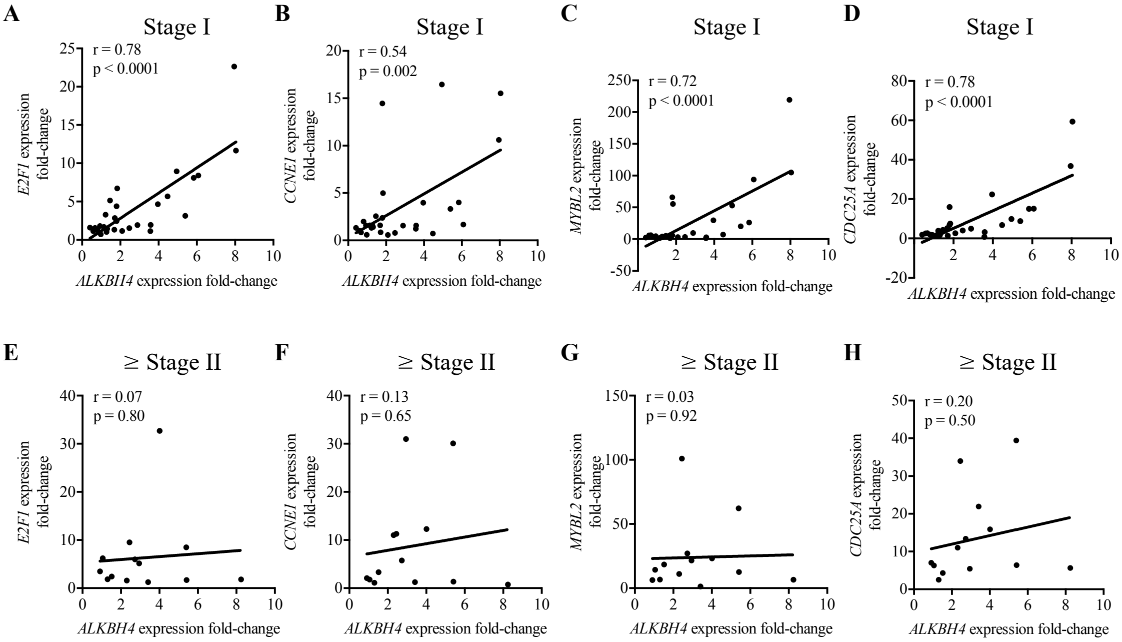

Supplementary Figure 5. The expression of *ALKBH4* and *E2F1* or E2F1-target genes only has a positive correlation in the early stage of NSCLC.

Correlation analysis was performed between the relative expression of *ALKBH4* and *E2F1* (**A** and **E**), *CCNE1* expression (**B** and **F**), *MYBL2* expression (**C** and **G**), and *CDC25A* expression (**D** and **H**) in stage 1 and ≥ stage II NSCLC specimens. Pearson correlation analysis was conducted.

A Lung adenocarcinoma

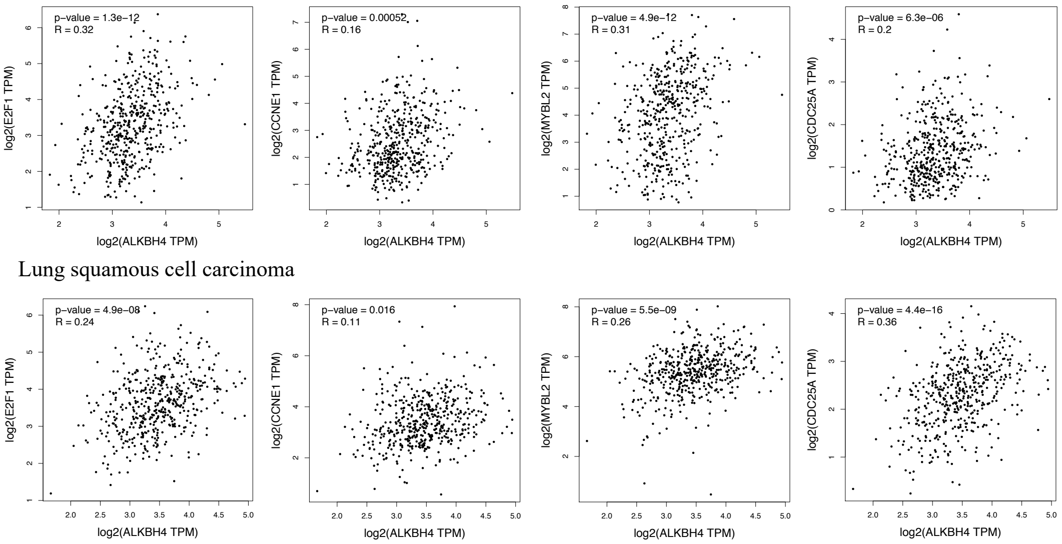

Supplementary Figure 6. The expression of *ALKBH4* and *E2F1* or E2F1-target genes has a positive correlation in large-scale cohort data of NSCLC.

Correlation analysis between ALKBH4 and *E2F1* or E2F1-target genes was performed with the expression data obtained (TPM: transcripts per million) from TCGA. **A**, Lung adenocarcinoma datasets. **B**, Lung squamous cell carcinoma datasets.

A Lung adenocarcinoma

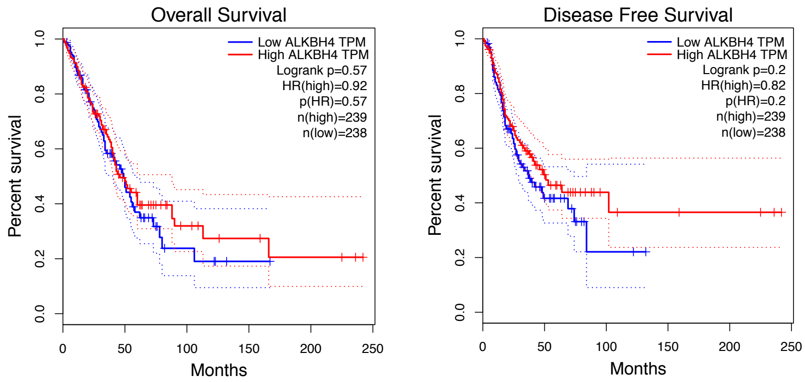

B Lung squamous cell carcinoma

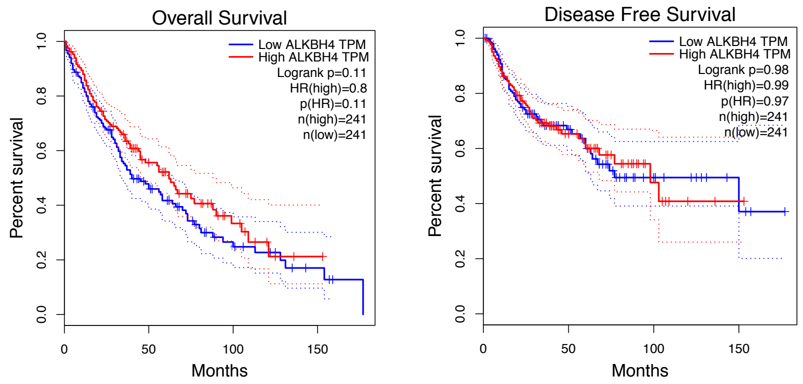

Supplementary Figure 7. Survival analysis in large-scale cohort data of NSCLC.

TCGA database analysis of ALKBH4 in patients with adenocarcinoma (**A**) or squamous cell carcinoma (**B**). Overall survival and disease-free survival analysis in patients with high (N=239) and low (N=238) ALKBH4 expression was analysed via Kaplan-Meier analysis with log-rank tests. Data were analysed using the GEPIA web server.

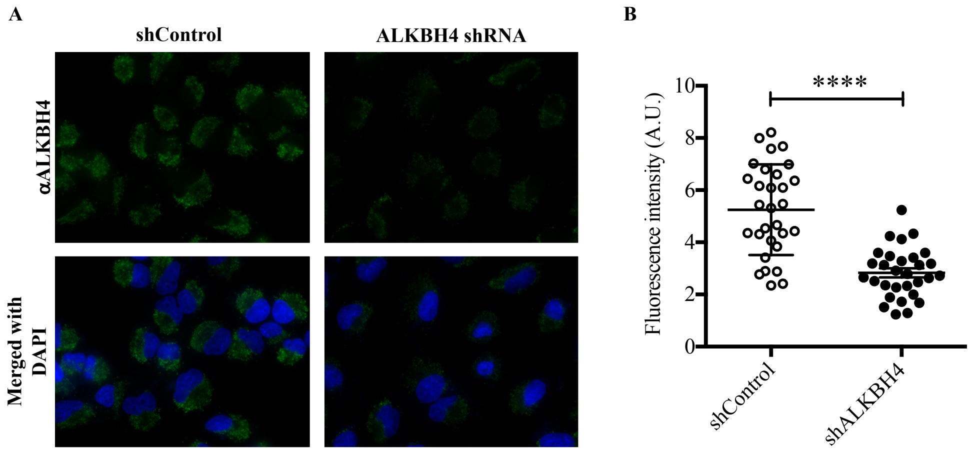

Supplementary Figure 8. Immunofluorescence staining of shControl and shALKBH4-transfected A549 cells with anti-ALKBH4 antibody.

A, For fluorescence microscopy observation, shControl or shALKBH4-transfected A549 cells were grown on a micro coverglass, fixed via incubation in 4% formaldehyde, and then permeabilized with blocking buffer containing 3% normal goat serum and 0.3% Triton X-100 in phosphate-buffered saline (PBS). The permeabilized cells were incubated with the anti-ALKBH4 antibody at 4°C overnight (1:400; Novus Biologicals, NBP2-14737), followed by incubation with fluorochrome-conjugated secondary antibody (Goat anti-rabbit IgG (H+L) highly cross-adsorbed secondary antibody, Alexa Fluor 488, A-11034, ThermoFisher Scientific) for 1 h at room temperature. Next, the coverslips were mounted onto a slide glass using Dapi Fluoromount-G (SouthernBiotech, Birmingham, AL, USA). Fluorescent images were obtained using Biozero BZ-X700 (KEYENCE). **B**, Fluorescence intensity of ALKBH4

staining for a total of 30 cells in three different fields. Data are represented as mean ± S.D. **** $p < 0.0001$ for paired *t*-test.

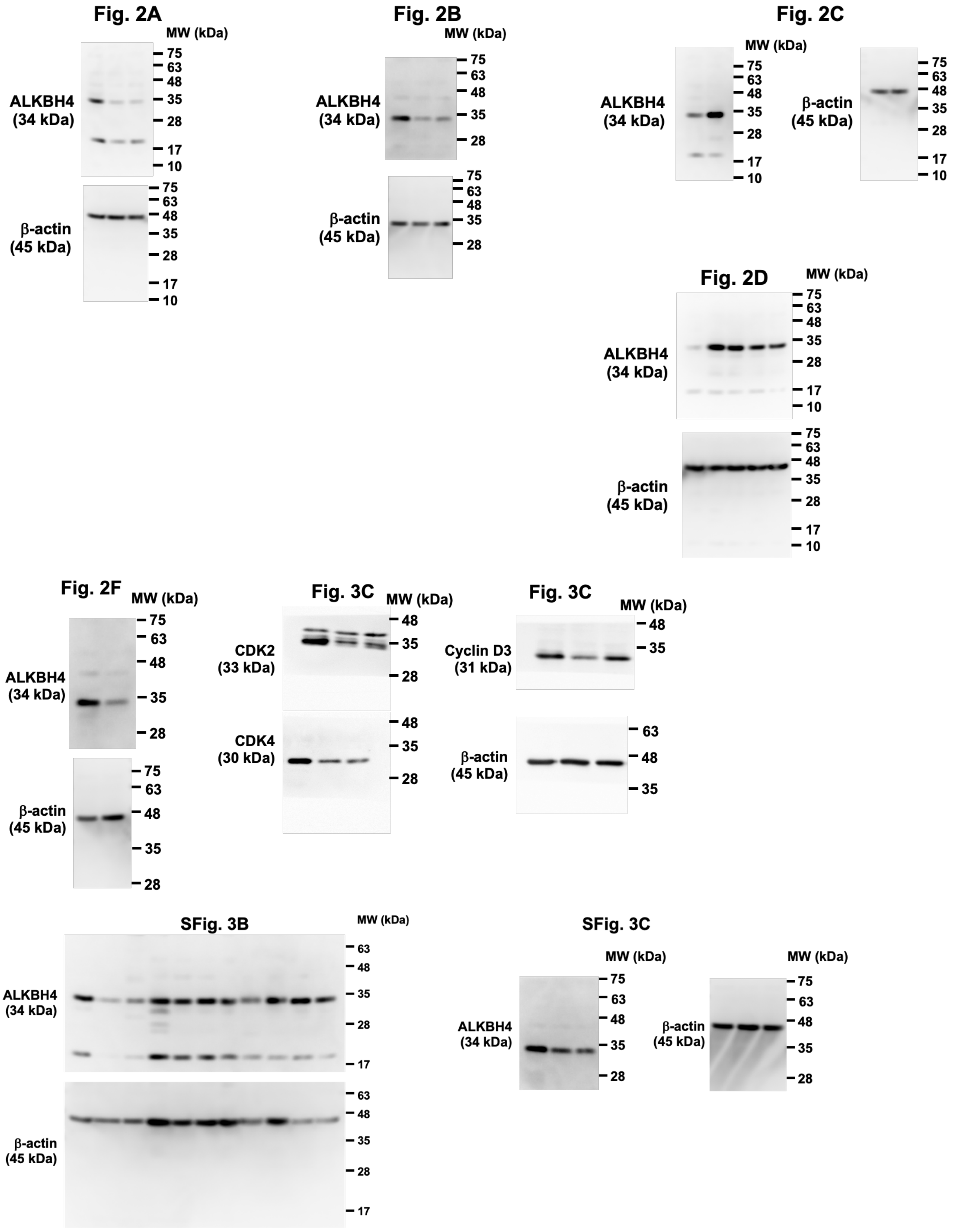

Supplementary Figure 9. Uncropped Western blot data.
